# Synthesis, Electronic Properties and Reactivity of [B_12_X_11_(NO_2_)]^2−^ (X=F–I) Dianions

**DOI:** 10.1002/chem.202003537

**Published:** 2020-10-05

**Authors:** Knut R. Asmis, Björn B. Beele, Carsten Jenne, Sebastian Kawa, Harald Knorke, Marc C. Nierstenhöfer, Xue‐Bin Wang, Jonas Warneke, Ziyan Warneke, Qinqin Yuan

**Affiliations:** ^1^ Wilhelm-Ostwald-Institut für Physikalische und Theoretische Chemie Universität Leipzig Linnéstr. 2 04103 Leipzig Germany; ^2^ Fakultät für Mathematik und Naturwissenschaften Anorganische Chemie Bergische Universität Wuppertal Gaußstr. 20 42119 Wuppertal Germany; ^3^ Physical Sciences Division Pacific Northwest National Laboratory 902 Battelle Boulevard Richland WA 99352 USA; ^4^ Leibniz Institute of Surface Engineering (IOM) Permoserstraße 15 04318 Leipzig Germany

**Keywords:** boron clusters, electronic stability, gas-phase reactions, mass spectrometry, nitro group, radical ions

## Abstract

Nitro‐functionalized undecahalogenated *closo*‐dodecaborates [B_12_X_11_(NO_2_)]^2−^ were synthesized in high purities and characterized by NMR, IR, and Raman spectroscopy, single crystal X‐diffraction, mass spectrometry, and gas‐phase ion vibrational spectroscopy. The NO_2_ substituent leads to an enhanced electronic and electrochemical stability compared to the parent perhalogenated [B_12_X_12_]^2−^ (X=F–I) dianions evidenced by photoelectron spectroscopy, cyclic voltammetry, and quantum‐chemical calculations. The stabilizing effect decreases from X=F to X=I. Thermogravimetric measurements of the salts indicate the loss of the nitric oxide radical (NO^.^). The homolytic NO^.^ elimination from the dianion under very soft collisional excitation in gas‐phase ion experiments results in the formation of the radical [B_12_X_11_O]^2−.^. Theoretical investigations suggest that the loss of NO^.^ proceeds via the rearrangement product [B_12_X_11_(ONO)]^2−^. The *O*‐bonded nitrosooxy structure is thermodynamically more stable than the *N*‐bonded nitro structure and its formation by radical recombination of [B_12_X_11_O]^2−.^ and NO^.^ is demonstrated.

## Graphical abstract

**Improved electrochemical stability**. The [B_12_X_11_(NO_2_)]^2−^ (X=F–I) dianions show an improved electrochemical stability compared to the parent [B_12_X_12_]^2−^ anions but thermally cleave NO to give very reactive [B_12_X_11_O]^2−.^ radical dianions.

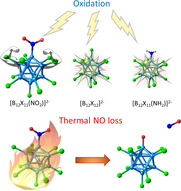

## Introduction

Perhalogenated *closo*‐dodecaborate anions [B_12_X_12_]^2−^ (X=F−I) derive from the parent icosahedral *closo*‐dodecaborate anion [B_12_H_12_]^2−^ ^[1]^ and were introduced in 1964 by Muetterties et al.^[2]^ For the last 15 years they have received increased attention after improved and simple halogenation methods were developed to make these compounds available in large quantities.[^2^, ^3^] Since then, their chemical and physical properties were investigated^[4]^ and a variety of applications were proposed: perhalogenated *closo*‐dodecaborate anions were shown to be weakly coordinating anions capable of stabilizing reactive cations in the condensed phase^[5]^ and to be suitable counterions for catalytically active cations.[^3d^, ^6^] In the solid state, they are part of superacids,^[7]^ host–guest complexes,^[8]^ and ion conductors,^[9]^ while in solution they have interesting properties for electrochemical^[4b]^ and medicinal applications.^[10]^ Their gas‐phase chemistry is unprecedented^[11]^ and most recently lead to the discovery of noble‐gas‐binding molecular anions.^[12]^

In recent years, procedures were developed to generate mono‐substituted perhalogenated *closo*‐dodecaborate anions [B_12_X_11_Y]^2−^. In principle, the functional group Y enables tuning of the anions’ properties for an anticipated application. However, synthetic procedures have only been developed so far for Y=NR_3_^+^, NH_2_, and OR (R=H, alkyl).^[13]^ Introduction of an NR_3_^+^ group reduces the total charge from −2 to −1, which leads to better solubility in organic solvents and allowed the synthesis of new ionic liquids.^[13d]^ While the functional groups Y=NH_2_, OH and OR reduce the electronic stability of the dianion, a nitro group (Y=NO_2_) is expected to increase the electronic stability of [B_12_X_11_(NO_2_)]^2−^ compared to [B_12_X_12_]^2−^. The c*loso*‐borate anions [B_*n*_H_*n*‐1_(NO_2_)]^2−^ (*n=*6, 9, 10)^[14]^ and [B_12_(OR)_11_(NO_2_)]^2−[15]^ with one nitro group have been reported, but no halogenated derivatives are known.

Herein, we report the synthesis of [B_12_X_11_(NO_2_)]^2−^ (X=F−I). The perhalogenated *closo*‐dodecaborates show exceptional stability against oxidation,[^5^, ^6^] which makes them interesting for electrochemical applications. Additionally, an unprecedented reactivity of the boron‐bound NO_2_ group was investigated, which opens the way to generate highly reactive intermediates from [B_12_X_11_(NO_2_)]^2−^ ions.

## Results and Discussion

### Synthesis and characterization of [B_12_X_11_(NO_2_)]^2−^ (X=F–I)

**Scheme 1:**
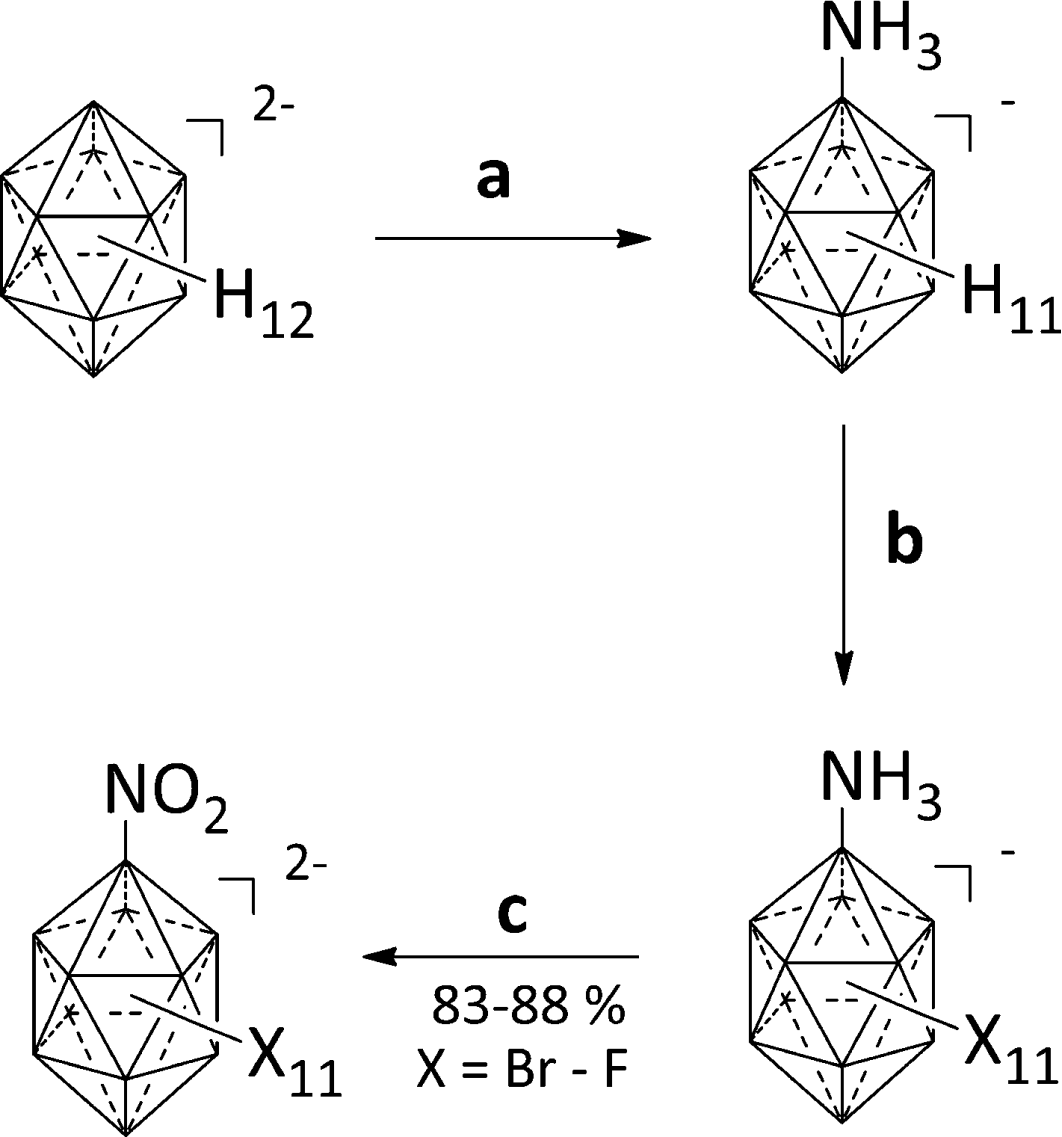
Reaction sequence to produce [B_12_X_11_(NO_2_)]^2−^ (X=F–I). (a) H_3_NOSO_3_, H_2_O,^[13a]^ (b) F_2_/N_2_ in CH_3_CN for X=F;^[16]^ SbCl_5_ for X=Cl;^[13a]^ Br in H_2_O;^[13e]^ I_2_+ICl, in C_2_H_4_Cl_2_ for X=I,[^13e^, ^17^] (c) H_2_O_2_+KOH in H_2_O, reflux.^[this work]^ The given yields correspond to the isolation of the anions as [N(*n*Bu)_4_]^+^ salts. An accurate yield for X=I could not be obtained due to the incomplete conversion. For full synthetic details including precipitation of the anions with different cations see section S2 of the Supporting Information.

Starting from the parent *closo*‐dodecaborate [B_12_H_12_]^2−^ the ammonio‐substituted perhalogenated clusters [B_12_X_11_(NH_3_)]^−^ (X=F–I) were obtained in two steps by known procedures.[^13a^, ^13e^, ^16^] Subsequently, the ammonio group was oxidized with H_2_O_2_ following a procedure used before for the synthesis of [B_12_(OH)_11_(NO_2_)]^2−^.[^15^, ^17^] For this purpose, the potassium salts of [B_12_X_11_(NH_3_)]^−^ were dissolved in an aqueous solution of hydrogen peroxide (30 %). Subsequently, potassium hydroxide was added to adjust the pH value to 8–10 (*Caution: A strong gas evolution can occur due to decomposition of H_2_O_2_. The use of a burst shield to protect from possible explosion is recommended*.). Aliquots of H_2_O_2_ were added several times a day and the reaction progress was monitored by ^11^B NMR spectroscopy. Scheme 1 illustrates the 3‐step synthesis starting from [B_12_H_12_]^2−^ and full details are given in section S2 of the Supporting Information.

**Figure 1:**
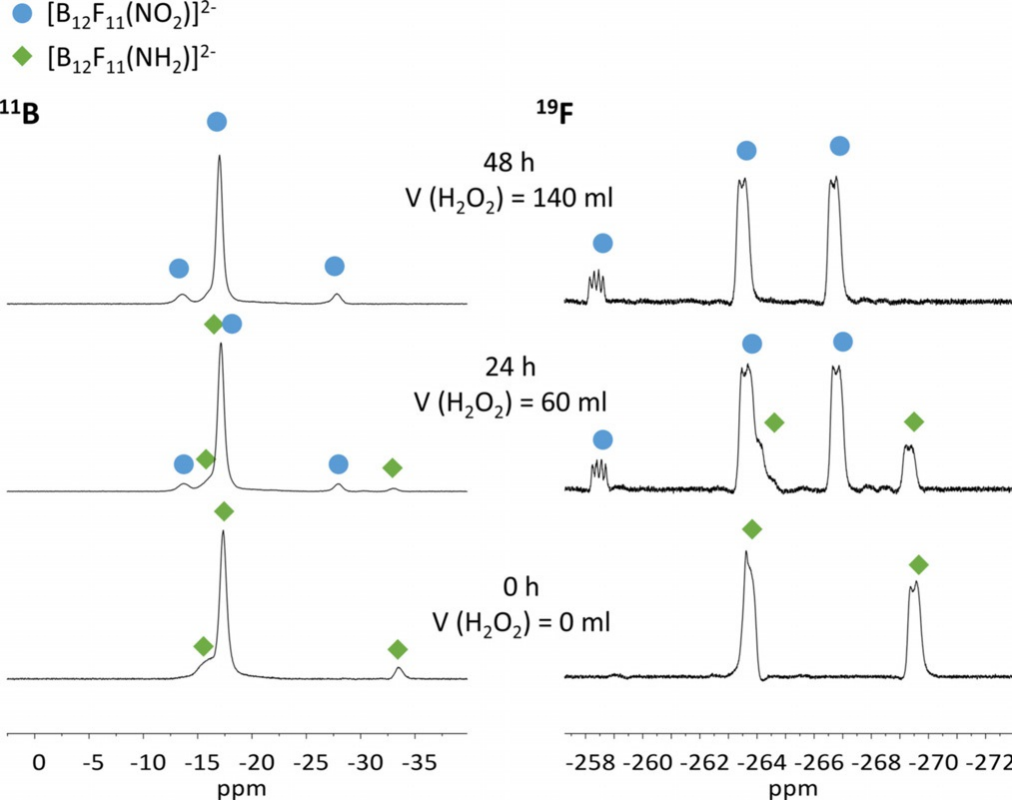
^11^B (left) and ^19^F NMR spectra (right) of the oxidation of K[B_12_F_11_(NH_3_)]^−^ with H_2_O_2_ over time. Resonances of the starting material [B_12_F_11_(NH_3_)]^−^ are marked with green diamonds and those from the product [B_12_F_11_(NO_2_)]^2−^ are labeled by blue circles.

The progress of the oxidation of the halogenated *closo*‐dodecaborates was monitored over time by NMR spectroscopy. As an example, the ^11^B and ^19^F NMR spectra for the reaction of K[B_12_F_11_(NH_3_)]^−^ with H_2_O_2_ are shown in Figure 1. The oxidation of the other derivatives was monitored in the same way and a related diagram for [B_12_Cl_11_(NO_2_)]^2−^ is given in section S2.5 of the Supporting information. At the beginning, the ^11^B NMR spectrum shows three signals in 1:10:1 ratio. After some time, a second set of three signals in 1:10:1 ratio appears in the ^11^B NMR spectrum, which can be assigned to [B_12_F_11_(NO_2_)]^2−^. ^11^B‐^11^B COSY NMR spectra helped to assign the resonances to the respective compounds. In the ^19^F NMR spectrum the integral ratio of the starting material is 6:5 and splits into a 1:5:5 ratio in the product. Obviously, the chemical shift of the fluorine atom attached to the antipodal boron atom is significantly influenced by the nitro group.

The oxidation of [B_12_F_11_(NH_3_)]^−^ requires approximately two days. In the case of X=Cl and X=Br the reaction time increases to approximately ten days and two weeks, respectively. For [B_12_I_11_(NH_3_)]^−^ a complete conversion could not be reached even after two weeks. There is a significant increase of reaction time by going from X=F to X=I. We calculated reaction energies for the oxidation of [B_12_X_11_(NH_3_)]^−^ according to Equation 1, which are highly exergonic (about 700 kJ mol^−1^ for all halogens) and only slightly decreases (by 2 %) from X=F to X=I (Table S11 of the Supporting Information). Therefore, different activation energies for the elementary reaction steps are more likely responsible for the observed change in reactivity along the halogen series than the overall reaction enthalpy.

(1) $$\left[B{}_{12}X{}_{11}\left(\mathrm{NH}{}_{3}\right)\right]{}^{-}+3\;H{}_{2}O{}_{2}+\mathrm{OH}{}^{-}\to \left[B{}_{12}X{}_{11}\left(\mathrm{NO}{}_{2}\right)\right]{}^{2-}+5\;H{}_{2}O$$

**Figure 2:**
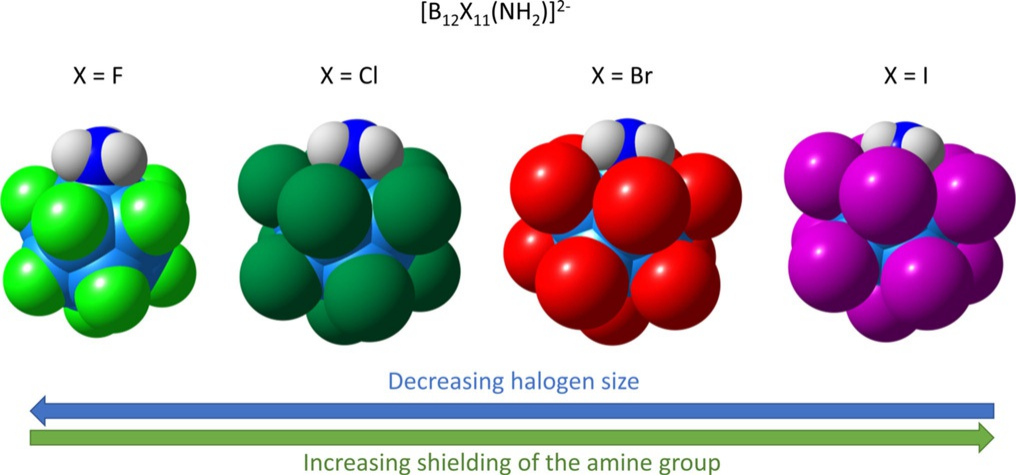
Space‐filling models based on PBE0/def2‐TZVPP geometries of [B_12_X_11_(NH_2_)]^2−^ with X=F–I. Calculated bond lengths[pm]: [B_12_F_11_(NH_2_)]^2−^: 149.6 (B−N), 138.3 (B‐F); [B_12_Cl_11_(NH_2_)]^2−^: 147.0 (B−N), 179.7 (B−Cl); [B_12_Br_11_(NH_2_)]^2−^: 146.3 (B−N), 196.3 (B−Br); [B_12_I_11_(NH_2_)]^2−^: 145.7 (B−N), 217.1 (B−I).

An attack of the nitrogen atom lone electron pair of the amine (the ammonio group is deprotonated under basic conditions) on the H_2_O_2_ molecule resulting in the formation of an amine oxide may be a simplified representation. The actual mechanism is unknown, but presumably proceeds via different steps involving cyclic transition states.^[18]^ A large halogen atom X neighbored to the NH_2_ group shields the nitrogen atom and certainly causes kinetic barriers due to steric hindrance (see Figure 2). The B−X bond is shorter than the B−N bond for X=F, but longer for X=Cl–I. In addition, basicity of the NH_2_ group decreases from X=F to X=I (see Table S10 of the Supporting Information), which may also influence the kinetics of this reaction.

### Crystal structures of [B_12_X_11_(NO_2_)]^2−^ (X=F–Br)

**Figure 3:**
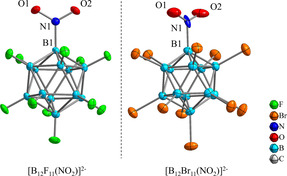
Structure of anions derived from single‐crystal X‐ray analysis of Cs_2_[B_12_F_11_(NO_2_)]⋅2 CH_3_CN and [N(*n*Bu)_4_]_2_[B_12_Br_11_(NO_2_)]⋅2 CH_3_CN. Thermal ellipsoids are drawn with 50 % probability. Selected experimental and calculated (in *italics*, PBE0/def2‐TZVPP) structural parameters (bond lengths in pm and angles in deg.) for [B_12_F_11_(NO_2_)]^2−^: N1−O1 122.5(2) *(122.2)*, N1−O2 122.3(2) *(122.2)*, B1−N1 154.1(3) *(154.9)* pm, O1‐N1‐O2 121.04(19)° *(122.4°)*, B1‐N1‐O1 119.91(19)° *(118.7°)*, B1‐N1‐O2 119.05(18)° *(118.7°)* and [B_12_Br_11_(NO_2_)]^2−^: N1−O1 111.8(5) *(121.7)*, N1−O2 115.7(5) *(121.7)*, B1−N1 165.3(6) *(155.9)* pm, O1‐N1‐O1 129.9(4)° *(123.2°)*, B1‐N1‐O1 116.0(4)° *(118.1°)*, B1‐N1‐O2 113.9(4)° *(118.1°)*.

A total of nine different crystal structures were measured for [B_12_X_11_(NO_2_)]^2−^ (X=F–Br) with different counter cations such as Cs^+^, [N(*n*Bu)_4_]^+^, [HNEt_3_]^+^, [PPh_4_]^+^, and Ba^2+^. Most of the obtained crystal structures show positional disorder of the nitro groups. The steric demand of the nitro group is similar to that of the halide substituents and we found examples where the nitro group is disordered over two, four, six, eight or twelve positions, depending on the crystal symmetry and strongly influenced by the counterion and solvent molecules in the lattice. Only for Cs_2_[B_12_F_11_(NO_2_)]⋅2 CH_3_CN and [N(*n*Bu)_4_]_2_[B_12_Br_11_(NO_2_)]⋅2 CH_3_CN crystal structures without any positional disorder of the nitro group could be obtained (Figure 3). Visualizations of the anions in the other crystal structures are shown in section S6 of the Supporting Information.

### Influence of the nitro group on the electronic properties

In organic chemistry, it is well known that the electron‐withdrawing nitro group deactivates aromatics against electrophilic attack while the electron donating amino group leads to activation. The same trend is expected for halogenated *closo*‐dodecaborate anions. To evaluate the difference in electronic stability between [B_12_X_11_(NH_2_)]^2−^, [B_12_X_12_]^2−^ and [B_12_X_11_(NO_2_)]^2−^ (X=F–I) we used photoelectron spectroscopy (PES) in the gas phase compared with quantum‐chemical calculations and cyclic voltammetry in solution.

**Figure 4:**
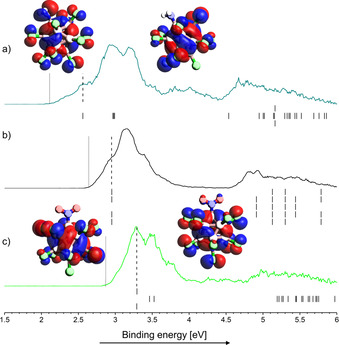
Photoelectron spectra of a) [B_12_Cl_11_(NH_2_)]^2−^, b) [B_12_Cl_12_]^2−^ and c) [B_12_Cl_11_(NO_2_)]^2−^ measured at 157 nm (7.866 eV). Dashed lines indicate vertical detachment energies (VDE) and dotted lines adiabatic detachment energies (ADE). Density of states (DOS) predicted by Hartree–Fock calculations are shown below the spectra. Note that the energy of all molecular orbitals was shifted by a constant value so that the energy of the highest occupied molecular orbital (HOMO) matches the experimental VDE. The HOMO and HOMO‐1 of [B_12_Cl_11_(NH_2_)]^2−^ and of [B_12_Cl_11_(NO_2_)]^2−^ are shown. The four highest lying orbitals of all three ions are shown in Figure S43 of the Supporting Information.

**Figure 5:**
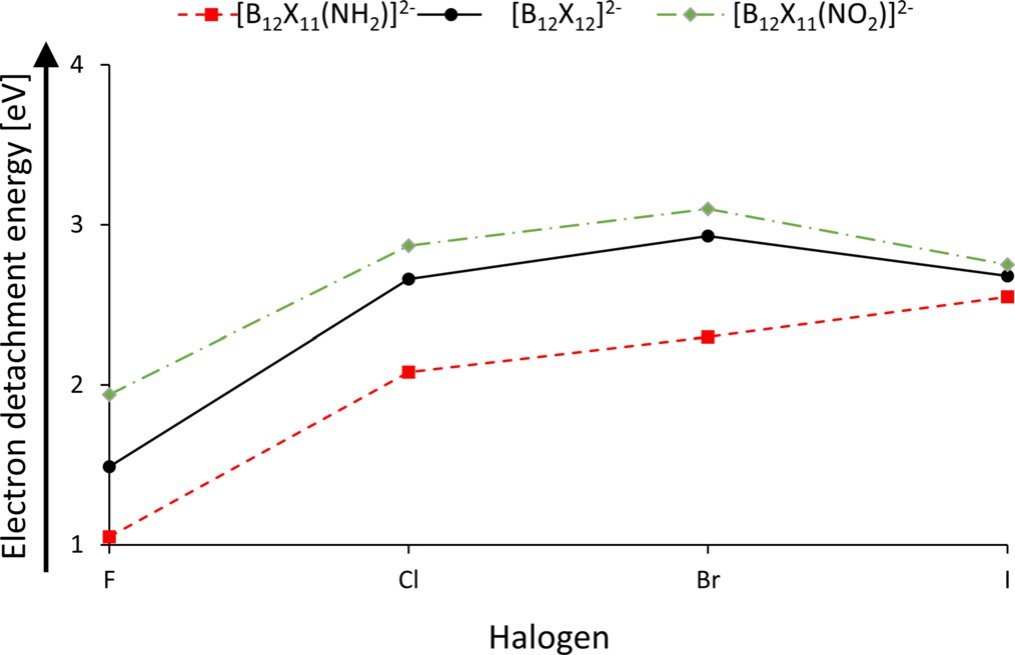
Visualization of the averaged, experimental, adiabatic electron detachment energies of the different substituted *closo*‐dodecaborate anions in the gas phase.

The photoelectron spectra measured at a laser wavelength of 157 nm for X=Cl are shown in Figure 4. All other spectra can be found in section S7 of the Supporting Information. In general, the electron donating amino group lowers the electron binding energy of the dianion in the gas phase, while the electron withdrawing nitro group leads to an increase compared to the perhalogenated dianions (Figure 5), in accord with calculated vertical (VDE) and adiabatic detachment energies (ADE) (Table 1). Hartree–Fock‐orbital energies (eigenvalues) are plotted below the spectra and shifted in energy so that the energy of the HOMO matches the VDE (Figure 4). The shift compensates for the deviation from Koopman's theorem. For high symmetry [B_12_Cl_12_]^2−^ the HOMO is four‐fold degenerated. For the lower symmetry [B_12_Cl_11_(NH_2_)]^2−^ and [B_12_Cl_11_(NO_2_)]^2−^ ions the energy of the four highest orbitals are differently affected by the functional groups. In the case of [B_12_Cl_11_(NH_2_)]^2−^, the orbital of the free electron pair of the NH_2_ group overlaps with only one of the four highest lying cluster orbitals, significantly destabilizing it energetically. In contrast, the energy of the four highest orbitals are affected by the NO_2_ group. While two orbitals are only slightly stabilized and constitute the two‐fold degenerate HOMO of [B_12_Cl_11_(NO_2_)]^2−^, a significant contribution of the oxygen orbitals is present for HOMO‐1 and HOMO‐2, which are strongly stabilized.

**Table 1:** Experimental and calculated electron detachment energies in the gas phase and oxidation potentials in SO_2_ solution. Energy differences with respect to [B_12_X_12_]^2−^ are given in brackets.^[a,b,c]^

Anion	Exp. ADE^[a]^ [eV] average	Calcd. ADE [eV] ^[b]^	Exp. VDE^[a]^ [eV] average	Calcd. VDE [eV] ^[b]^	*E* ^a^_p_^[c]^ [V]
[B_12_F_11_(NH_2_)]^2−^	1.05 (−0.44)	0.72(−0.58)	1.46 (−0.44)	1.24 (−0.54)	N/A
[B_12_F_12_]^2−^	1.49	1.30^[4b]^	1.90±0.05^[4a]^	1.78	1.78^[6]^
[B_12_F_11_(NO_2_)]^2−^	1.94 (+0.45)	1.69 (+0.39)	2.24 (+0.34)	2.12 (+0.34)	2.09 (+0.31)
[B_12_Cl_11_(NH_2_)]^2−^	2.08 (−0.58)	1.67 (−0.66)	2.36 (−0.59)	2.05 (−0.58)	N/A
[B_12_Cl_12_]^2−^	2.66	2.33^[4b]^	2.95±0.05^[4a]^	2.63	2.15^[4b]^
[B_12_Cl_11_(NO_2_)]^2−^	2.87 (+0.21)	2.52 (+0.19)	3.18 (+0.23)	2.79 (+0.16)	2.45 (+0.30)
[B_12_Br_11_(NH_2_)]^2−^	2.30 (−0.63)	1.98 (−0.66)	2.60 (−0.60)	2.33 (−0.56)	N/A
[B_12_Br_12_]^2−^	2.93	2.64^[4b]^	3.20±0.03^[4a]^	2.89	2.31^[4b]^
[B_12_Br_11_(NO_2_)]^2−^	3.10 (+0.17)	2.78 (+0.14)	3.27 (+0.07)	2.99 (+0.10)	2.39 (+0.08)
[B_12_I_11_(NH_2_)]^2−^	2.55 (−0.13)	2.24 (−0.09)	2.83 (+0.03)	2.27 (−0.07)	N/A
[B_12_I_12_]^2−^	2.68	2.33^[4b]^	2.80±0.02^[4a]^	2.34	2.1^[4b]^
[B_12_I_11_(NO_2_)]^2−^	2.75 (+0.07)	2.39 (+0.06)	2.91 (+0.11)	2.40 (+0.06)	N/A

[a] In order to obtain accurate values measurements at different wavelength were done and averaged (section S7 of the Supporting Information). [b] PBE0/def2‐TZVPP results. [c] The values of cyclovoltammetric measurements were referenced to ferrocene (*E* ^a^_p_=0.06 in SO_2_; external standard).

The electronic stabilization caused by the nitro group is strongest for X=F (+0.45 eV). Since [B_12_X_12_]^2−^ becomes electronically more stable from X=F to X=Br, the additional stabilization affected by substituting one X with NO_2_ decreases along the halogen series (X=Cl (+0.21 eV)), X=Br (+0.17 eV)). X=I constitutes a special case. While the HOMO for X=F−Br has a significant boron contribution and is influenced by the electronic effect of the substituents Y in [B_12_X_11_Y]^2−^, the HOMO for X=I is almost exclusively localized on the iodine atoms.^[4a]^ Therefore, the electronic stabilities of [B_12_I_11_NH_2_]^2−^, [B_12_I_12_]^2−^ and [B_12_I_11_NO_2_]^2−^ are almost identical, see Figure 5.

Complementary to the determination of the electronic stabilities in the gas phase, cyclovoltammetric measurements were performed in liquid sulfur dioxide solution in order to estimate the electrochemical potentials. In the past, liquid sulfur dioxide has been shown to be a suitable solvent for similar *closo*‐borate anions with very high oxidation potentials.[^4b^, ^4c^, ^19^] The cyclic voltammograms for [B_12_X_11_(NO_2_)]^2−^ (X=F–Cl) are shown in section S3 of the Supporting Information. The [B_12_X_11_(NO_2_)]^2−^ anions for X=Cl and Br show a quasi‐reversible redox process, while it is not reversible for [B_12_F_11_(NO_2_)]^2−^. Compared to [B_12_X_12_]^2−^, the oxidation potentials increase in the presence of the nitro group (see Table 1).

These results demonstrate that the electronic properties of [B_12_X_11_Y]^2−^ are determined by the choice of Y. NO_2_ constitutes a functional group which allows for further chemical modification of the anion.

### Reactivity of [B_12_X_11_(NO_2_)]^2−^ anions

**Figure 6:**
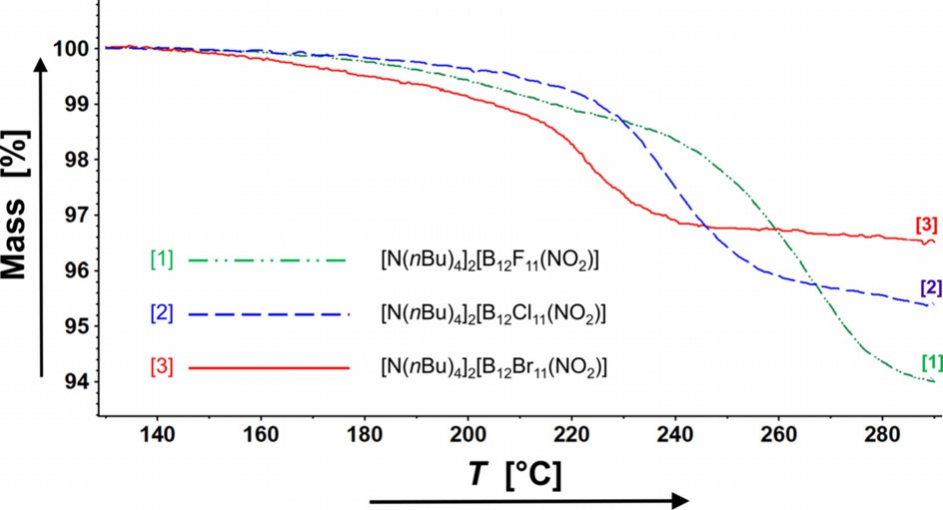
Thermogravimetric analyses of [N(*n*Bu)_4_]_2_[B_12_X_11_(NO_2_)] (X=F, Cl, Br) with a heating rate of 5 K min^−1^.

*Closo*‐borate anions are three‐dimensional aromatics.^[20]^ Therefore, it is reasonable to assume that the NO_2_ group may be further functionalized by established procedures known for nitrobenzene derivatives. For instance, the reaction with nascent hydrogen obtained by acidifying a heterogenic zinc solution, which is known to reduce aromatic NO_2_ groups, generates [B_12_X_11_(NH_3_)]^−^ in good yields (section S5 of the Supporting Information). Surprisingly, we observed a thermally induced reaction for [B_12_X_11_(NO_2_)]^2−^, which is known for nitrobenzene only as a side reaction: In a simultaneous thermogravimetric (TG) and differential scanning calorimetric (DSC) measurement of [N(*n*Bu)_4_]_2_[B_12_X_11_(NO_2_)] (X=F–Br) in the temperature range from 25 to 350 °C, a step in the TG analysis with onset temperatures between 214 and 248 °C was observed (Figure 6). The detected mass losses are in accord with NO^.^ loss from the anion (see Table 2 for experimental and calculated values). The onset temperatures in the TG analyses and the reaction enthalpies obtained by the DSC analyses decrease from [B_12_F_11_(NO_2_)]^2−^ to [B_12_Br_11_(NO_2_)]^2−^ showing that the stability of the nitro group decreases with increasing halogen size from X=F−Br. Decomposition of the organic counterion does not start below 400 °C.

**Table 2:** Summary of the thermogravimetric and differential scanning calorimetric measurements.

Compound	*T*_onset_	Mass loss [%]		Exp. reaction enthalpy
	[°C]	exp.	calcd.	[kJ mol^−1^]
[N(*n*Bu)_4_]_2_[B_12_F_11_(NO_2_)]	248	3.8±0.5	3.5	−79
[N(*n*Bu)_4_]_2_[B_12_Cl_11_(NO_2_)]	226	3.1±0.5	2.9	−71
[N(*n*Bu)_4_]_2_[B_12_Br_11_(NO_2_)]	214	1.9±0.5	2.0	−62

**Figure 7:**
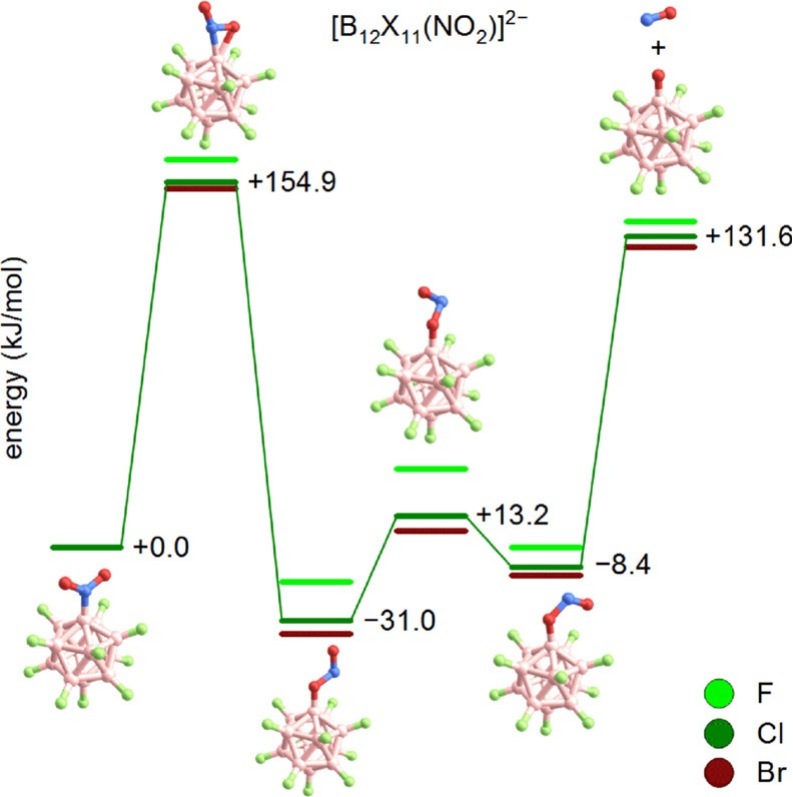
Calculated (B3LYP+GD3BJ/def2‐TZVPP) reaction path way for NO^.^ elimination from [B_12_X_11_(NO_2_)]^2−^ (X=F–Br).

The loss of the radical NO^.^ as an exclusive reaction at fairly low temperatures from an even electron molecule with an *N*‐bonded NO_2_ group is surprising. Quantum‐chemical calculations suggest that the *N*‐bonded NO_2_ group is transformed via an *η* ^2^‐*N*,*O*‐bonded transition state (+157 kJ mol^−1^) into an *O*‐bonded nitrosooxy moiety (R‐ONO), which lies several kJ mol^−1^ lower in energy than the *N*‐bonded isomer, according to DFT analysis. Subsequently, the ONO‐substituted cluster easily eliminates an NO^.^ molecule (Figure 7).

The change in transition state and reaction enthalpies along the halogen series are qualitatively in agreement with the trend observed in thermogravimetry. A driving force for the rearrangement may be the formation of the strong boron‐oxygen bond. For comparison, for nitrobenzene the corresponding transition state is 100 kJ mol^−1^ higher in energy and the nitrosooxy isomer C_6_H_5_(ONO) is less energetically stable than nitrobenzene^[21]^ (Figure S41 of the Supporting Information). The remaining [B_12_X_11_O]^2−.^ radical possesses an oxygen‐localized unpaired electron (section S8 and Figure S44 of the Supporting Information) and must be highly reactive. To generate this intermediate, a sample of Cs_2_[B_12_F_11_(NO_2_)] was heated in an open vessel to 300 °C for 40 min and the residue was analyzed by IR, NMR and MS methods and compared to the starting material (spectra are shown in section S4 of the Supporting Information). The ^11^B NMR spectrum changed from a signal in 1:10:1 ratio to a significantly different pattern in 11:1 ratio (Figure S25 of the Supporting Information), indicating that the B_12_ unit is still present, but has been chemically modified. In the IR spectrum (Figure S24 of the Supporting Information), the signals assigned to the antisymmetric NO_2_ stretch and to the B−N stretch have disappeared and a broad O−H band becomes visible. This is confirmed by the detection of [B_12_F_11_(OH)]^2−^ in the mass spectrometric analysis (Figure S26 of the Supporting Information). Since the heated vessel was not protected from ambient molecules like H_2_O, [B_12_X_11_(OH)]^2−^ is formed by H^.^ abstraction.

**Figure 8:**
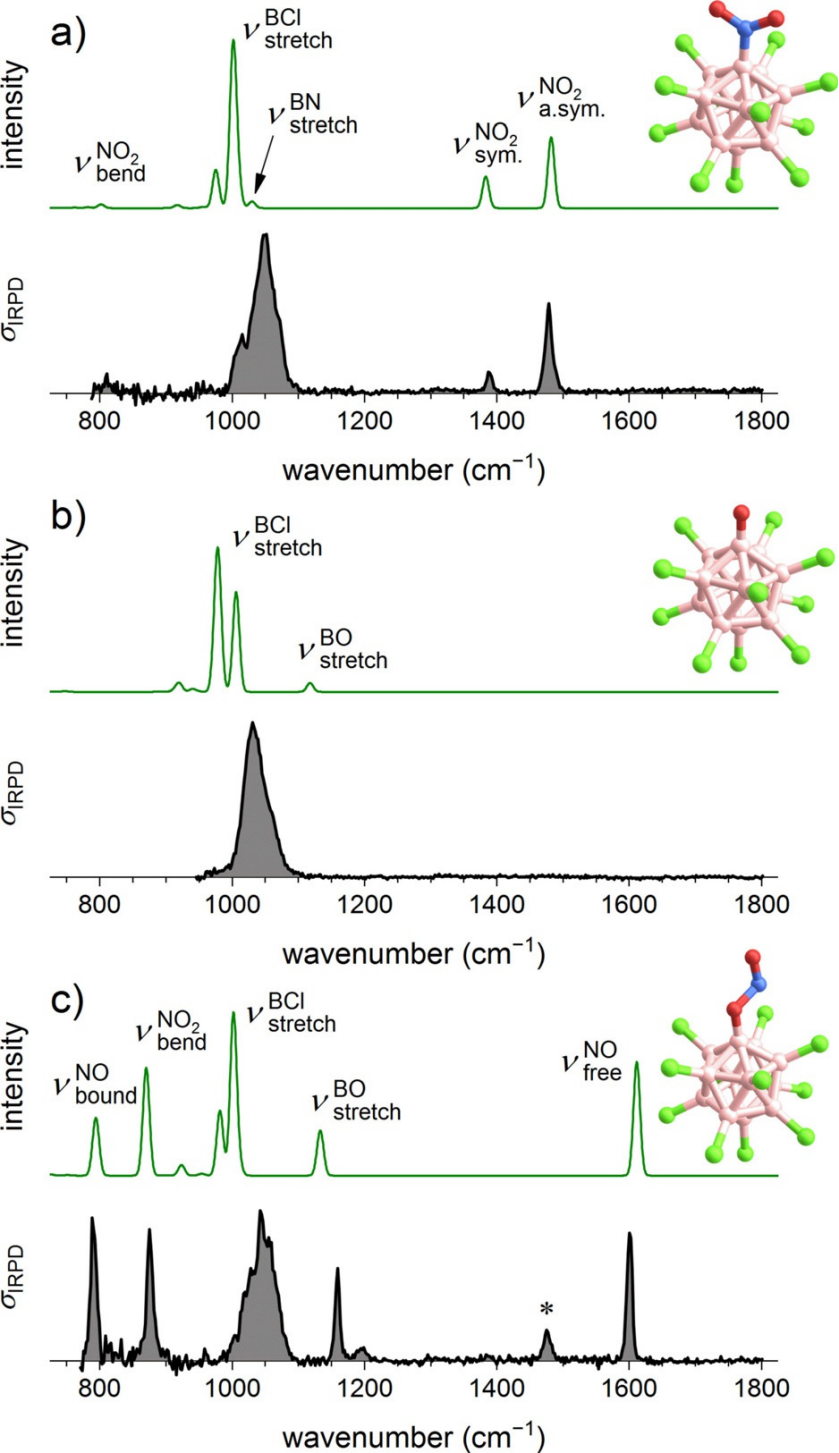
B3LYP+GD3BJ/def2‐TZVPP harmonic IR spectra (green) and experimental IRPD spectra of N_2_ messenger‐tagged ions (black) a) [B_12_Cl_11_(NO_2_)]^2−^, b) [B_12_Cl_11_O]^2−.^, and c) [B_12_Cl_11_(ONO)]^2−^. The ion [B_12_Cl_11_(ONO)]^2−^ was generated by radical recombination of [B_12_Cl_11_O]^2−.^ and NO^.^. Small amounts of the isobaric [B_12_Cl_11_(NO_2_)]^2−^ ions due to incomplete fragmentation (for further information see section S2 of the supporting Information) could not be avoided and are responsible for the spectral band marked with an asterisk. See Table S13 of the Supporting Information for band positions and their assignment.

For a direct evidence of the proposed NO^.^ loss, we aimed for the observation of [B_12_X_11_O]^2−.^. Due to the ions’ high reactivity, potential reaction partners need to be eliminated. Usually, the observation of isolated radical dianions is challenging, because autodetachment of an electron occurs. However, the exceptional electronic stability of halogenated *closo*‐borate anions can prevent the dianionic radical from electron emission in the gas phase. Electrospray ionization (ESI) was used to transfer [B_12_X_11_(NO_2_)]^2−^ into the gas phase of our mass spectrometers. Even at very “soft” conditions (low collisional excitation, see parameters in section S2 of the Supporting Information), ions with *m*/*z* 15 smaller than [B_12_X_11_(NO_2_)]^2−^ were observed. Two experiments were performed to differentiate the ion [B_12_X_11_O]^2−.^ from the isobar [B_12_X_11_(NH_2_)]^2−^. (1) High resolution mass spectrometry of [B_12_I_11_O]^2−.^ distinguishes this ion from [B_12_I_11_(NH_2_)]^2−^ which was measured for comparison (Figure S55 of the Supporting Information). (2) Structural information on the gaseous ions [B_12_Cl_11_(NO_2_)]^2−^ and [B_12_Cl_11_O]^2−.^ were obtained from vibrational spectra using infrared photodissociation spectroscopy (IRPD).^[22]^ The comparison of the IRPD spectrum of [B_12_Cl_11_(NO_2_)]^2−^ with harmonic IR spectra (Figure 8 a) from DFT calculations shows that the B−N (and not the B−O) bound isomer is present in the gas phase. The symmetric and antisymmetric nitro stretching bands are observed at 1388 cm^−1^ and 1478 cm^−1^, respectively. In contrast, the IRPD spectrum of [B_12_Cl_11_O]^2−.^ reveals no IR‐active modes of significant intensity above the dominant absorption band at 1032 cm^−1^ (Figure 8 b), which is associated with B−Cl stretching vibrations, coupled to B_12_‐cage deformation modes.^[23]^ The absence of any NH_2_ signals (Figure S46 of the Supporting Information) underlines the assignment and evidences the observation of [B_12_Cl_11_O]^2−.^. Note, the global minimum‐energy structure, the nitrosooxy isomer [B_12_Cl_11_(ONO)]^2−^, is not stabilized following collision‐induced dissociation (CID) of [B_12_Cl_11_(NO_2_)]^2−^. The energy necessary to overcome the energy barrier for the NO_2_ rearrangement results in bond cleavage and NO^.^ loss. However, the [B_12_Cl_11_(ONO)]^2−^ ion, for which so far no synthetic procedure exists, can be generated by gas phase radical recombination of [B_12_Cl_11_O]^2−.^ with NO^.^. Its IRPD spectrum, shown in Figure 8 c, is different from the other two and exhibits a characteristic free N=O stretching band at 1600 cm^−1^.

**Figure 9:**
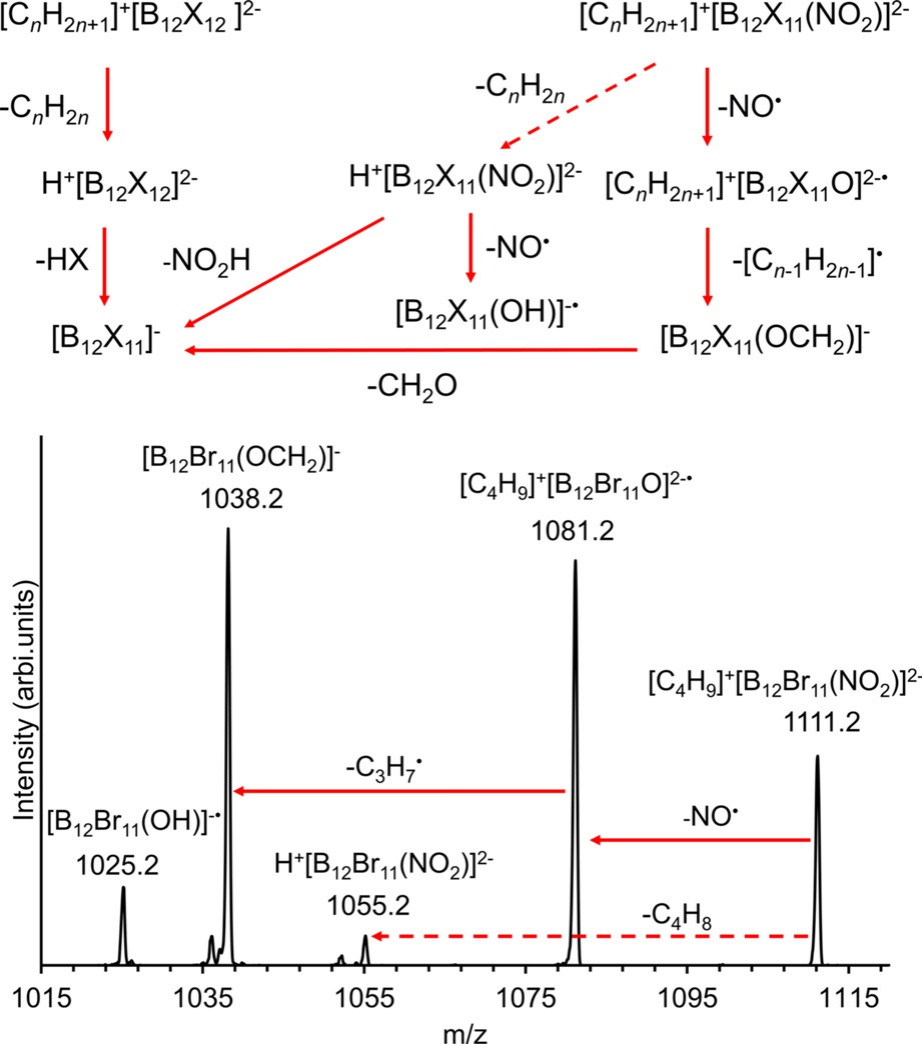
a) Fragmentation scheme of the ion pairs [C_*n*_H_2*n*+1_]^+^[B_12_X_11_(NO_2_)]^2−^ and [C_*n*_H_2*n*+1_]^+^[B_12_X_12_]^2−^. While CID of the latter exclusively results in the loss of C_*n*_H_2*n*_, this is only a side reaction for [C_*n*_H_2*n*+1_]^+^[B_12_X_11_(NO_2_)]^2−^ (indicated by the dashed arrow) and the loss of the NO^.^ is more pronounced. The subsequent loss of [C_*n*‐1_H_2*n*‐1_]^.^ transforms the fragment into the even electron ion [B_12_X_11_(OCH_2_)]^−^. The fragmentation of both ion pairs finally results in [B_12_X_11_]^−^ (for detailed MS^n^ analysis confirming the shown pathway for X=Br and for exemplary spectra for X=Cl, see section S9 of the Supporting Information). b) CID mass spectrum of the isolated [C_4_H_9_]^+^[B_12_Br_11_(NO_2_)]^2−^ ion pair showing the NO^.^ and C_3_H_7_^.^ loss as main fragmentation pathways and the C_4_H_8_ loss as a side reaction (dashed arrow).

The formation and reactivity of [B_12_Cl_11_O]^2−.^ was further investigated by gas‐phase experiments in the presence of counterions. Following an established procedure, alkyl cations [C_*n*_H_2*n*+1_]^+^ (*n=*3, 4) were bound to the dianion by fragmentation of a tetraalkylammonium counterion.^[11a]^ For the fully perhalogenated clusters, CID of the anionic ion pair [C_*n*_H_2*n*+1_]^+^[B_12_X_12_]^2−^ results in the loss of an alkene and formation of the strong acid H^+^[B_12_X_12_]^2−^ (see Figure 9 a for a fragmentation scheme). In contrast, the equivalent reaction is only observed as a side‐reaction for [C_*n*_H_2*n*+1_]^+^[B_12_X_11_(NO_2_)]^2−^. The loss of NO^.^ from the ion pair is more pronounced. The resulting radical dianion appears to attack the cation. The next fragmentation step is the loss of the radical [C_*n*‐1_H_2*n*‐1_]^.^, which transforms the anion into the even electron system [B_12_X_11_(OCH_2_)]^−^ (Figure 9 b). The pronounced differences in the fragmentation behavior of [C_*n*_H_2*n*+1_]^+^[B_12_X_12_]^2−^ and [C_*n*_H_2*n*+1_]^+^[B_12_X_11_(NO_2_)]^2−^ underline the strong tendency for the loss of NO^.^ and reveal interesting perspectives for reactions with the [B_12_X_11_O]^2−.^ dianions.

## Conclusions

The halogenated [B_12_X_11_(NO_2_)]^2−^ dodecaborates were obtained in high purity and yield by oxidation of the corresponding amines. This new class of halogenated *closo*‐dodecaborates shows improved electrochemical stability compared to the common [B_12_X_12_]^2−^ anions. Their ability to release nitric oxide by thermal treatment gives access to the dianionic oxygen‐bound radical [B_12_X_11_O]^2−.^, which might be a very useful precursor for the chemical modification of the halogenated *closo*‐dodecaborates.

## Experimental Section

Numbering scheme, experimental details and spectroscopic data, cyclic voltammetry, thermal NO^.^ cleavage, reduction of the nitro group by nascent hydrogen, crystal structures, photoelectron spectroscopy, quantum‐chemical calculations, gas phase chemistry are reported in the Supporting Information.

Deposition numbers 2009135, 2009136, 2009137, 2009138, 2009139, 2009140, 2009141, 2009142, and 2009143 contain the supplementary crystallographic data for this paper. These data are provided free of charge by the joint Cambridge Crystallographic Data Centre and Fachinformationszentrum Karlsruhe Access Structures service.

## Conflict of interest

The authors declare no conflict of interest.

## Supporting information

As a service to our authors and readers, this journal provides supporting information supplied by the authors. Such materials are peer reviewed and may be re‐organized for online delivery, but are not copy‐edited or typeset. Technical support issues arising from supporting information (other than missing files) should be addressed to the authors.

Supplementary
