# Supplementary material for: Synthesis, Electronic Properties and Reactivity of [B12X11(NO2)]2− (X=F–I) Dianions
Source: Chemistry. 2020 Oct 5;26(64):14594–601. doi: 10.1002/chem.202003537 (PMC7756457; doi:10.1002/chem.202003537)
Supplement: Supplementary file 1 — Supplementary [file CHEM-26-14594-s001.pdf]

Chemistry–A European Journal

Supporting Information

Synthesis, Electronic Properties and Reactivity of $[B_{12}X_{11}(NO_2)]^{2-}$ (X = F–I) Dianions

Knut R. Asmis,[a] Björn B. Beele,[b] Carsten Jenne,*[b] Sebastian Kawa,[a] Harald Knorke,[a] Marc C. Nierstenhöfer,[b] Xue-Bin Wang,[c] Jonas Warneke,*[a, d] Ziyan Warneke,[a] and Qinqin Yuan[c]

Table of contents

S1 Numbering scheme 3

S2 Experimental details and spectroscopic data 3

S2.1 Synthesis and spectroscopic characterization of $M_2[B_{12}F_{11}(NO_2)]$ 7

S2.2 Synthesis and spectroscopic characterization of $M_2[B_{12}Cl_{11}(NO_2)]$ (M = Cs^+, Ba^{2+}, PPh_4^+, $[HNEt_3]^+$, $[N(n\text{Bu})_4]^+$) 12

S2.3 Synthesis and spectroscopic characterization of $M_2[B_{12}Br_{11}(NO_2)]$ 15

S2.4 Synthesis and spectroscopic characterization of $K_2[B_{12}I_{11}(NO_2)]$ 18

S2.5 Monitoring the reaction progress of the oxidation over time 20

S2.6 Comparison of the ^{11}B NMR spectra of $[B_{12}X_{11}(NO_2)]^{2-}$ (X = F, Cl, Br) 21

S3 Cyclic voltammetry 22

S4 Thermal NO Cleavage 23

S5 Reduction of the nitro group by nascent hydrogen 26

S6 Crystal structures 28

S7 Photoelectron spectroscopy 35

S8 Quantum-chemical calculations 40

S8.1 Calculated energies of all optimized structures 44

S8.2 Coordinates of all optimized structures 45

S9 Gas phase chemistry 57

S10 References 64

S1 Numbering scheme

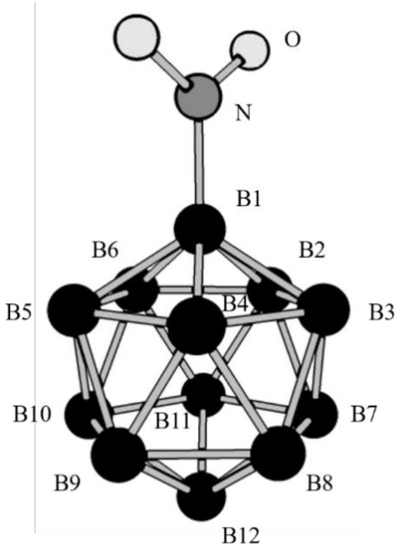

Figure S1: Numbering scheme for the $[B_{12}X_{11}NO_2]^{2-}$ anions (X = F, Cl, Br, I) according to IUPAC.

S2 Experimental details and spectroscopic data

General remarks: All prepared compounds are air and moisture stable. $K_2[B_{12}H_{12}]$ was prepared by a known procedure starting from $Na[BH_4]$ (Acros, 98+ %) and I_2 (Roth, 99.8 %) in diglyme (Fluka, 99.5 %). $K[B_{12}H_{11}NH_3]$ was prepared from $K_2[B_{12}H_{12}]$ and Hydroxylamine-*O*-sulfonic acid in water.[1] $K[B_{12}Cl_{11}NH_3]$ was obtained by chlorination of $K_2[B_{12}H_{11}NH_3]$ with $SbCl_5$.[2] The other halogenated anions $[B_{12}X_{11}(NH_3)]^-$ (X = F, Br, I) are synthesized by known procedures.[3][4]The reagents H_2O_2 (35 %, Carl Roth), KOH (Grüssing, 85 %), $[N(n\text{Bu})_4]Br$ (EGA-Chemie), $[PPh_4]Cl$ (abcr), $Et_3N{\cdot}HCl$ (abcr), CsCl (Carl Roth), hydrochloric acid (37 %, Acros Organics) were commercially available and were used without further purification. The solvents acetonitrile (AWD Scientific), D_2O (Deutero), and CD_3CN (Deutero) were used without further purification. The solvent liquid sulfur dioxide (Schick, 99.8 %) was dried over CaH_2 (Merck, 90 – 95 %) and distilled prior to use.

***Quantum-chemical calculations*:** All quantum-chemical calculations were performed on the PBE0/def2-TZVPP and B3LYP+GD3BJ/def2-TZVPP levels of theory as implemented in the program package Gaussian 16.[5] Zero Point Energies (ZPE) were included to determine the given relative energies. The harmonic approximation was used for the calculated vibrational spectra. A scaling factor of 0.968 was determined by comparing the experimental and computational NO stretching vibrations. Calculated structures, mapped van der Waals spheres, and orbitals were visualized by the programs Chemcraft[6] and a self-written visualization

program in Mathematica.[7] In order to ensure that the relative order of isomers is method independent, we used additionally the DFT functional HSE06 and basis sets aug-cc-pVTZ and def2-TZVPPD.

Vibrational spectroscopy: IR spectra of the non-air sensitive compounds were recorded on a Bruker VERTEX 70 spectrometer equipped with a diamond ATR attachment in the range of 400–4000 cm^{-1}. Raman spectra of the non-air sensitive compounds were recorded in flame sealed cappilaries on a Bruker MultiRam spectrometer in the range of 100–3600 cm^{-1}.

NMR spectroscopy: ^{1}H, ^{11}B, ^{13}C, ^{19}F and ^{31}P NMR spectra were measured on Bruker Avance 400 MHz NMR and Bruker Avance 600 MHz spectrometers in 5 mm NMR tubes equipped with J. Young valves at 298 K. Chemical shifts are given with respect to Me_4Si (^{1}H, ^{13}C), $BF_3{\cdot}OEt_2$ (^{11}B), $CFCl_3$ (^{19}F) and H_3PO_4 (85%) (^{31}P).

Mass spectrometry: Mass spectra (ESI-MS) were recorded on a Bruker Daltonics micrOTOF instrument equipped with an Agilent 1100 series (LC) liquid chromatograph and direct infusion. The solid samples were dissolved in acetonitrile.

IRPD spectroscopy: IRPD experiments were performed using the Leipzig 6 K ion trap triple mass spectrometer.[8] Gaseous $[B_{12}Cl_{11}(NO_2)]^{2-}$ ions were produced in a nanospray ion source from a 0.2 mmol L^{-1} solution of $[N(C_4H_9)_4]_2[B_{12}Cl_{11}(NO_2)]$ in methanol. The ion beam is transferred into vacuum via a two-stage differentially pumped chamber. By applying a 300 V difference between the chambers, collision-induced dissociation can take place to form the radical $[B_{12}Cl_{11}O]^{2-\bullet}$. The anions are than collimated in a gas-filled radio frequency (RF) ion guide. To induce reactions with the ions, the reaction gas is added to the buffer gas (Helium). Subsequently, the ions are mass-selected using a quadrupole mass filter and focused in a RF ring-electrode ion trap, held at a temperature in-between 6 and 300 K using a closed-cycle helium cryostat. To allow for continuous ion loading and ion thermalization, the trap is continuously filled with buffer gas, either He (for bare ions) or N_2 (for messenger-tagged ions). Many collisions of the trapped ions with the buffer gas provide gentle cooling of the internal degrees of freedom close to the ambient temperature. At sufficiently low ion trap temperatures, ion-messenger complexes $[B_{12}Cl_{11}(NO_2)]^{2-}(N_2)_z$ are formed via three-body collisions.[9] Every 100 ms, all ions are extracted from the ion trap and focused both temporally and spatially into the center of the extraction region of an orthogonally-mounted double-focusing reflectron time-of-flight (TOF) mass spectrometer, which is operated in the IR^1MS^2 mode. The ion packet is

accelerated into the reflectron stage, ions spread out in space according to their mass-to-charge ratio (*m*/*z*) and are refocused at the initial extraction region. Prior to reacceleration towards the MCP detector, ions with a particular *m*/*z* value are irradiated by a properly timed, widely wavelength tunable IR laser pulse (bandwidth: 2.5 cm^{-1}), supplied by an optical parametric oscillator/amplifier (LaserVision: OPO/OPA/$AgGaSe_2$) laser system.[10] All parent and photofragment ions are then accelerated toward an MCP detector. An IRPD spectrum is measured by continuously scanning the laser wavelength, which is monitored online using a HighFinesse WS6-600 wavelength meter, with a scan speed such that an averaged TOF mass spectrum (over 50 laser shots) is obtained every 2 cm^{-1}. The photodissociation cross section σ_{IRPD} is determined as described previously.[8]

Additional remark: For the spectroscopic investigation of $[B_{12}Cl_{11}(ONO)]^{2-}$ instrumental settings were optimized to produce large intensities of $[B_{12}Cl_{11}O]^{2-\bullet}$ by skimmer-induced fragmentation. The ion beam comprises still small amounts of the precursor $[B_{12}Cl_{11}(NO_2)]^{2-}$. The reaction of $[B_{12}Cl_{11}O]^{2-\bullet}$ with $NO^{\bullet}$ was initiated before mass selection. Therefore, the ion beam contains mainly the B-O-bound isomer, slightly contaminated with the remaining B-N-bound $[B_{12}Cl_{11}(NO_2)]^{2-}$.

CID Experiments: Tetrabutylammonium salts of $[B_{12}X_{11}(NO_2)]^{2-}$ (X = Cl, Br, I) were dissolved in acetonitrile to a final concentration of ~10^{-6} mol L^{-1}. The solution was injected using a syringe pump into the inlet of an ion trap mass spectrometer (LTQ Orbitrap XL (Thermo Fisher Scientific GmbH, Bremen, Germany) or Bruker Esquire 3000 (Bruker Daltonik, Bremen, Germany) at a flow rate of ~3 µL min^{-1}. CID experiments were performed by isolating the ion of interest and subsequently fragmenting it by applying an adjustable AC voltage. Helium is used as a collision gas at a typical activation time of 30 ms. Ions with a specific *m/z* value were isolated using: 1) a wide isolation window of 10 *m/z* to include the natural broad isotopic pattern of the precursor ion and identify the corresponding product ions; 2) a narrow isolation window of ~1 *m/z* to unambiguously determine the mass of a neutral loss in a CID spectrum.

Photoelectron spectroscopy: The photoelectron (PES) experiments were performed with an apparatus consisting of an electrospray ionization sourse (ESI), a temperature-controlled cryogenic ion trap and a magnetic bottle time-of-flight (TOF) photoelectron spectrometer. Similiar cryogenic traps have been used for cold gas ion spectroscopy study. An acetonitrile solution solution of different cation salt (K^+, $[HNEt_3]^+$, $[N(nBu)_4]^+$) of $[B_{12}X_{11}NH_2]^{2-}$ (X = F, Cl, Br, I) and $[B_{12}X_{11}(NO_2)]^{2-}$ (X = F, Cl, Br, I) was used for electrospray. The amino substituted

clusters $[B_{12}X_{11}(NH_2)]^{2-}$ were obtained from the ammonio substituted $[B_{12}X_{11}(NH_3)]^-$ (X = F-I) anions by deprotonation with potassium hydroxide to have the identical twofold negative charge on all investigated ions. The ions generated by ESI were guided by quadrupole ion guides into the ion trap, where they were accumulated and cooled for 20−100 ms by collisions with cold buffer gas (20% H_2 balanced in helium) at 20 K, before being transferred into the extraction zone of a TOF mass spectrometer. The cooling of the anions to 20 K improved spectral energy resolution and eliminated the possibility of the appearance of peaks in the PE spectra, due to hot bands. The ions were then mass selected and maximally decelerated before being photodetached with a laser beam. Photons of three different wavelengths were used: 266 nm (4.661 eV) photons from a Nd:YAG laser; 193 nm (6.424 eV) and 157 nm (7.866 eV) from an excimer laser. All lasers were operated at a 20 Hz repetition rate, with the ion beam off at alternating laser shots, affording shot-to-shot background subtraction. Photoelectrons were collected with ca. 100% efficiency with the magnetic bottle and analyzed in a 5.2 m long electron flight tube. The recorded TOF photoelectron spectrum was converted into an electron kinetic energy spectrum by calibration with the known photoelectron spectra of I^- and $[OsCl_6]^{2-}$. The electron binding energy (EBE) was obtained by subtracting the electron kinetic energy from the energy of the detaching photons. The energy resolution was about 2%, i.e., ~20 meV for 1 eV kinetic energy electrons.

Cyclic Voltammetry: Cyclic voltammograms were obtained at temperatures below 243 K in 1-4 mm solutions in liquid sulfur dioxide. The samples contained 0.1m $[N(n Bu_4)][AsF_6]$ as the supporting electrolyte. The CVs were executed using a Metrohm PGSTAT 101potentiostat. The cell design utilized a conventional three-electrode setup with a 2.0 mm diameter platinum working electrode, a platinum-wire auxiliary, and a silver wire quasi-reference electrode. The CVs were obtained at a scan rate of 0.1 V s^{-1}. All potentials are reported versus the operative formal potential $E^0_{Fc+/0}$ for the $Fc^{0/+}$ redox couple. However, Fc could not be used as an internal standard owing to the strong oxidizing properties of the analytes, but ferrocene by itself behaves ideally in liquid SO_2. Therefore, the spectra were calibrated for the $Fc^{0/+}$ redox pair against the Ag wire pseudo-reference electrode as described before.[11]

Thermal analysis: Thermogravimetric analysis (TGA) and Differential scanning calorimetry (DSC) measurements were simultaneously carried out using a Netzsch STA 449 F5 Jupiter instrument. Experiments were conducted in 40 μL aluminium crucibles, which were closed with aluminium lids. Samples were heated from 25°C to 450°C with a heating rate of 5 K min^{-1} in a nitrogen atmosphere applying a constant nitrogen flow of 25 ml min^{-1} during the experiment.

***Crystal structure determinations*:** Single crystals suitable for X-ray diffraction were obtained by slow removal of the solvent from the reaction mixture or by diffusion of diethyl ether into a saturated solution in acetonitrile. The single crystal X-ray diffraction studies were performed on an Oxford Diffraction Gemini E Ultra diffractometer equipped with a 2 K × 2 K EOS CCD area detector and a four-circle kappa goniometer using MoKα (0.71073 Å) radiation at 150 K. The crystals were mounted onto a cryo loop using fluorinated oil and frozen in the cold nitrogen stream of the goniometer. Details of the crystallographic data collection and the refinement parameters can be found in Tables S5 and S6. The structures were solved by direct methods (SHELXT)[12] using the program package OLEX2[13]. Subsequent least-squares refinement on F^2 (SHELXL)[14] located the positions of the remaining atoms in the electron density maps. All non-hydrogen atoms were refined with anisotropic displacement parameters. Hydrogen atoms were placed in calculated positions using a riding model. The data were corrected for absorption. Deposition numbers CCDC-2009135-2009143 contain the supplementary crystallographic data for this paper. These data are provided free of charge by the joint Cambridge Crystallographic Data Centre and Fachinformationszentrum Karlsruhe Access Structures service www.ccdc.cam.ac.uk/structures.

S2.1 Synthesis and spectroscopic characterization of $M_2[B_{12}F_{11}(NO_2)]$

$K[B_{12}F_{11}(NH_3)]$ (1.00 g, 2.55 mmol, 1.0 eq.) was dissolved in 20 ml of hydrogen peroxide (35 %). The pH value was set to 9 by adding potassium hydroxide. The solution was heated to reflux (the oil bath temperature was set to 120 °C) under intense stirring (**caution**: gas and heat evolution!) for 48 h. Three times a day a 20 ml portion of hydrogen peroxide (35 %) was added and the pH value was adjusted again if necessary. The reaction progress was monitored by ^{11}B NMR spectroscopy. After 48 hours stirring was continued at elevated temperature until a test with potassium starch paper for remaining hydrogen peroxide was negative. Subsequently, the mixture was allowed to reach room temperature and the pH was adjusted to 7 by adding hydrochloric acid and filtered. A solution of $[N(n\text{Bu})_4]Br$ (1.64 g, 5.09 mmol, 2.0 eq.) in 5 ml of water was added dropwise to the filtrate under intense stirring to precipitate the product (Alternatively, CsCl was used to obtain the cesium salt.). The suspension was stirred for 20 minutes and then filtered. The isolated precipitate was washed with water (3 × 10 ml), dissolved in acetonitrile, and filtered again. Evaporation of the solvent yielded $[N(n\text{Bu})_4]_2[B_{12}F_{11}(NO_2)]$ (1.95 g, 2.24 mmol, 88 %) as a white solid.

NMR data for $[N(n\mathrm{Bu})_4]_2[B_{12}F_{11}(NO_2)]$: ^{1}H NMR (600.27 MHz, CD_3CN, 298 K): δ = 1.01 (t, $^{3}J_{HH}$ = 9.0 Hz, 12H, $[N(n\mathrm{Bu})_4]^+$), 1.39 (m, 8H, $[N(n\mathrm{Bu})_4]^+$), 1.64 (m, 8H, $[N(n\mathrm{Bu})_4]^+$), 3.12 (m, 8H, $[N(n\mathrm{Bu})_4]^+$). ^{11}B{^{1}H} NMR (128.38 MHz, CD_3CN, 298 K): δ = -13.5 (s, 1B, *B*12), -16.9 (s, 10B, *B*(2-11)), -27.7 (s, 1B, *B*1). ^{13}C NMR (150.95 MHz, CD_3CN, 298 K): δ = 13.8 (s, $[N(n\mathrm{Bu})_4]^+$), 20.3 (m, $[N(n\mathrm{Bu})_4]^+$), 24.3 (s, $[N(n\mathrm{Bu})_4]^+$), 59.4 (m, $[N(n\mathrm{Bu})_4]^+$). ^{19}F NMR (376.42 MHz, CD_3CN, 298 K): δ = -259.4 (q, $^{1}J_{FB}$ = 60.2 Hz, 1F, *F*-B12), -264.4 (m, 5F, *F*-B(7–11)), 267.2 (m, 5F, *F*-B(2–6)).

IR (diamond ATR) of $Cs_2[B_{12}F_{11}(NO_2)]$: $\tilde{\nu}$ = 1461 (s, ν_{as} (NO_2)), 1390 (m, ν_s (NO_2)), 1316 (w), 1221 (vs, ν_{as} (B-F)), 1065 (vw), 1035 (vw), 829 (vw), 729 (vs, δ (B-F)), 657 (vs), 559 (vw), 540 (vw), 497 (w), 454 (vw), 418 (vw).

Raman of $Cs_2[B_{12}F_{11}(NO_2)]$: $\tilde{\nu}$ = 2955 (w), 1475 (w, ν_{as} (NO_2), 1466 (w, ν_{as} (NO_2), 1398 (s, ν_s (NO_2)), 1317 (w), 1069 (w), 830 (w), 660 (w), 454 (s), 421 (vs), 394 (vs), 318 (m), 302 (m), 231 (w), 178 (w), 145 (w), 116 (s, sh).

ESI-MS [neg. mode, m/z]: found = 627.3797 (calc. = 627.3802), $[(N(n\mathrm{Bu})_4)\ (B_{12}F_{11}(NO_2))]^-$

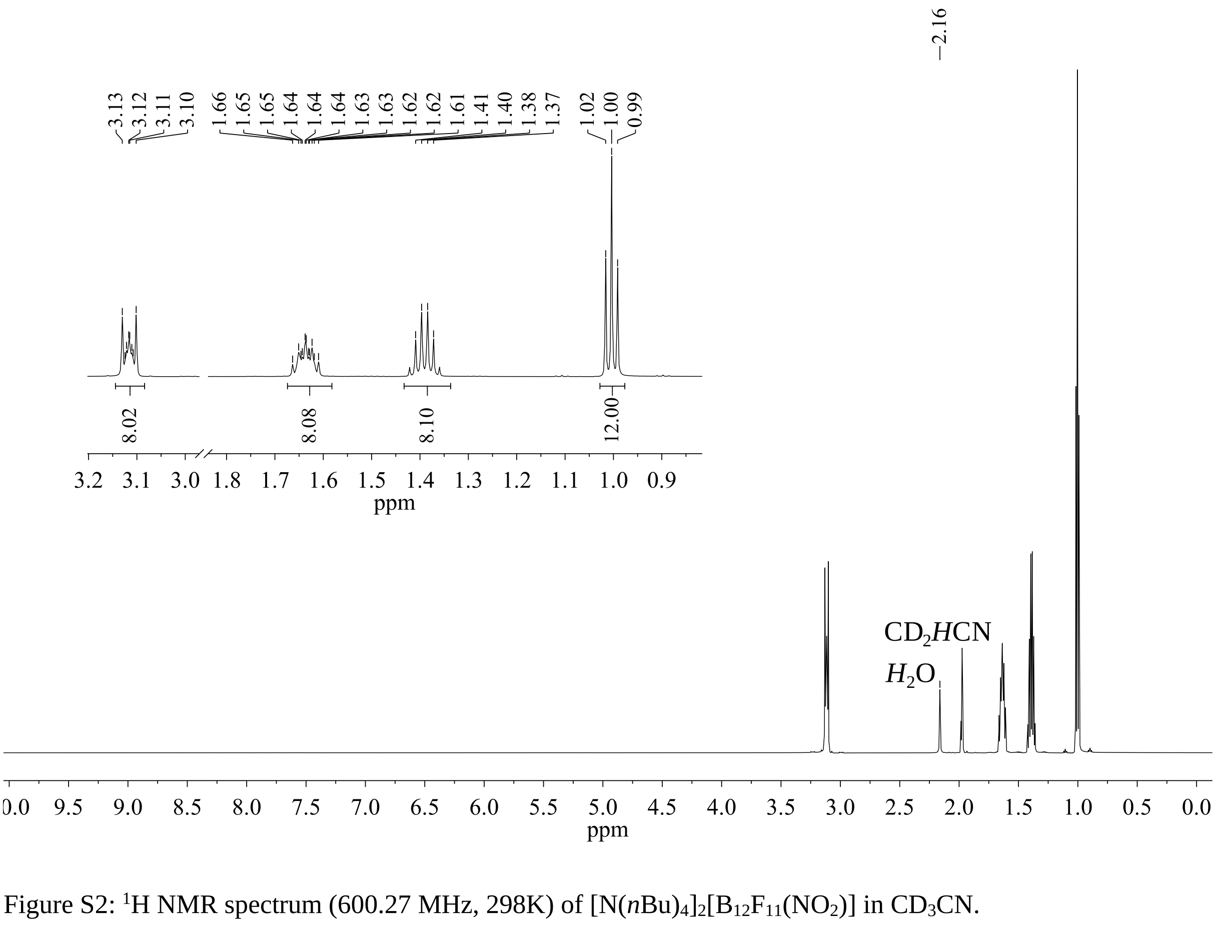

Figure S2: ^{1}H NMR spectrum (600.27 MHz, 298K) of $[N(n\mathrm{Bu})_4]_2[B_{12}F_{11}(NO_2)]$ in CD_3CN.

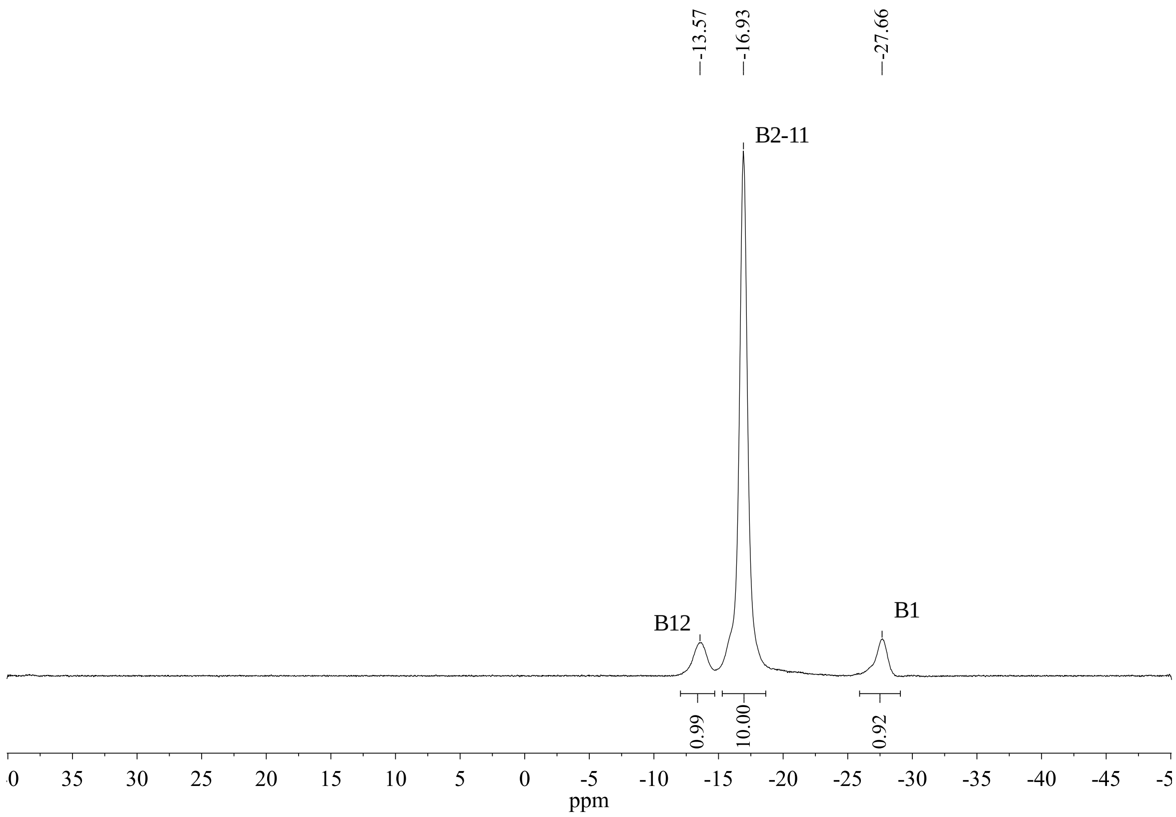

Figure S3: ^{11}B NMR spectrum (128.38 MHz, 298K) of $[N(nBu)_4]_2[B_{12}F_{11}(NO_2)]$ in CD_3CN.

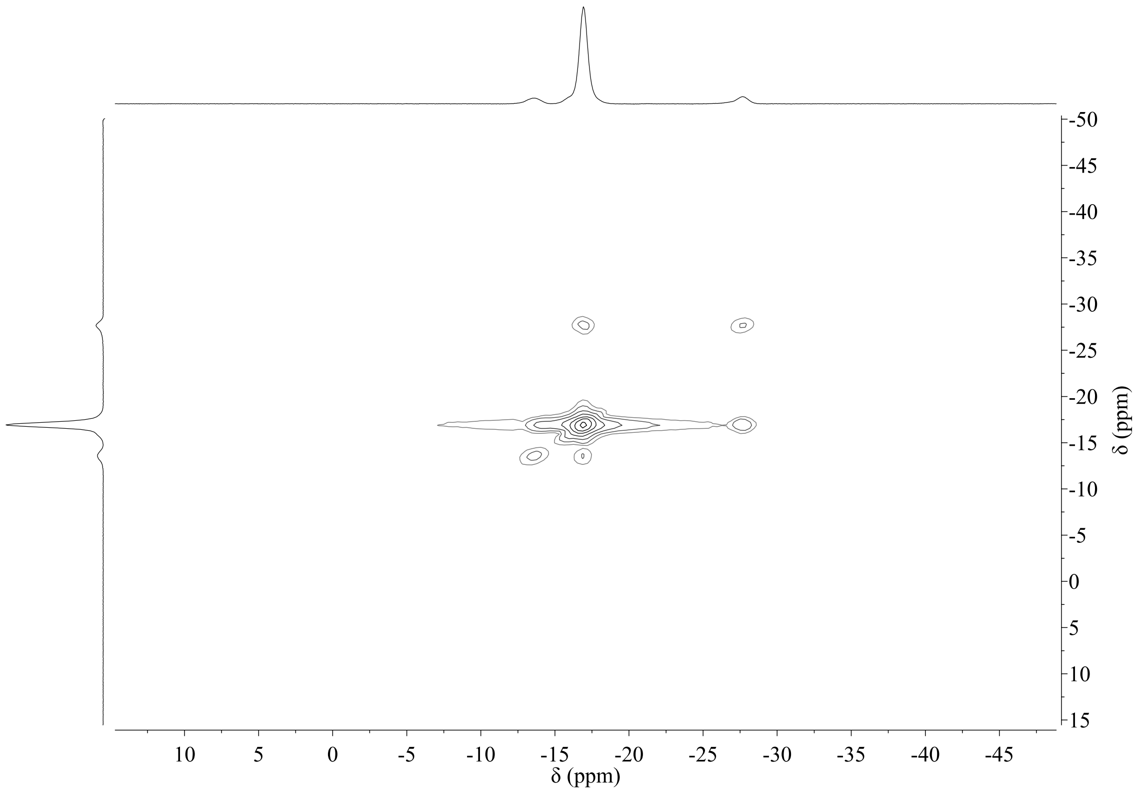

Figure S4: ^{11}B-^{11}B-COSY NMR spectrum (128.38 MHz, 298K) of $[N(nBu)_4]_2[B_{12}F_{11}(NO_2)]$ in CD_3CN.

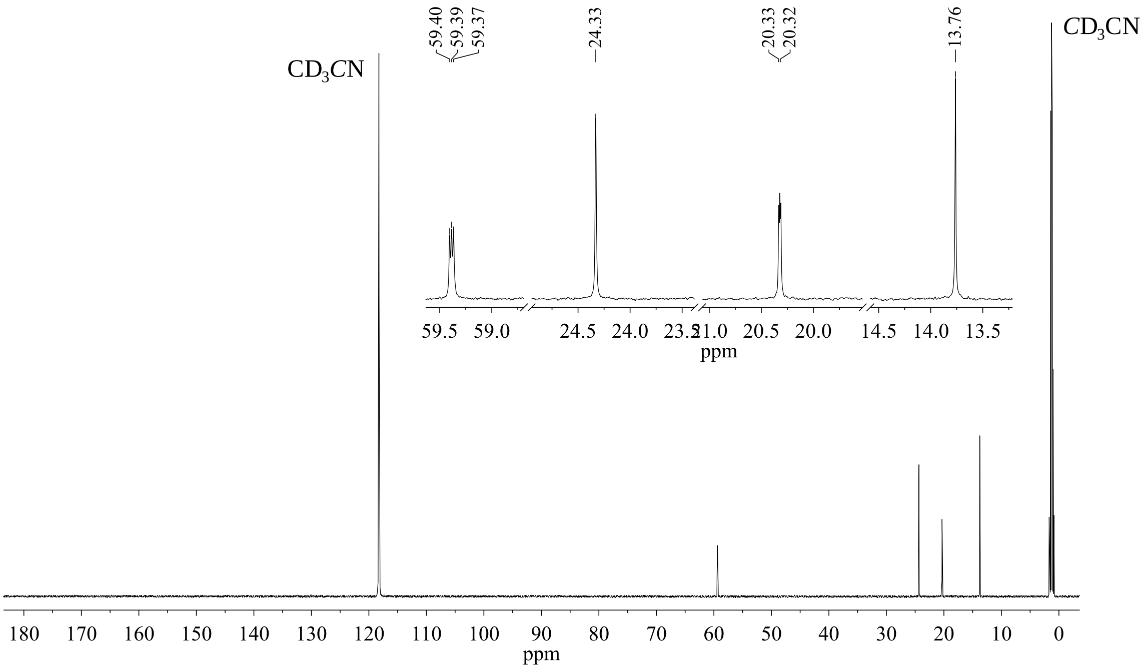

Figure S5: ^{13}C NMR spectrum (150.95 MHz, 298K) of $[N(nBu)_4]_2[B_{12}F_{11}(NO_2)]$ in CD_3CN.

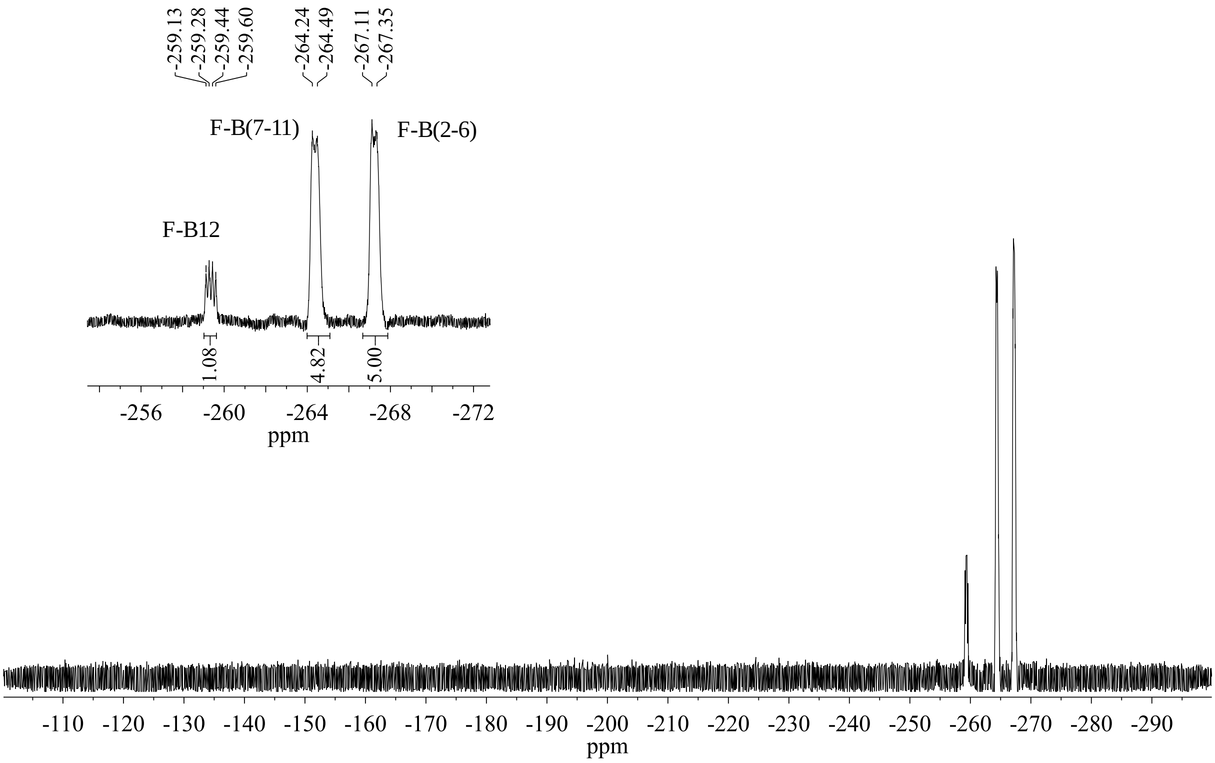

Figure S6: ^{19}F NMR spectrum (376.42 MHz, 298K) of $[N(nBu)_4]_2[B_{12}F_{11}(NO_2)]$ in CD_3CN.

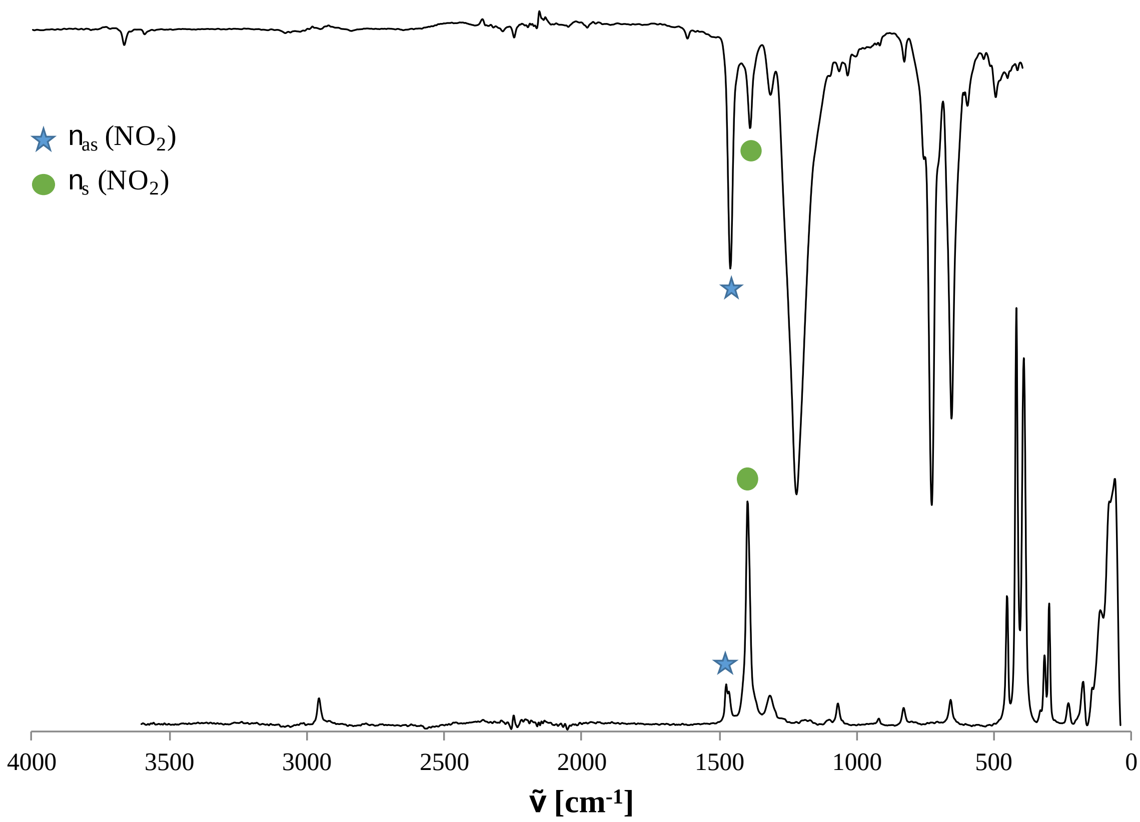

Figure S7: IR- (top) and Raman-spectra (bottom) of $Cs_2[B_{12}F_{11}(NO_2)]$.

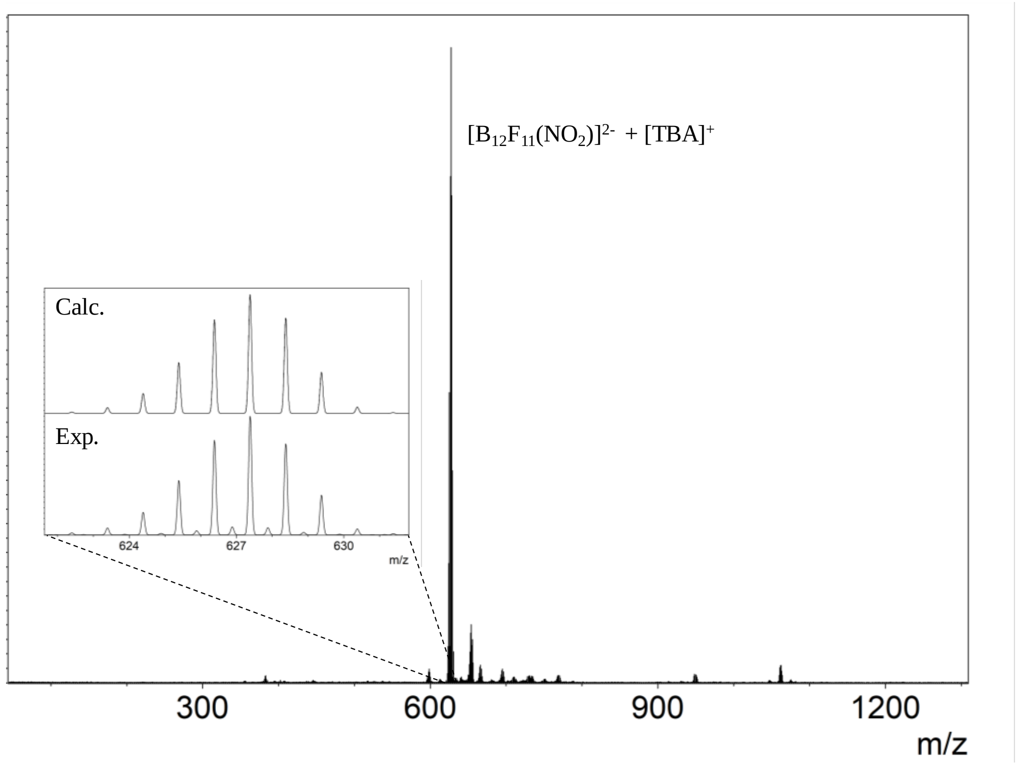

Figure S8: ESI-mass spectrum (negative mode) of $[N(n\text{Bu})_4]_2[B_{12}F_{11}(NO_2)]$. The simulated mass spectrum is shown above.

S2.2 Synthesis and spectroscopic characterization of $M_2[B_{12}Cl_{11}(NO_2)]$ (M = Cs^+, Ba^{2+}, PPh_4^+, $[HNEt_3]^+$, $[N(nBu)_4]^+$)

$K[B_{12}Cl_{11}(NH_3)]$ (1.50 g, 2.6 mmol, 1.0 eq.) was dissolved in 20 ml of hydrogen peroxide (35 %). The pH value was set to 9 by adding potassium hydroxide. The solution was heated to reflux (the oil bath temperature was set to 120 °C) under intense stirring (caution: gas and heat evolution!) for 10 d. Three to four times a day another 20 ml portion of hydrogen peroxide (35 %) was added and the pH value was adjusted again, if necessary. The reaction progress was monitored by ^{11}B NMR spectroscopy. After 10 days and a total amount of 780 ml hydrogen peroxide was added the mixture was continued stirring at elevated temperature until a test with potassium starch paper for remaining hydrogen peroxide was negative. Subsequently, the mixture was allowed to reach room temperature and the pH was adjusted to pH 7 by adding hydrochloric acid and filtered. A solution of $[N(nBu)_4]Br$ (1.64 g, 5.09 mmol, 2.0 eq.) in 5 ml of water was added dropwise to the filtrate under intense stirring to precipitate the product (Alternatively, CsCl to give the cesium salt, $Et_3N\cdot HCl$ to give the $[Et_3NH]^+$ salt, or $[PPh_4]Cl$ to give the $[PPh_4]^+$ salt may be used. The Ba^{2+} salt was obtained by reacting the $[Et_3NH]^+$ salt with $Ba(OH)_2$). The suspension was stirred for 20 minutes and then filtered. The isolated solid residue was washed with water (3 × 10 ml), dissolved in acetonitrile, and filtered again. Evaporation of the solvent yielded $[(nBu)_4N]_2[B_{12}Cl_{11}(NO_2)]$ (2.33 g, 2.22 mmol, 85 %) as a white solid.

NMR data for $[N(nBu)_4]_2[B_{12}Cl_{11}(NO_2)]$: ^1H and ^{13}C spectra are identical to those for $[N(nBu)_4]_2[B_{12}F_{11}(NO_2)]$. $^{11}B\{^1H\}$ NMR (128.38 MHz, CD_3CN, 298 K): δ = -10.1 (s, 1B), -12.0 (s, 5B), -13.5 (s, 6B).

IR (diamond ATR) of $Cs_2[B_{12}Cl_{11}(NO_2)]$: $\tilde{\nu}$ = 1477 (s, ν_{as} (NO_2)), 1395 (m, ν_s (NO_2)), 1032 (vs, ν_{as} (B-Cl)), 1009 (s, sh, ν_{as} (B-Cl)), 812 (m, δ (NO_2)), 695 (vw), 657 (w), 624 (w), 568 (w, sh), 544 (s), 520 (vs, δ (B-Cl)), 458 (m), 412 (w, sh).

Raman of $Cs_2[B_{12}Cl_{11}(NO_2)]$: $\tilde{\nu}$ = 1483 (w, ν_{as} (NO_2), 1400 (w, ν_s (NO_2)), 323 (w, sh), 303 (vs), 270 (w), 132 (s).

ESI-MS [m/z]: found = 282.8825 (calc. = 282.8585) $[B_{12}Cl_{11}(NO_2)]^{2-}$, 807.0454 (807.0498) $[(N(nBu)_4)(B_{12}Cl_{11}(NO_2))]^-$.

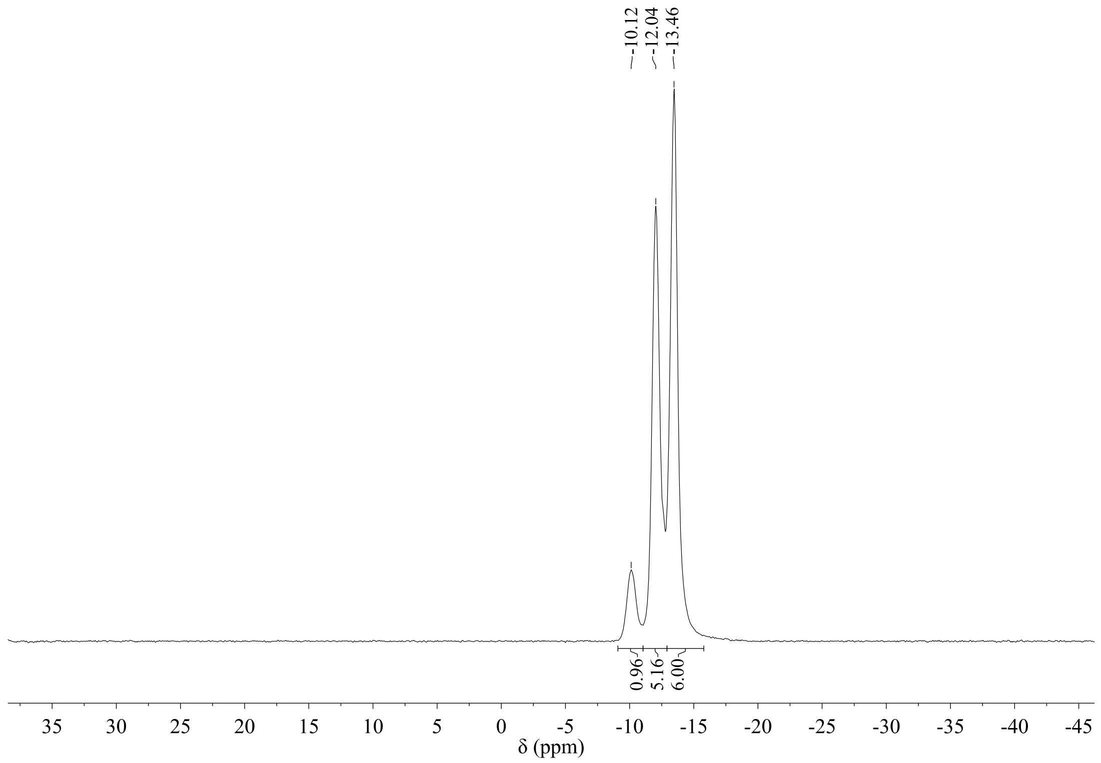

Figure S9: ^{11}B NMR spectrum (128.38 MHz, 298K) of $[HNEt_3]_2[B_{12}Cl_{11}(NO_2)]$ in CD_3CN.

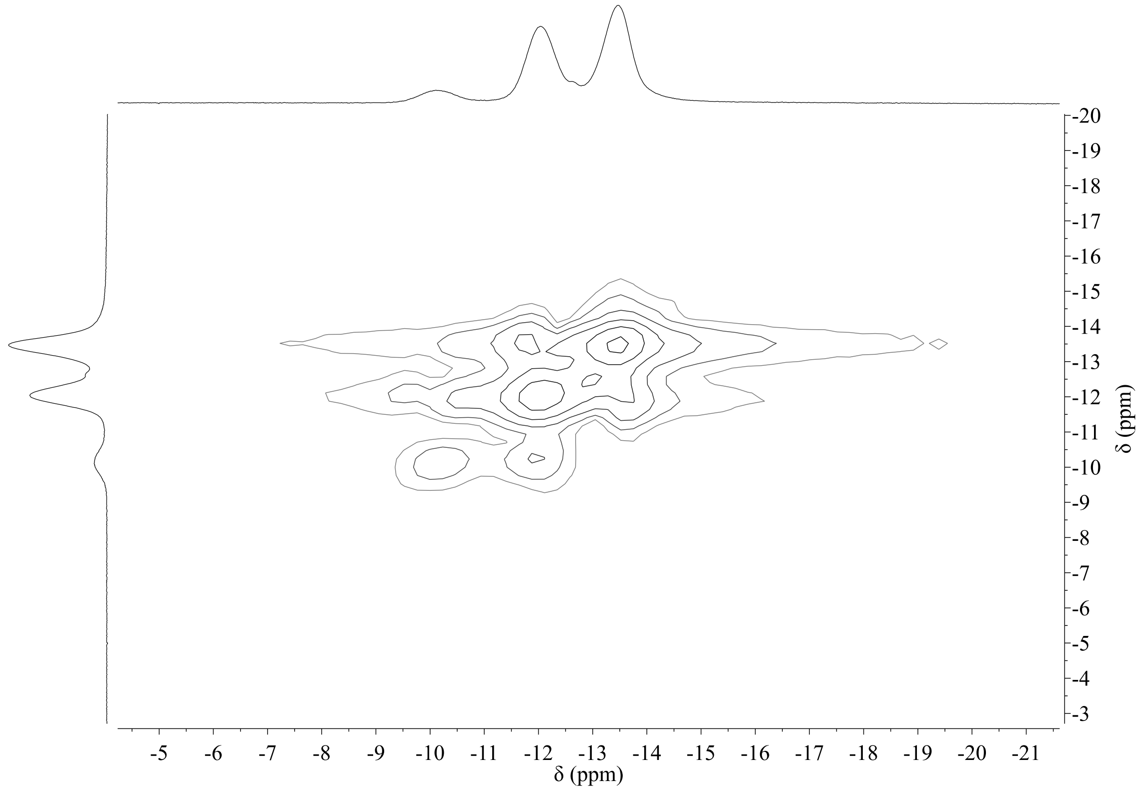

Figure S10: ^{11}B-^{11}B NMR spectrum (128.38 MHz, 298K) of $[HNEt_3]_2[B_{12}Cl_{11}(NO_2)]$ in CD_3CN.

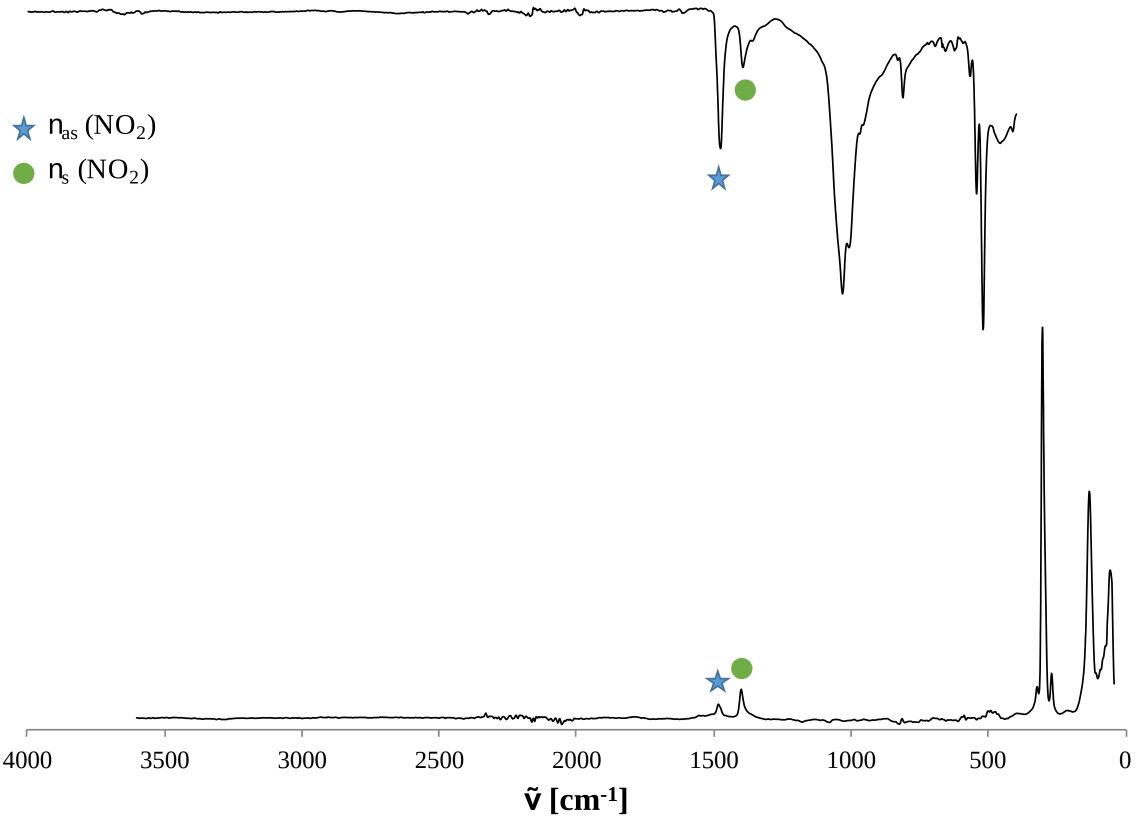

Figure S11: IR- (top) and Raman-spectra (bottom) of $Cs_2[B_{12}Cl_{11}(NO_2)]$.

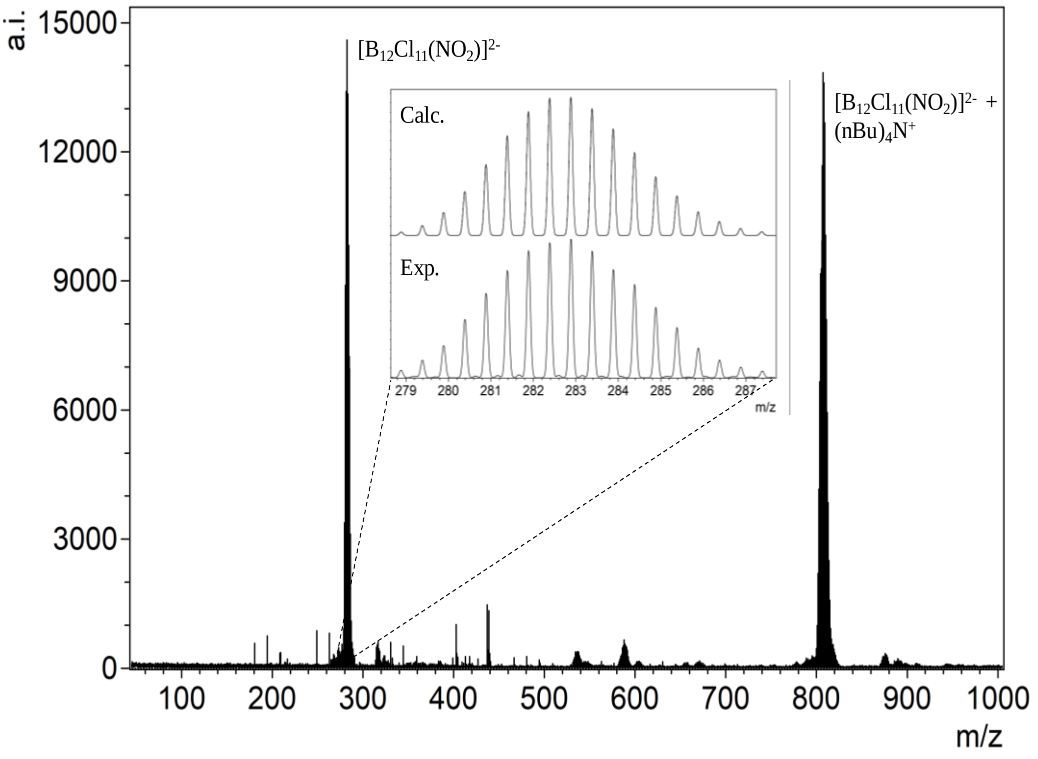

Figure S12: ESI mass spectrum (negative mode) of $[N(nBu)_4]_2[B_{12}Cl_{11}(NO_2)]$. The simulated mass spectrum is shown above.

S2.3 Synthesis and spectroscopic characterization of $M_2[B_{12}Br_{11}(NO_2)]$

$K[B_{12}Br_{11}(NH_3)]$ (0.85 g, 0.80 mmol, 1.0 eq.) was dissolved in 20 ml of hydrogen peroxide (35 %). The pH value was set to 9 by adding potassium hydroxide. The solution was heated to reflux (the oil bath temperature was set to 120 °C) under intense stirring (Caution: gas and heat evolution!) for 13 days. Three to four times a day addinitional 20 ml portions of hydrogen peroxide (35 %) were added and the pH value was adjusted again, if necessary. The reaction progress was monitored by ^{11}B NMR spectroscopy. After 13 days a total amount of 700 ml hydrogen peroxide was added. The mixture was continued stirring at elevated temperature until a test with potassium starch paper for remaining hydrogen peroxide was negative. Subsequently, the mixture was allowed to reach room temperature and the pH value was adjusted to 7 by adding hydrochloric acid and then filtered. A solution of $[N(nBu)_4]Br$ (0.54 g, 1.68 mmol, 2.1 eq.) in 5 ml of water was added dropwise to the filtrate under intense stirring to precipitate the product (Alternatively CsCl to give the cesium salt, $Et_3N{\cdot}HCl$ to give the $[Et_3NH]^+$ salt, or $[PPh_4]Cl$ to give the $[PPh_4]^+$ salt may be used.). The suspension was stirred for 20 minutes and then filtered. The solid residue was washed with water (3 × 10 ml), dissolved in acetonitrile, and filtered again. Evaporation of the solvent yielded $[(nBu)_4N]_2[B_{12}Br_{11}(NO_2)]$ (1.01 g, 0.66 mmol, 83 %) of as a white solid.

NMR data for $[N(nBu)_4]_2[B_{12}Br_{11}(NO_2)]$: ^{1}H and ^{13}C spectra are identical to those for $[N(nBu)_4]_2[B_{12}F_{11}(NO_2)]$. $^{11}B\{^{1}H\}$ NMR (128.38 MHz, CD_3CN, 298 K): δ = -10.0 (s, 1B), -10.9 (s, 1B), -12.3 (s, 5B), -14.2 (s, 5B).

IR (diamond ATR) of $Cs_2[B_{12}Br_{11}(NO_2)]$: $\tilde{\nu}$ = 3648 (w, ϖ_{as} (H_2O)), 3569 ϖ_s (H_2O), 1607 (w, δ (H_2O)), 1474 (s, ν_{as} (NO_2)), 1435 (w, sh), 1391 (m, ν_s (NO_2)), 1095 (m), 1048 (w), 998 (s, sh), 982 (vs, ν_{as} (B-Br)), 964 (s, sh), 806 (m, δ (NO_2)), 742 (w), 692 (w), 668 (w, sh), 596 (w), 464 (s, sh, δ (B-Cl)), 447 (vs, sh, δ (B-Cl)), 436 (vs, δ (B-Cl)).

Raman of $Cs_2[B_{12}Br_{11}(NO_2)]$: $\tilde{\nu}$ = 1477 (vw, ν_{as} (NO_2), 1392 (w, ν_s (NO_2)), 808 (vw), 278 (w), 251 (w), 195 (vs).

ESI-MS [m/z]: found = 527.1033 (calc. = 527.1027) ($[B_{12}Br_{11}(NO_2)]^{2-}$), 1297.4933 (calc. = 1297.4888) $[(N(nBu)_4)(B_{12}Br_{11}(NO_2))]^-$.

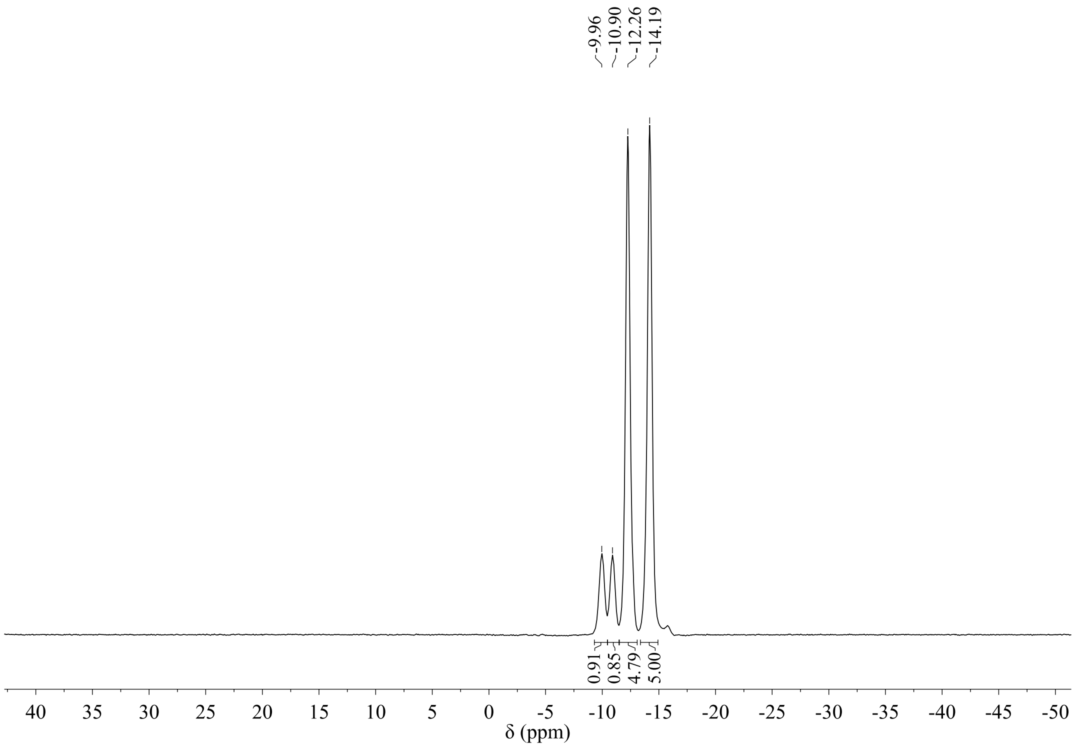

Figure S13: ^{11}B NMR spectrum (192.59 MHz, 298K) of $[N(nBu)_4]_2[B_{12}Br_{11}(NO_2)]$ in CD_3CN.

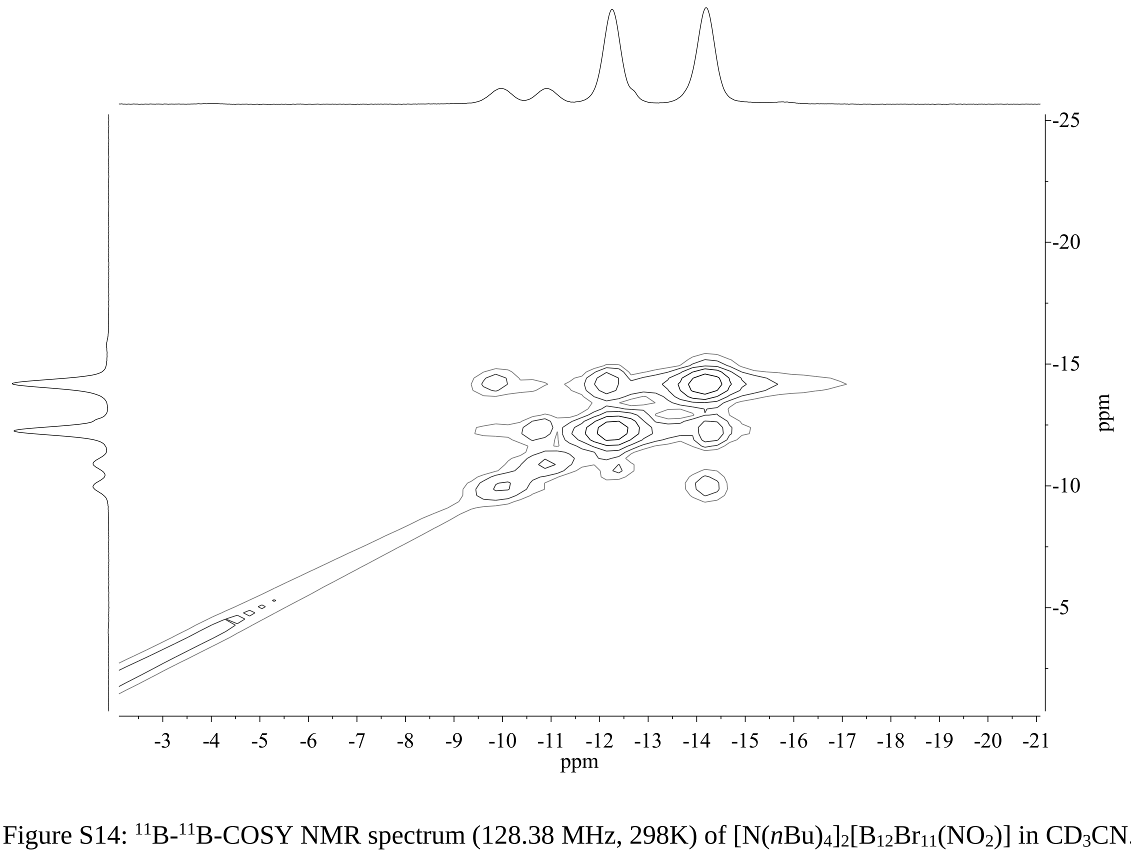

Figure S14: ^{11}B-^{11}B-COSY NMR spectrum (128.38 MHz, 298K) of $[N(nBu)_4]_2[B_{12}Br_{11}(NO_2)]$ in CD_3CN.

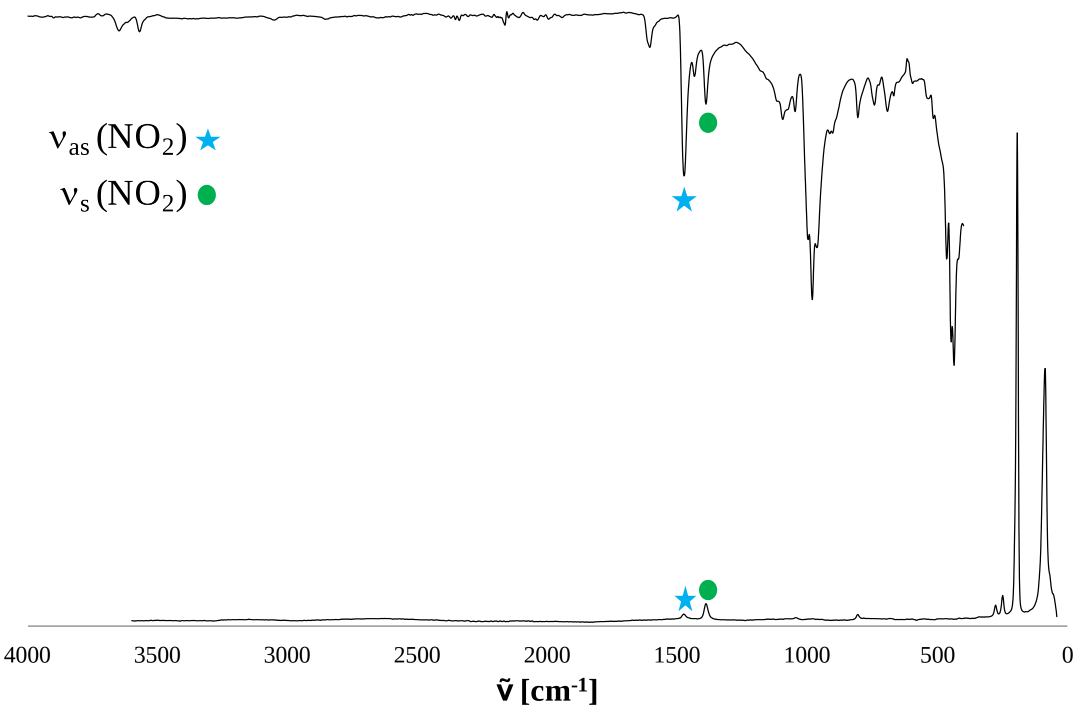

Figure S15: IR- (top) and Raman-spectra (bottom) of $Cs_2[B_{12}Br_{11}(NO_2)]$.

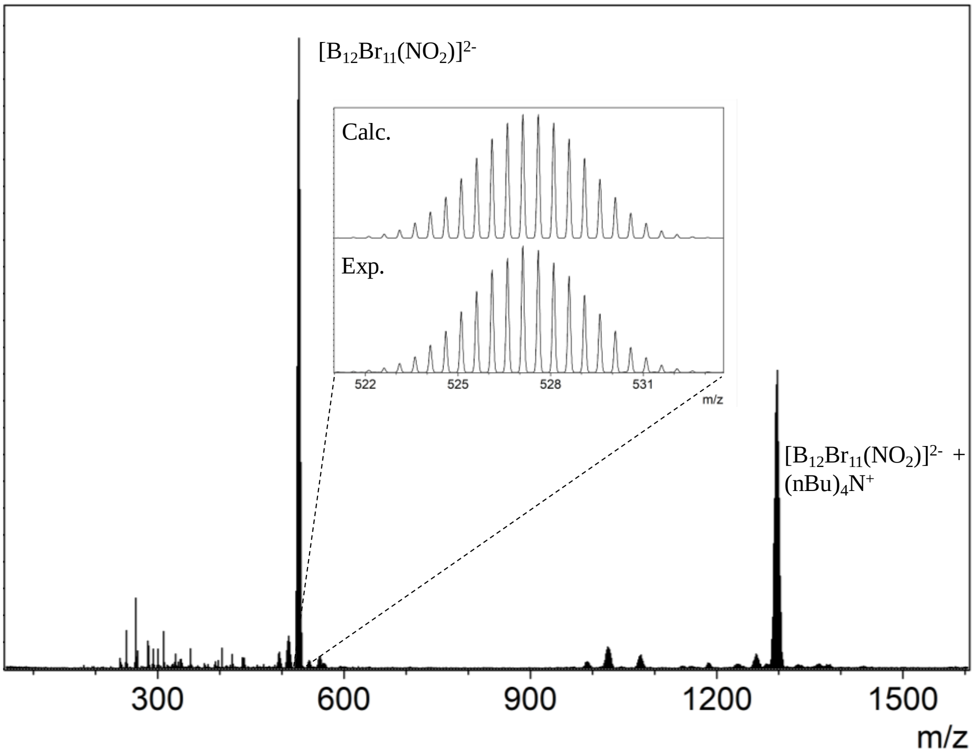

Figure S16: ESI-mass spectrum (negative mode) of $[N(nBu)_4]_2[B_{12}Cl_{11}(NO_2)]$. The simulated mass spectrum is shown above.

S2.4 Synthesis and spectroscopic characterization of $K_2[B_{12}I_{11}(NO_2)]$

$K[B_{12}I_{11}(NH_3)]$ (0.50 g, 0.32 mmol, 1.0 eq.) was dissolved in 20 ml of hydrogen peroxide (35 %). The pH value was set to 9 by adding potassium hydroxide. The solution was heated to reflux (the oil bath temperature was set to 120 °C) under intense stirring (**caution**: gas and heat evolution!) for eight days. Three to four times a day additional 20 ml portions of hydrogen peroxide (35 %) were added and the pH value was adjusted again, if necessary. The reaction process was monitored by ^{11}B-NMR spectroscopy. After eight days a total amount of 360 ml hydrogen peroxide was added. Subsequently, the reaction mixture was allowed to reach room temperature and filtered. The solvent was evaporated in vacuo. A yellow solid (0.34 g) was obtained containing $K_2[B_{12}I_{11}(NH_2)]$, $K_2[B_{12}I_{11}(NO_2)]$ and remaining KOH.

NMR data for the product mixture: $^{11}B\{^1H\}$ NMR (128.38 MHz, CD_3CN, 298 K): δ = -3.3 (s, $[B_{12}I_{11}(NH_2)]^{2-}$), -12.9 (s), -15.3 (s, $[B_{12}I_{11}(NO_2)]^{2-}$), -17.0 (s, $[B_{12}I_{11}(NH_2)]^{2-}$), -19.0 (s, $[B_{12}I_{11}(NO_2)]^{2-}$), -21.1 (s, $[B_{12}I_{11}(NH_2)]^{2-}$).

ESI-MS [m/z]: 771.0504 (calc. = 771.0441) $[B_{12}I_{11}(NH_2)]^{2-}$, 786.0404 (calc. = 786.0312) $[B_{12}I_{11}(NO_2)]^{2-}$, 1543.1504 (calc. = 1543.0948) $[B_{12}I_{11}(NH_3)]^{-}$.

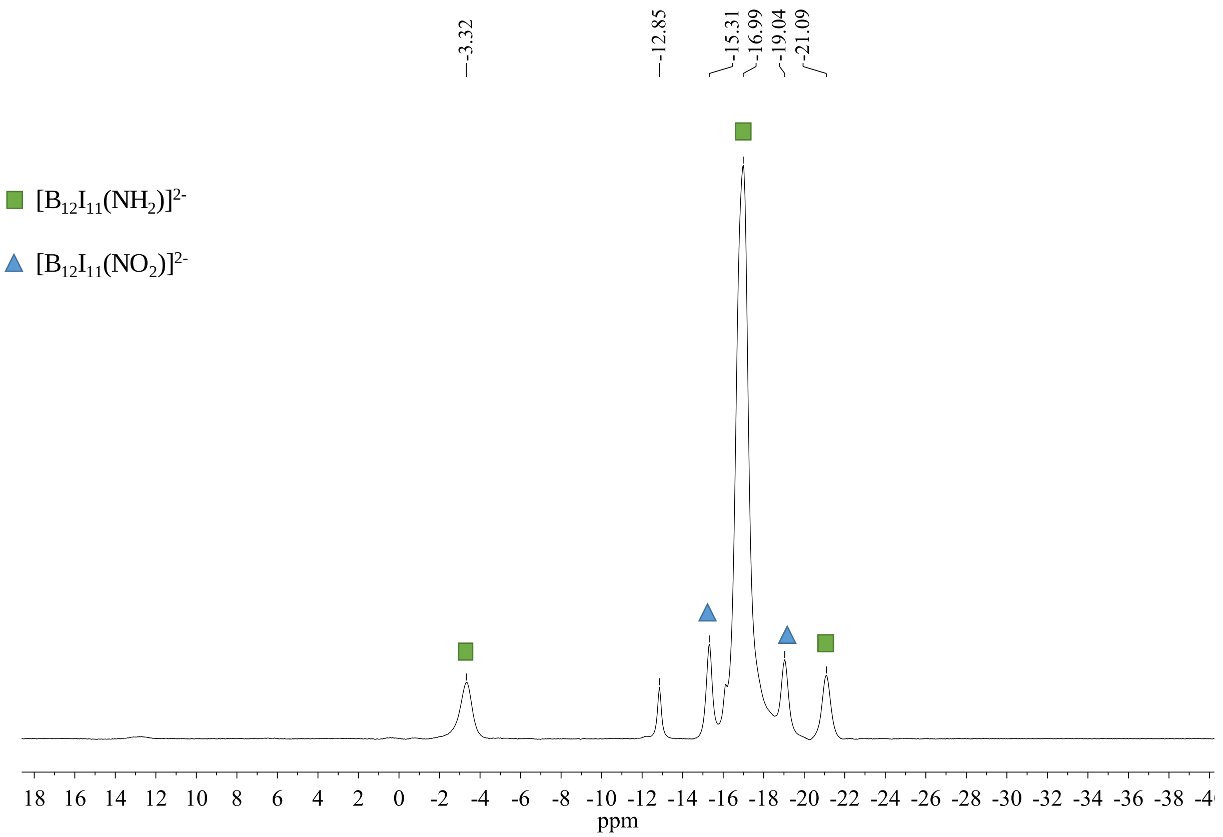

Figure S17: ^{11}B NMR spectrum (192.59 MHz, 298K) of the product mixture $K_2[B_{12}I_{11}(NO_2)]/K_2[B_{12}I_{11}(NH_2)]$ in CD_3CN.

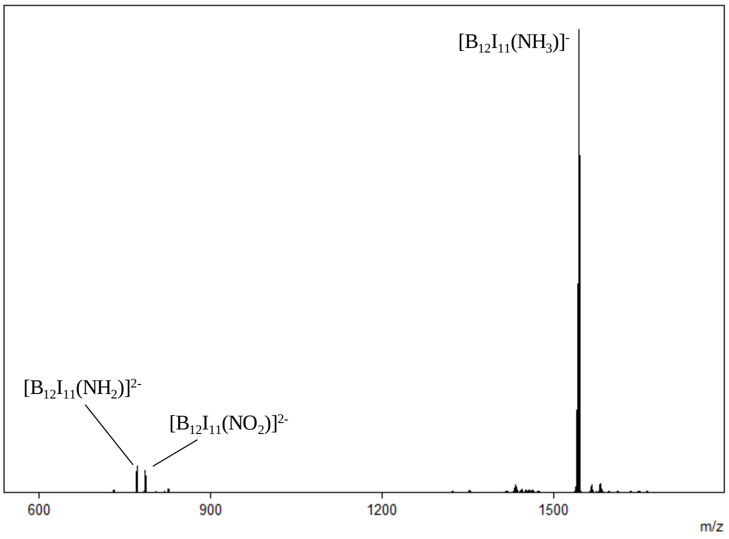

Figure S18: ESI-mass spectrum (negative mode) of the product mixture $K_2[B_{12}I_{11}(NO_2)]/K_2[B_{12}I_{11}(NH_2)]$.

S2.5 Monitoring the reaction progress of the oxidation over time

The reaction progress of the reaction of $[B_{12}Cl_{11}(NH_3)]^-$ with H_2O_2 was monitored over time by ^{11}B NMR spectroscopy. The NMR spectra of $[B_{12}Cl_{11}(NH_3)]^-$ change with the pH value of the solution due to protonation and deprotonation of the ammonio group.

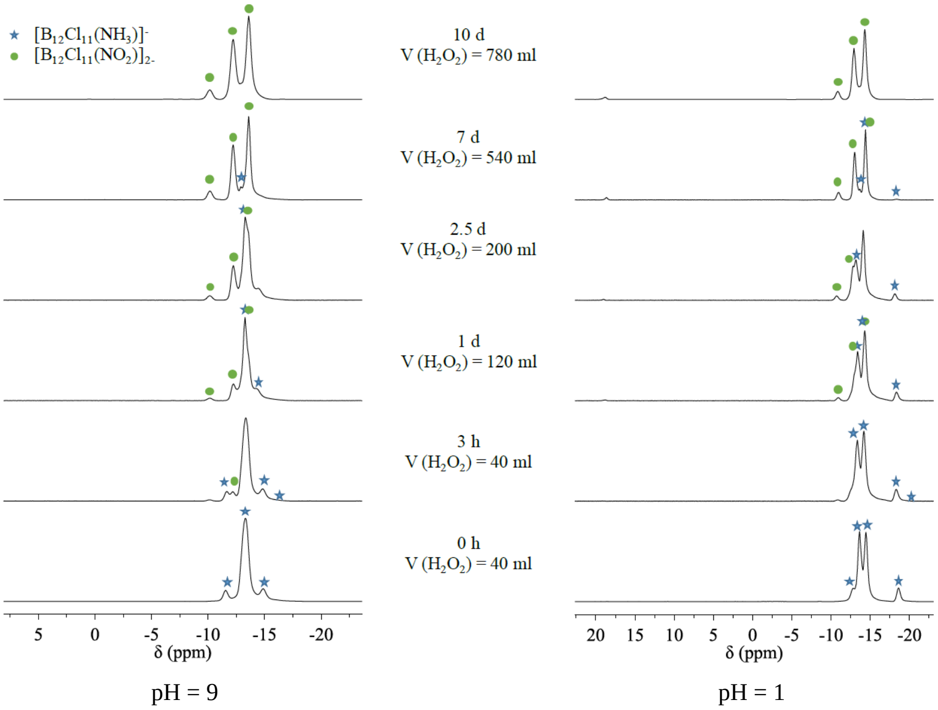

Figure S19: ^{11}B NMR (CD_3CN, 298 K) spectra of the oxidation of $[B_{12}Cl_{11}(NH_3)]^-$ depending on time and pH. Signals of $[B_{12}Cl_{11}(NH_3)]^-$ are marked with blue stars, signals of $[B_{12}Cl_{11}(NO_2)]^{2-}$ are marked with green circles.

S2.6 Comparison of the ^{11}B NMR spectra of $[B_{12}X_{11}(NO_2)]^{2-}$ (X = F, Cl, Br)

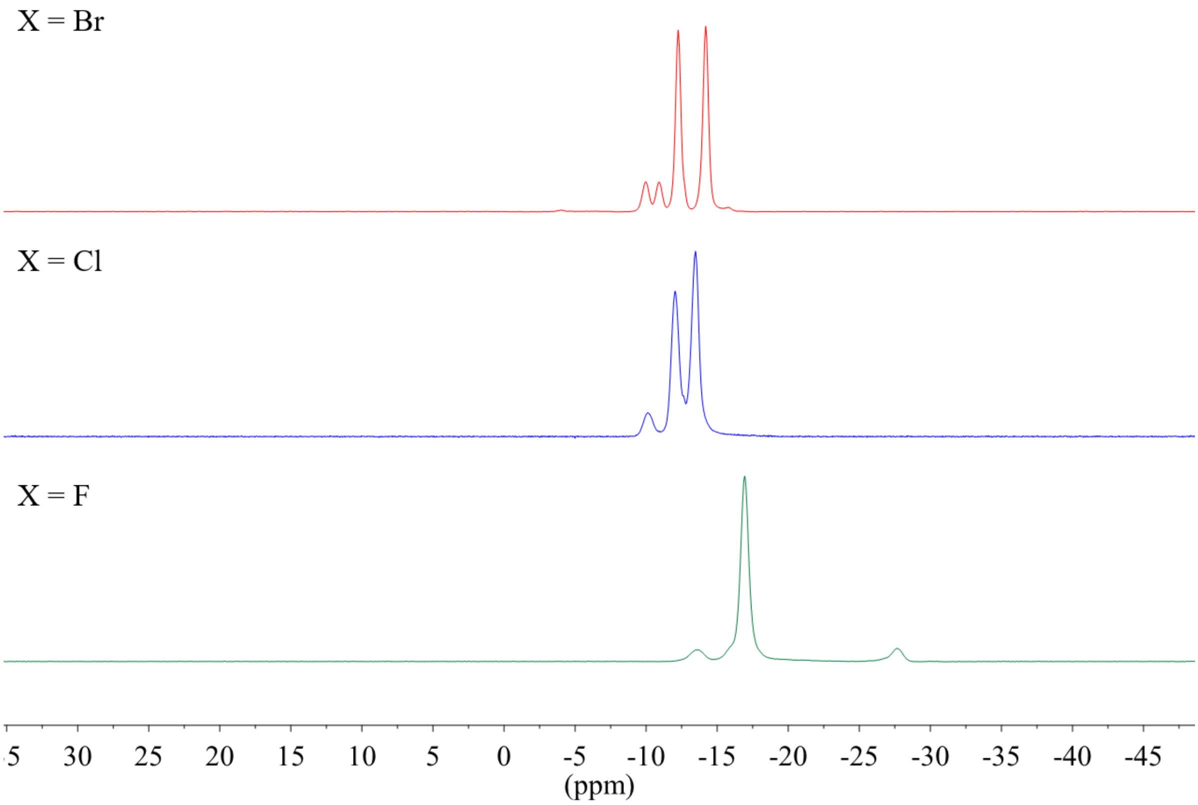

Figure S20: ^{11}B NMR spectra (128.38 MHz, CD_3CN, 298 K) of $[N(nBu)_4]_2[B_{12}X_{11}(NO_2)]$ (X = F, Cl, Br).

Table S1: ^{11}B NMR shifts of $[TBA]_2[B_{12}X_{11}(NO_2)]$ (X = F, Cl, Br) in CD_3CN.

**Anion**	^{11}B shift [ppm]	Ratio
$[B_{12}F_{11}(NO_2)]^{2-}$	-13.6 (B1), -16.9 (B2-11), -27.7 (B12)	1 : 10 : 1
$[B_{12}Cl_{11}(NO_2)]^{2-}$	-10.1, -12.0, -13.5	1 : 5 : 6
$[B_{12}Br_{11}(NO_2)]^{2-}$	-10.0, -10.9, -12.3, -14.2	1 : 1 : 5: 5

The ^{11}B NMR spectra for all three anions are significantly different. In the case of X = F the chemical shifts could be reproduced by quantum-chemical calculations and thus the resonances could be assigned to the different boron atoms.

S3 Cyclic voltammetry

Cyclic voltammetric measurements were carried out in liquid sulfur dioxide at temperatures below 243±3 K with a three electrode setup.

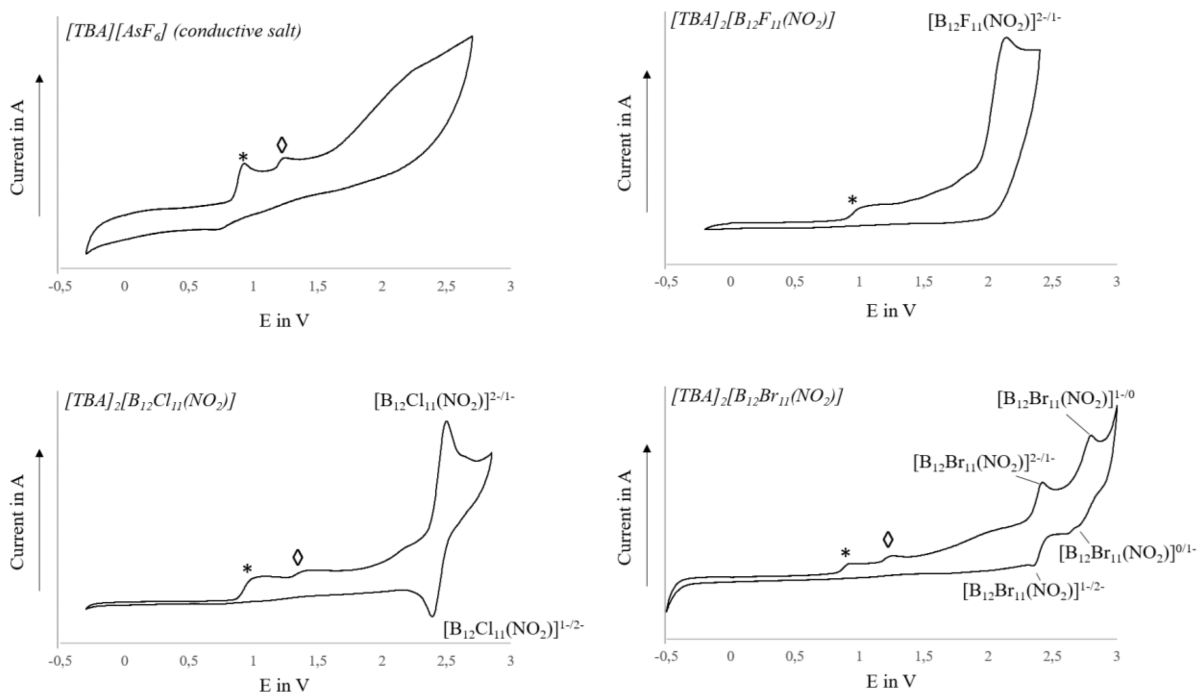

Figure S21: Cyclovoltammetric measurements (Sυ = 0.1 V s^{-1}) of $[N(nBu)_4]_2[B_{12}X_{11}(NO_2)]$ (X = F, Cl, Br) and $[N(nBu)_4][AsF_6]$ in liquid SO_2 at 243±3 K (0.1 M $[N(nBu)_4][AsF_6]$, Pt working electrode). The star (*) indicates the oxidation of $[N(nBu)_4]Br$ as an impurity in the conductive salt. The rhombus (◊) indicates the oxidation of an unkown impurity of the conductive salt.

Table S2: Summary of electrochemical potential data for $[B_{12}X_{11}(NO_2)]^{2-}$ in SO_2 and $[N(nBu)_4][AsF_6]$ as supporting electrolyte versus $Fc^{0/1}$.

Compound	E_p^{a1}[V]	E_p^{c1}[V]	ΔE^{a1-c1} [mV]	E_m[V]	E_p^{a2}[V]	ΔE^{2-1} [V]	T [°C]
$[N(nBu)_4]_2[B_{12}F_{11}(NO_2)]$	2.09	-	-	-	-	-	-30
$[N(nBu)_4]_2[B_{12}Cl_{11}(NO_2)]$	2.45	2.34	110	2.40	-	-	-30
$[N(nBu)_4]_2[B_{12}Br_{11}(NO_2)]$	2.39	2.31	80	2.35	2.72	0.33	-30

Table S3: Comparison of electrochemical potential data for $[B_{12}X_{11}(NO_2)]^{2-}$ in SO_2 with that for $[B_{12}X_{12}]^{2-}$. Values in brackets refer to the potentials of the second oxidation.

Anion	E^{a}_{p} [V]	Anion	E^{a}_{p} [V] [11b]	ΔE^{a}_{p} [V] [11b]
$[B_{12}F_{11}(NO_2)]^{2-}$	2.09	$[B_{12}F_{12}]^{2-}$	1.78	+0.31
$[B_{12}Cl_{11}(NO_2)]^{2-}$	2.45	$[B_{12}Cl_{12}]^{2-}$	2.15	+0.30
$[B_{12}Br_{11}(NO_2)]^{2-}$	2.39 (2.72)	$[B_{12}Br_{12}]^{2-}$	2.31 (2.67)	+0.08 (+0.05)
$[B_{12}I_{11}(NO_2)]^{2-}$	–	$[B_{12}I_{12}]^{2-}$	2.1	–

S4 Thermal NO Cleavage

Thermogravimetric and Differential scanning calorimetry were done in order to see a possible NO_2 or NO loss by thermal decomposition (see **Fehler! Verweisquelle konnte nicht gefunden werden.**). The curves were normalized to 100 % (mass). T_{Onset} and experimental mass loss were calculated automatically. At about 410 °C the $[N(nBu)_4]^+$ cations begins to decompose. Thus, this temperature range was not shown due to clarity.

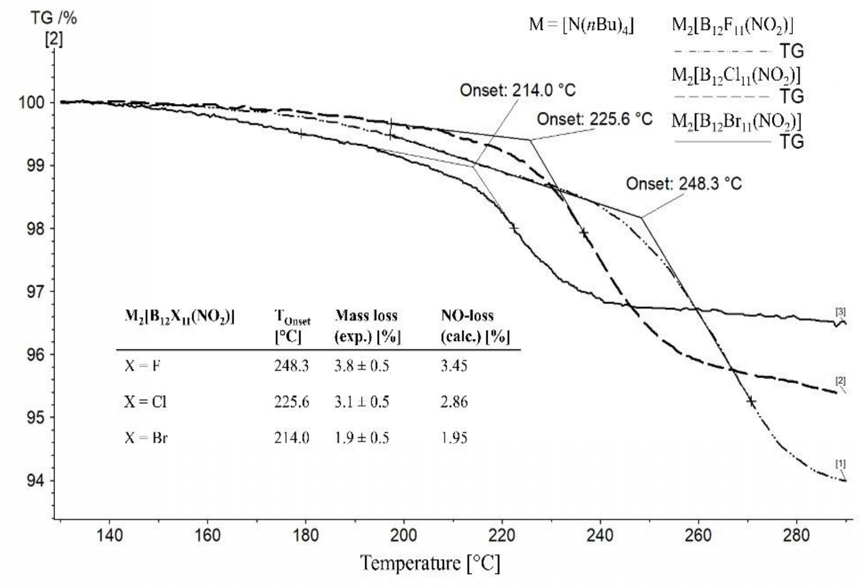

Figure S22: Thermogravimetric measurements of $[N(nBu)_4]_2[B_{12}X_{11}(NO_2)]$ (X = F, Cl, Br) with a heating rate of 5 K min^{-1}.

The measurements show that not NO_2, but NO does leave the molecules. The observed mass losses are in agreement with the expected cleavage of nitric oxide.

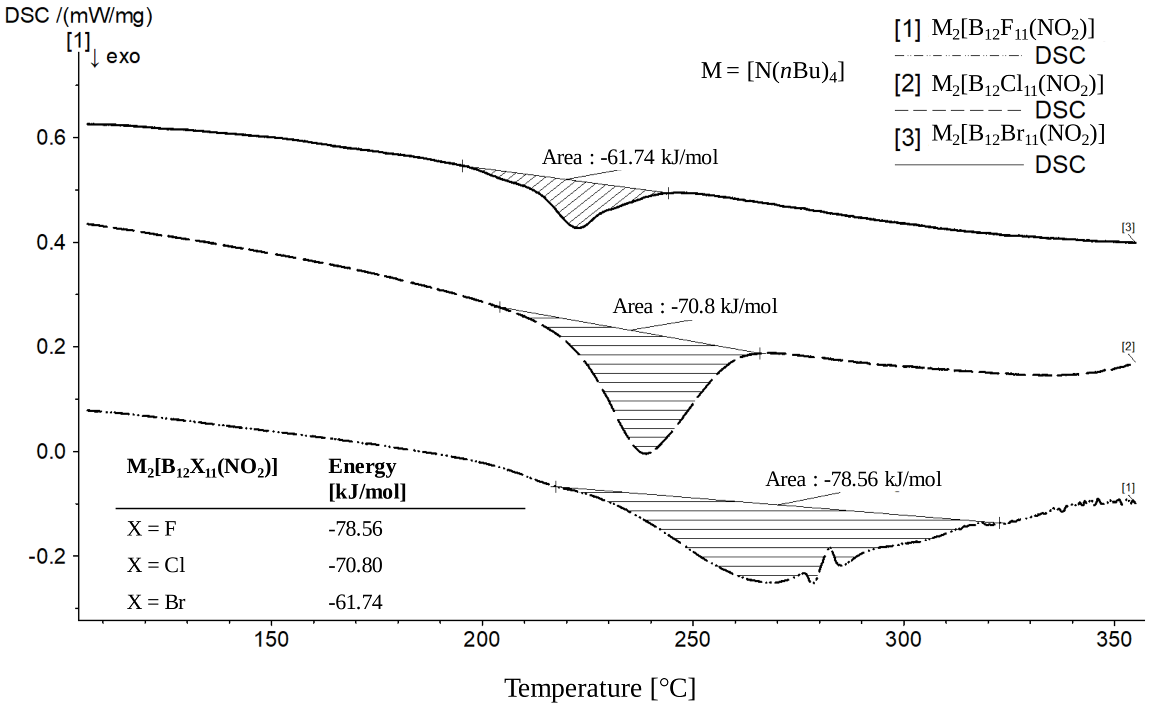

Figure S23: Differential scanning calorimetry measurements of $[N(nBu)_4]_2[B_{12}X_{11}(NO_2)]$ (X = F, Cl, Br) with a heating rate of 5 K/min. In the case of $[N(nBu)_4]_2[B_{12}F_{11}(NO_2)]$ the spike at about 280 °C is reproducible and could not be avoid.

Table S4: Summary of thermogravimetric and differential scanning calorimetric data for $[B_{12}X_{11}(NO_2)]^{2-}$.

Compound	T_{Onset} [°C]	Mass loss (exp.) [%]	NO-loss (calc.) [%]	Energy [kJ mol^{-1}]
$[N(nBu)_4]_2[B_{12}F_{11}(NO_2)]$	248.3	3.8 ± 0.5	3.45	-78.56
$[N(nBu)_4]_2[B_{12}Cl_{11}(NO_2)]$	225.6	3.1 ± 0.5	2.86	-70.80
$[N(nBu)_4]_2[B_{12}Br_{11}(NO_2)]$	214.0	1.9 ± 0.5	1.95	-61.74

To ensure, that the residue contains remaining $[B_{12}X_{11}OH]^{2-}$ another reaction was done using the $Cs_2[B_{12}F_{11}(NO_2)]$. After 40 minutes of heating 20 mg of the sample at 300 °C in an open vessel the product was analyzed by infrared, nuclear magnetic resonance spectroscopy and mass spectrometry (see **Fehler! Verweisquelle konnte nicht gefunden werden.**–26). The spectra show the absence of remaining $[B_{12}F_{11}(NO_2)]^{2-}$ and indeed the presence of $[B_{12}F_{11}OH]^{2-}$.

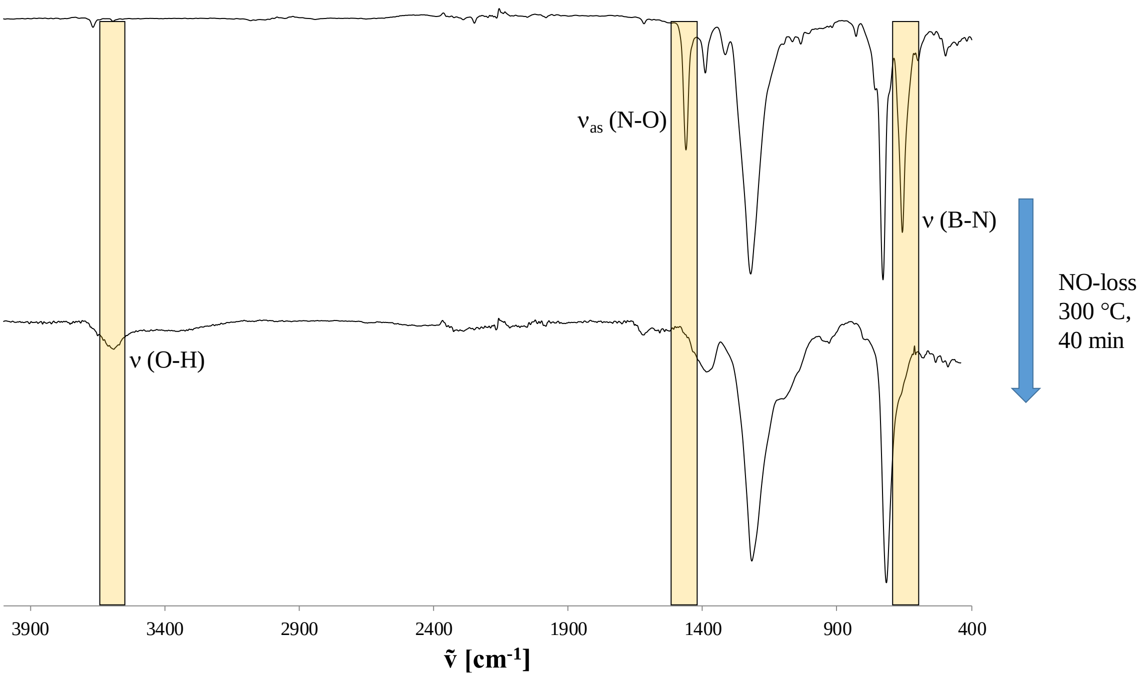

Figure S24: Infrared spectra of $Cs_2[B_{12}F_{11}(NO_2)]$ (top) and $Cs_2[B_{12}F_{11}OH]$ (bottom).

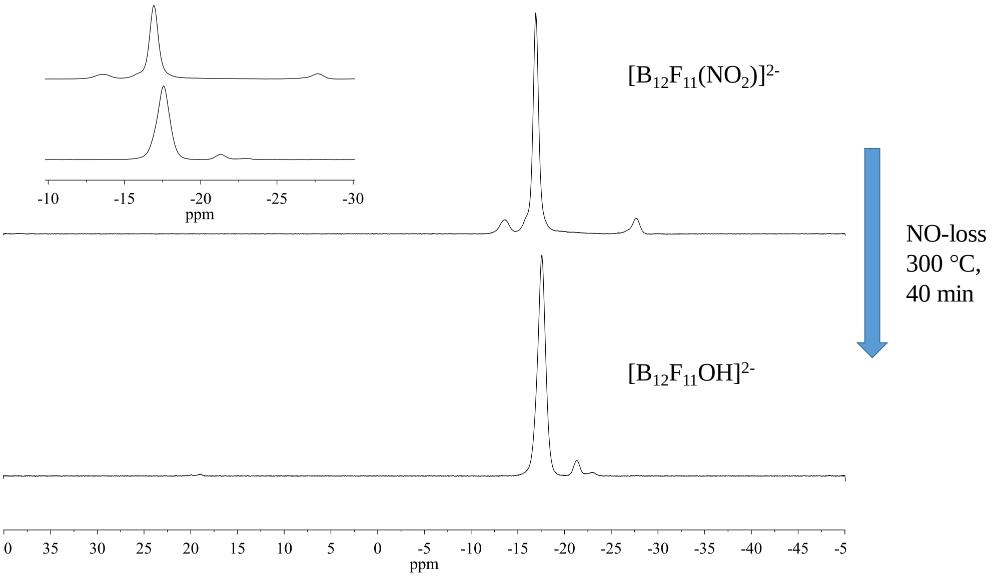

Figure S25: ^{11}B NMR (128.38 MHz, CD_3CN, 298 K) spectra of $Cs_2[B_{12}F_{11}(NO_2)]$ (top) and $Cs_2[B_{12}F_{11}OH]$ (bottom).

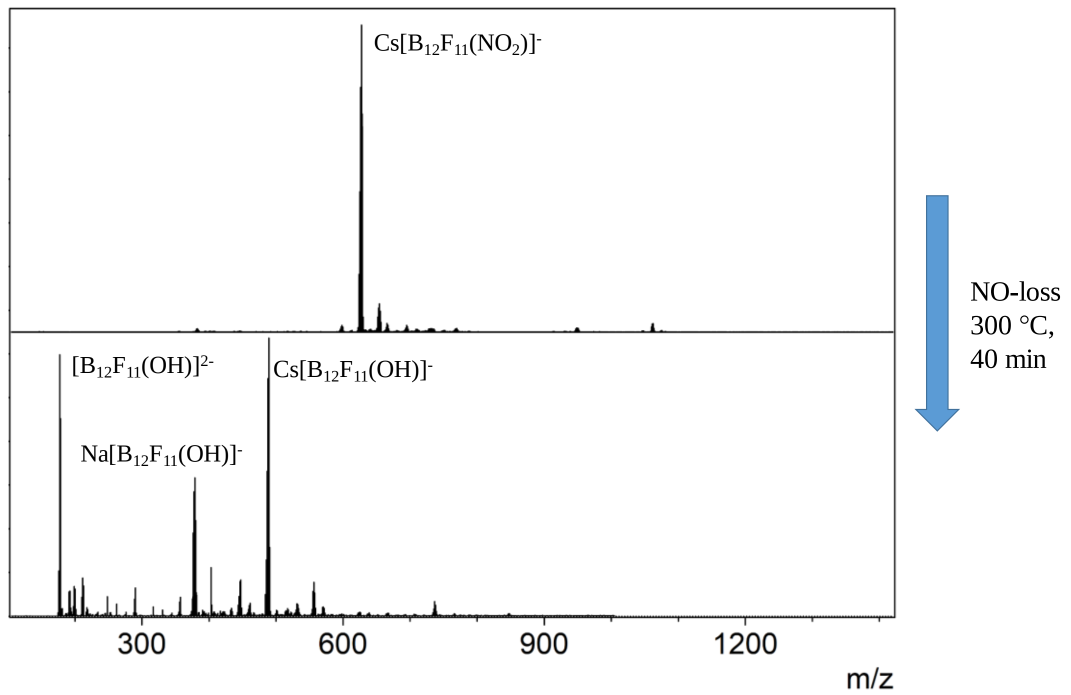

Figure S26: ESI-mass spectrum (negative mode) of $Cs_2[B_{12}F_{11}(NO_2)]$ (top) and $Cs_2[B_{12}F_{11}OH]$ (bottom).

S5 Reduction of the nitro group by nascent hydrogen

In order to reduce the nitro group in $[B_{12}X_{11}(NO_2)]^{2-}$ chemically $[TBA]_2[B_{12}Cl_{11}(NO_2)]$ was dissolved in an acetonitrile/water mixture. An excess of zinc chips was added and concentrated hydrochloride acid was added dropwise at room temperature to produce nascent hydrogen. After 30 minutes of stirring only a very small conversion was observed. Therefore, additional zinc and acid was added and the temperature was increased to 90 °C. After one hour of stirring the reaction mixture was allowed to cool to room temperature overnight. The mixture was then analyzed by mass spectrometry (see **Fehler! Verweisquelle konnte nicht gefunden werden.**27).

Besides several zinc chloride anions of the general formula $[Zn_xCl_{2x+1}]^-$ (x = 1-5) three signals that corresponds to *closo*-dodecaborates were observed. The reaction was not complete, what is evident by the presence of remaining $[B_{12}Cl_{11}(NO_2)]$. Nevertheless, the desired product ammonio group was formed as shown by the presence of $[B_{12}Cl_{11}(NH_3)]^-$. The strongest signal corresponds to the anion. In which the ammonio group is partially hydroxylated ($[B_{12}Cl_{11}(NH_2OH)]^-$).

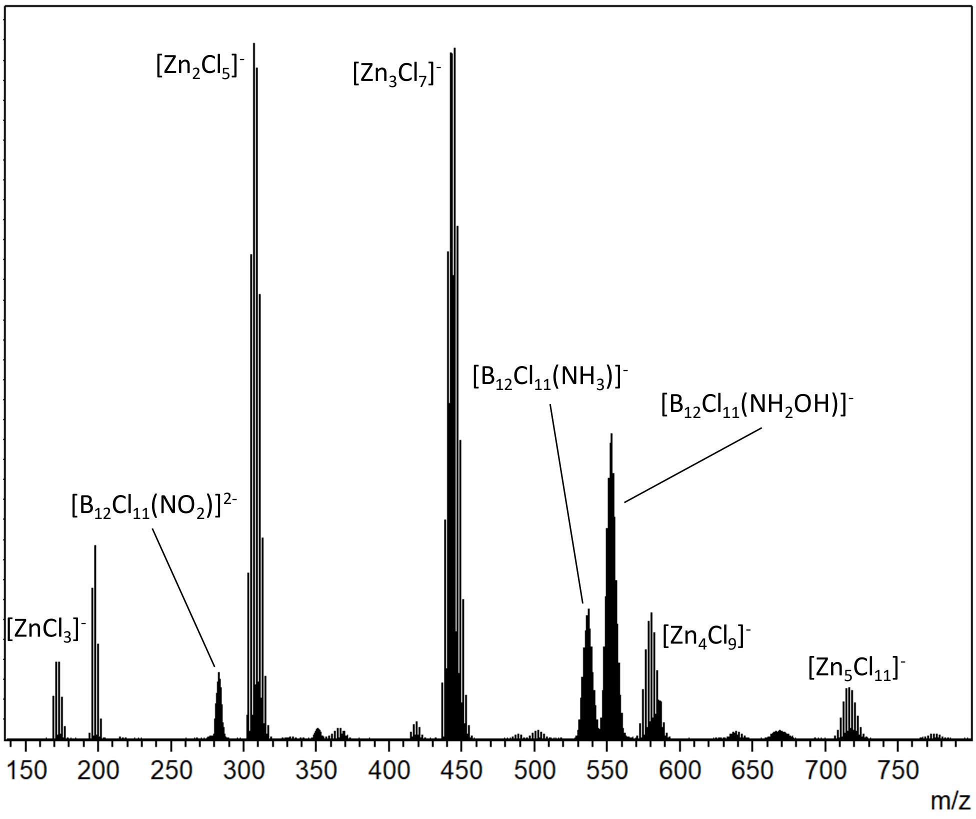

Figure S27: Mass spectrum (ESI, neg. mode) of the reaction mixture.

S6 Crystal structures

Table S5: Crystallographic data, part 1.

	$Cs_2[B_{12}F_{11}(NO_2)]$ · 2 CH_3CN	$[N(nBu)_4]_2$ $[B_{12}Br_{11}(NO_2)]$ · CH_3CN	$Cs_2[B_{12}Br_{11}(NO_2)]$ · 2 CH_3CN	$[PPh_4]_2[B_{12}Br_{11}(NO_2)]$	$Cs_2[B_{12}Cl_{11}(NO_2)]$ · 2 CH_3CN
Formula	$C_4H_6B_{12}Cs_2F_{11}N_3O_2$	$C_{34}H_{75}B_{12}Br_{11}N_4O_2$	$C_4H_6B_{12}Br_{10.98}Cs_2N_3O_2$	$C_{48}H_{40}B_{12}Br_{11}NO_2P_2$	$C_4H_6B_{12}Cl_{11}Cs_2N_3O_2$
CCDC Nr	2009135	2009136	2009137	2009138	2009139
M	732.66	1580.71	1401.07	1733.48	913.61
Temperature / K	150(1)	150(1)	150(1)	150(1)	150(1)
Crystal system	triclinic	triclinic	monoclinic	triclinic	monoclinic
Space group	$P\bar{1}$	$P\bar{1}$	*I*2/m	$P\bar{1}$	*I*2/m
a/pm	826.96(4)	1159.78(5)	1022.86(3)	1159.60(5)	988.36(3)
b/pm	891.86(5)	1257.85(5)	1198.24(3)	1178.46(5)	1162.57(4)
c/pm	1599.60(8)	2131.85(7)	1256.70(4)	1286.45(6)	1208.69(4)
α /°	87.492(4)	74.314(3)	90	70.881(4)	90
β /°	88.588(4)	81.473(3)	90.102(3)	72.480(4)	98.974(3)
γ /°	62.586(5)	77.312(3)	90	66.648(4)	90
U/nm^3	1.04626(10)	2.9080(2)	1.52086(8)	1.49496(13)	1.37183(8)
Z	2	2	2	1	2
$\mu(Mo\text{-}K_\alpha)/mm^{-1}$	3.590	7.611	16.834	7.462	3.743
No. of data collected	9331	25895	6227	15389	3394
No. of unique data	4786	11111	1724	6922	1668
R_{int}	0.0166	0.0334	0.0292	0.0274	0.0237
R_1, wR_2 (I>2σ(I))[a]	0.0204, 0.0484	0.0398, 0.0976	0.0280, 0.0693	0.0301, 0.0671	0.0241, 0.0544
R_1, wR_2 (all data)	0.0233, 0.0496	0.0542, 0.1046	0.0318, 0.0708	0.0449, 0.0719	0.0286, 0.0569

[a] $R_1 = \sum\left|\,|F_o| - |F_c|\,\right| / \sum|F_o|$, $wR_2 = (\sum[w(F_o^2-F_c^2)^2]/\sum[wF_o^4])^{1/2}$

Table S6: Crystallographic data, part 2.

	$[HNEt_3]_2[B_{12}Cl_{11}(NO_2)]$ · 2 CH_3CN	$Cs_2[B_{12}Cl_{11}(NO_2)]$ · 4 $(CH_3)_2SO$	$[PPh_4]_2[B_{12}Cl_{11}(NO_2)]$	$[Ba(CH_3CN)_8(H_2O)]$ $[B_{12}Cl_{11}(NO_2)]$ · CH_3CN · OEt_2
Formula	$C_{16}H_{38}B_{12}Cl_{11}N_5O_2$	$C_8H_{24}B_{12}Cl_{11}Cs_2NO_6S_4$	$C_{48}H_{40}B_{12}Cl_{11}NO_2P_2$	$C_{22}H_{39}B_{12}BaCl_{11}N_{10}O_4$
CCDC-Nr.	2009140	2009141	2009142	2009143
M	852.18	1144.01	1244.42	1164.64
Temperature / K	150(1)	150(1)	149.95(10)	150(1)
Crystal system	monoclinic	triclinic	monoclinic	monoclinic
Space group	$I2/m$	$P\bar{1}$	$P2_1/n$	$C2/c$
a/pm	1203.87(5)	989.88(5)	1502.18(9)	2637.33(7)
b/pm	1190.10(5)	1036.79(6)	1018.16(6)	1512.44(3)
c/pm	1474.56(7)	1045.69(6)	1829.11(11)	2751.96(8)
α /°	90	73.380(5)	90	90
β /°	11.9010(5)	73.707(5)	100.718(6)	107.749(3)
γ /°	90	89.224(5)	90	90
U/nm^3	2.06945(16)	0.98446(10)	2.7487(3)	10.4545(5)
Z	2	1	2	8
μ(Mo-K$_\alpha$)/mm^{-1}	0.764	2.839	0.656	1.361
No. of data collected	8611	16402	16931	28319
No. of unique data	2584	4786	6518	12107
R_{int}	0.0236	0.0405	0.0451	0.0273
R_1, wR_2 (I>2σ(I))[a]	0.0481, 0.1305	0.0319, 0.0616	0.0460, 0.0850	0.0438, 0.1027
R_1, wR_2 (all data)	0.0573, 0.1389	0.0461, 0.0669	0.0794, 0.0972	0.0602, 0.1123

[a] $R_1 = \sum \lvert\lvert F_o \rvert - \lvert F_c \rvert\rvert / \sum \lvert F_o \rvert$, $wR_2 = (\sum[w(F_o^2-F_c^2)^2]/\sum[wF_o^4])^{1/2}$

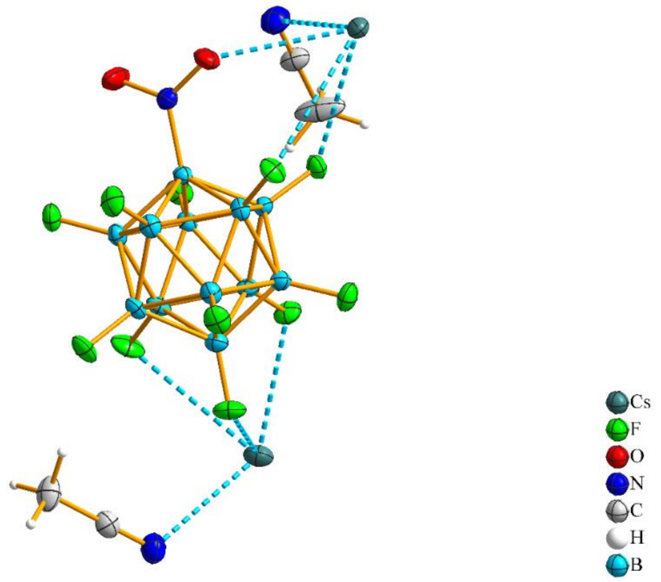

Figure S28: Part of the crystal structure of $Cs_2[B_{12}F_{11}(NO_2)]\cdot 2\ CH_3CN$. Ellipsoids are drawn at 50% probability and hydrogen atoms are drawn with arbitrary radii.

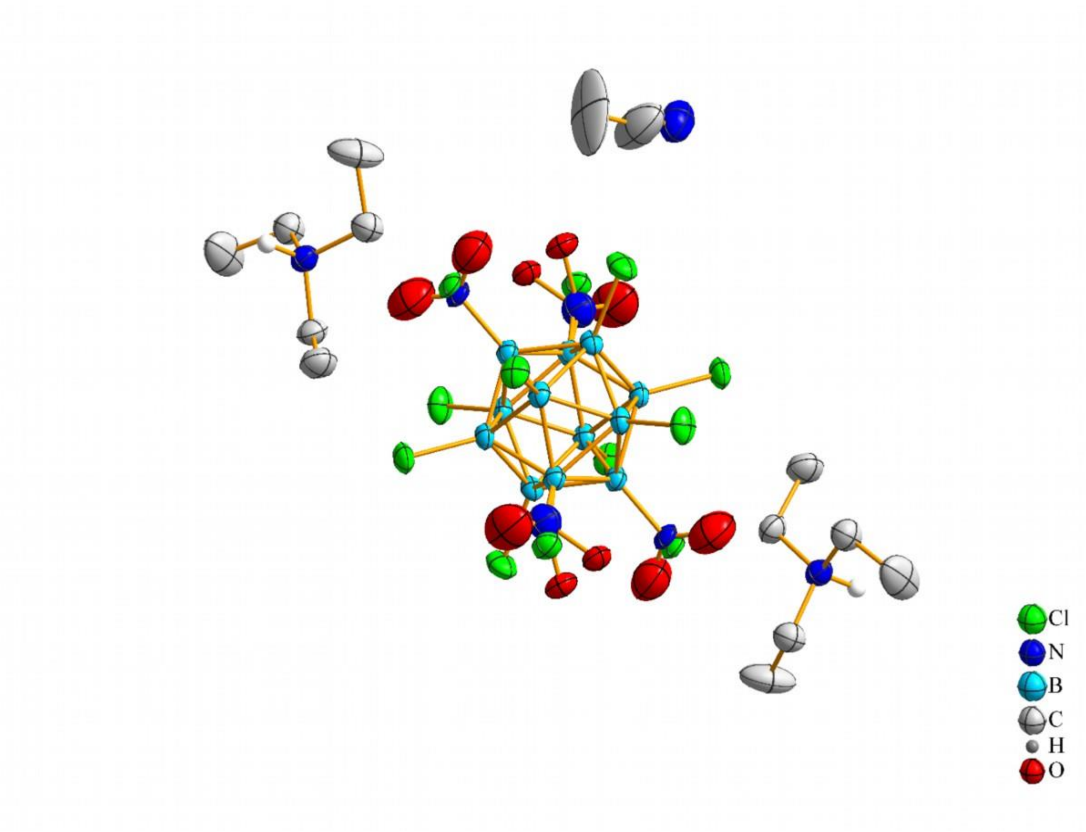

Figure S29: Part of the crystal structure of $[HNEt_3][B_{12}Cl_{11}(NO_2)]\cdot 2\ CH_3CN$. Ellipsoids are drawn at 50% probability and hydrogen atoms are drawn with arbitrary radii. For clarity not all hydrogen atoms are shown. The

nitro group is disordered over four positions. The acetonitrile solvent molecule and the $[HNEt_3]^+$ cations are disordered as well and only the major components are shown.

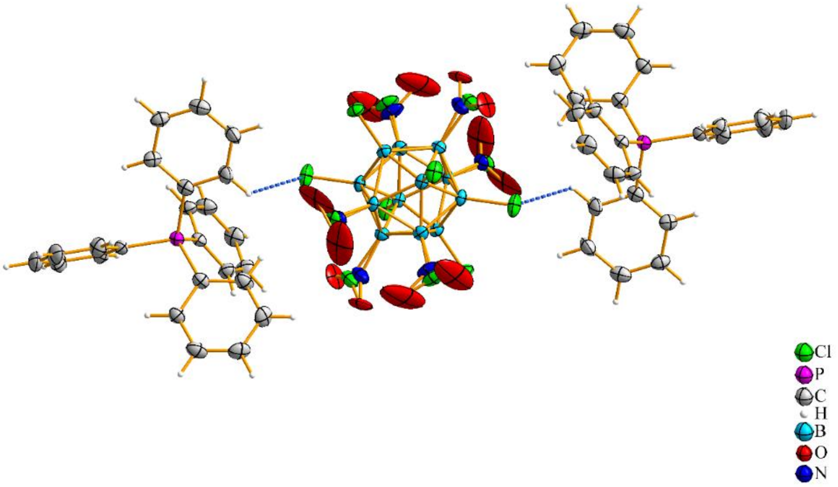

Figure S30: Part of the crystal structure of $[PPh_4]_2[B_{12}Cl_{11}(NO_2)]NO_3$. Ellipsoids are drawn at 50% probability and hydrogen atoms are drawn with arbitrary radii. The nitro group is disordered over six positions.

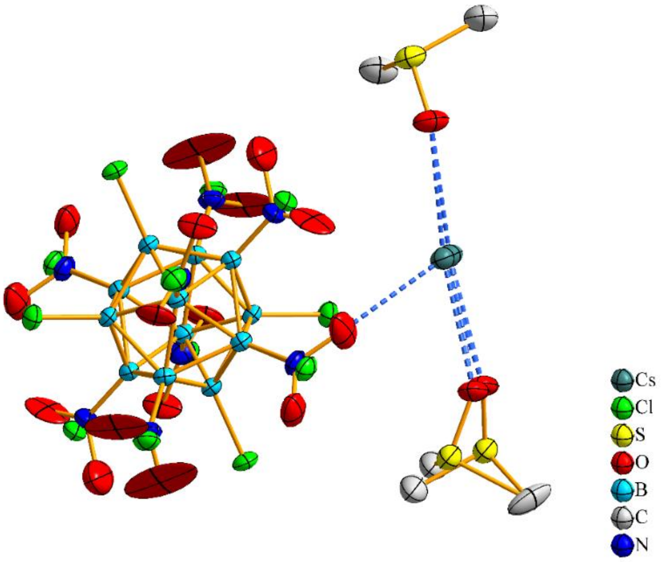

Figure S31: Part of the crystal structure of $Cs_2[B_{12}Cl_{11}(NO_2)]\cdot 4\,(CH_3)_2SO$. Ellipsoids are drawn at 50% probability and hydrogen atoms were omitted for clarity. The nitro group is disordered over eight positions.

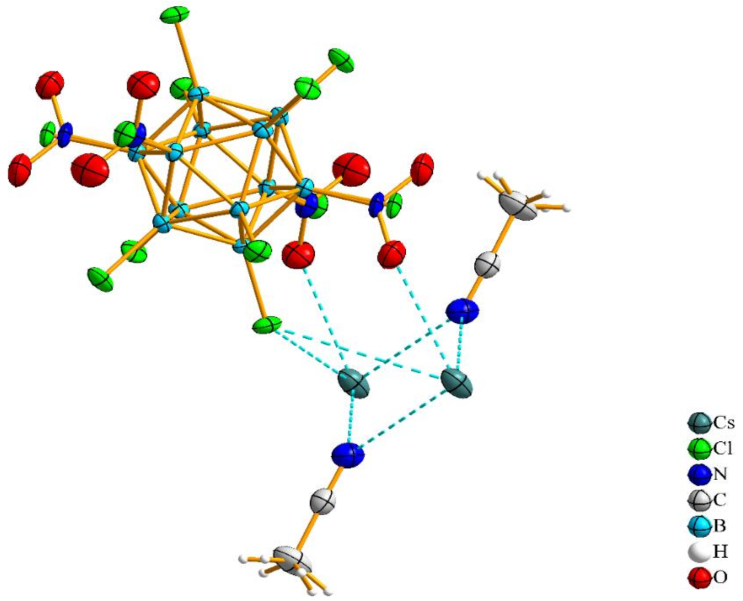

Figure S32: Part of the crystal structure of $Cs_2[B_{12}Cl_{11}(NO_2)]\cdot 2\ CH_3CN$. Ellipsoids are drawn at 50% probability and hydrogen atoms are drawn with arbitrary radii. The nitro group is disordered over four positions.

.

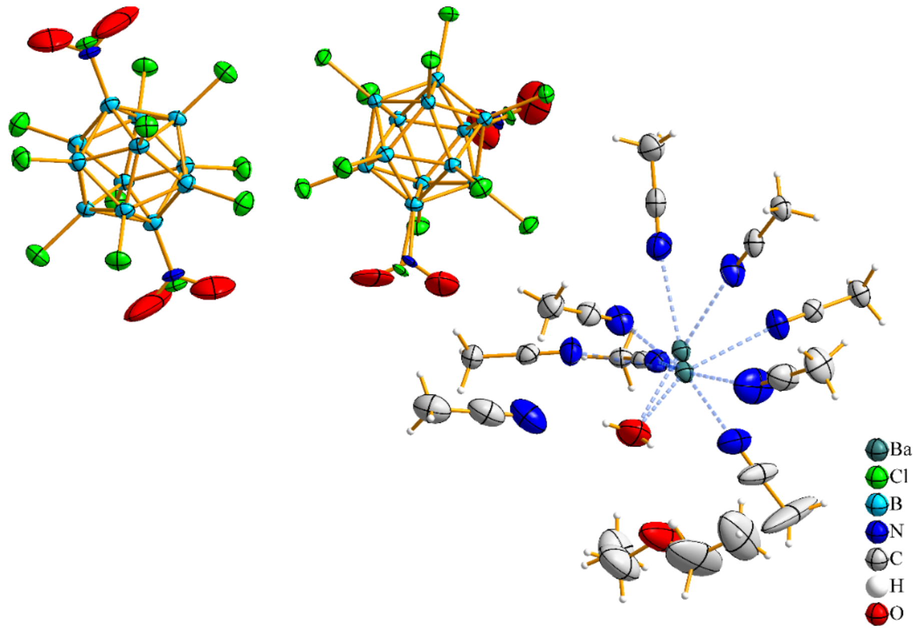

Figure S33: Part of the crystal structure of $[Ba(CH_3CN)_8(H_2O)][B_{12}Cl_{11}(NO_2)]\cdot\ CH_3CN\cdot\ OEt_2$. Ellipsoids are drawn at 50% probability and hydrogen atoms are drawn with arbitrary radii. The structure contains two independent clusters. In both clusters the nitro group is disordered over two positions. In one cluster the disordered nitro groups are in 1,12 position, while they are in 1,7 position in the second cluster.

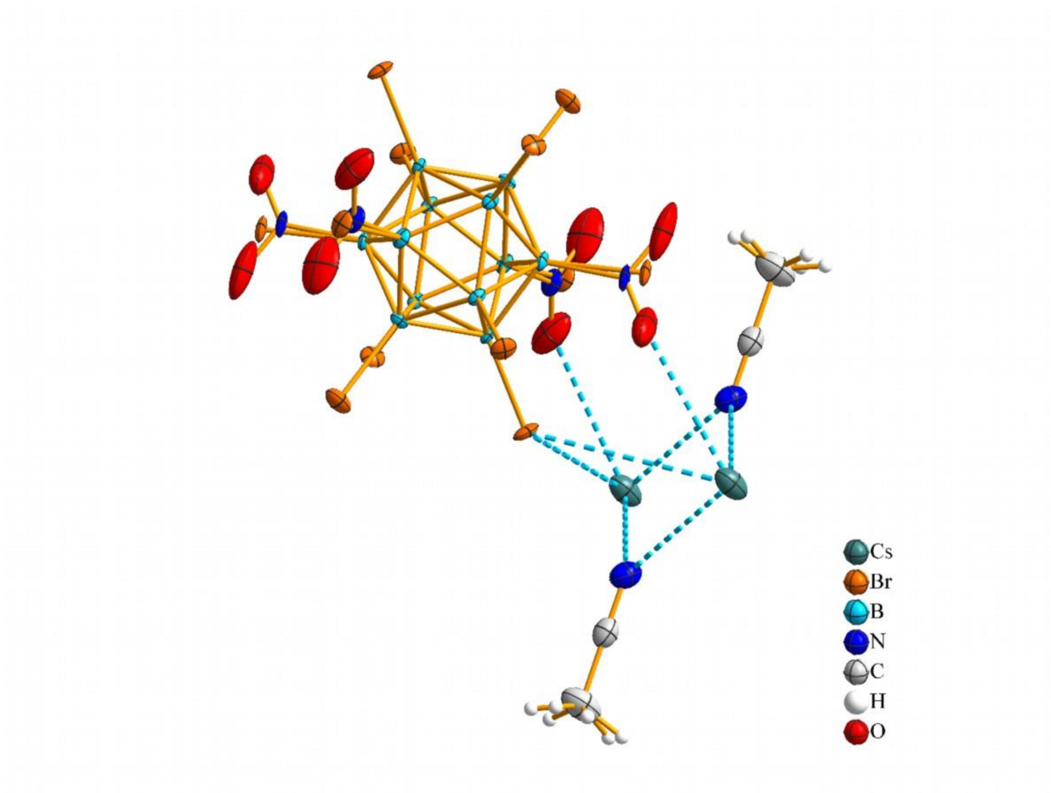

Figure 34: Part of the crystal structure of $Cs_2[B_{12}Br_{11}(NO_2)]\cdot 2\ CH_3CN$. Ellipsoids are drawn at 50% probability and hydrogen atoms are drawn with arbitrary radii.

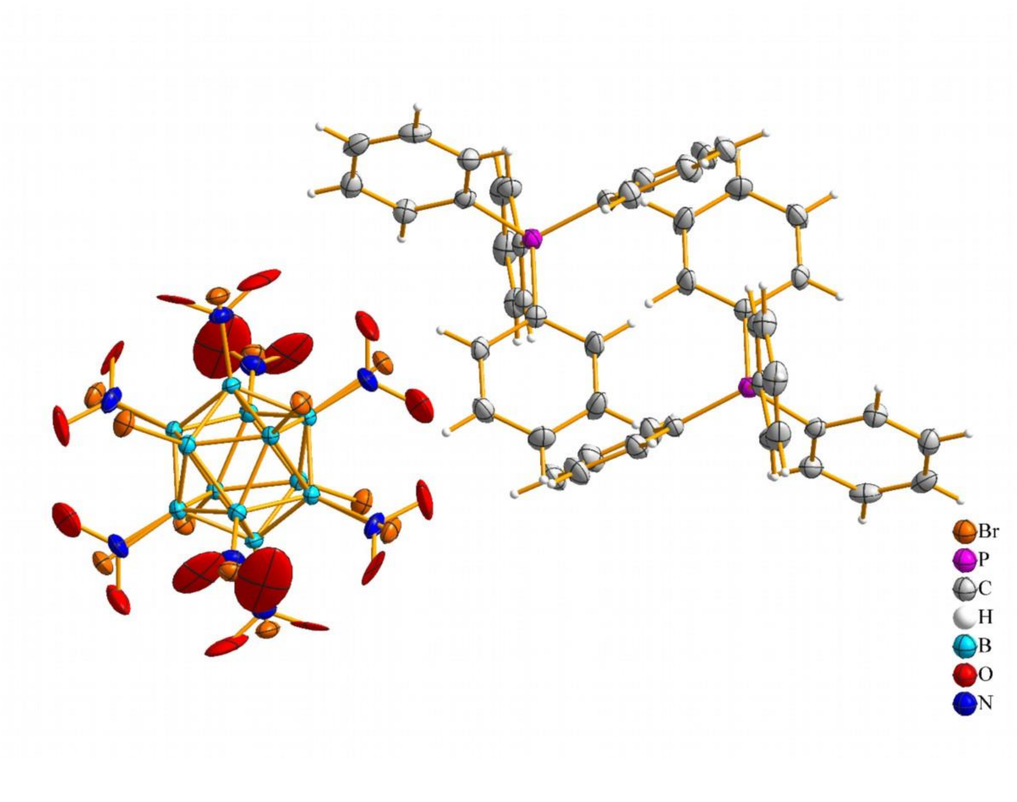

Figure S35: Part of the crystal structure of $[PPh_4]_2[B_{12}Br_{11}(NO_2)]$. Ellipsoids are drawn at 50% probability and hydrogen atoms are drawn with arbitrary radii. The nitro group is disordered over eight positions.

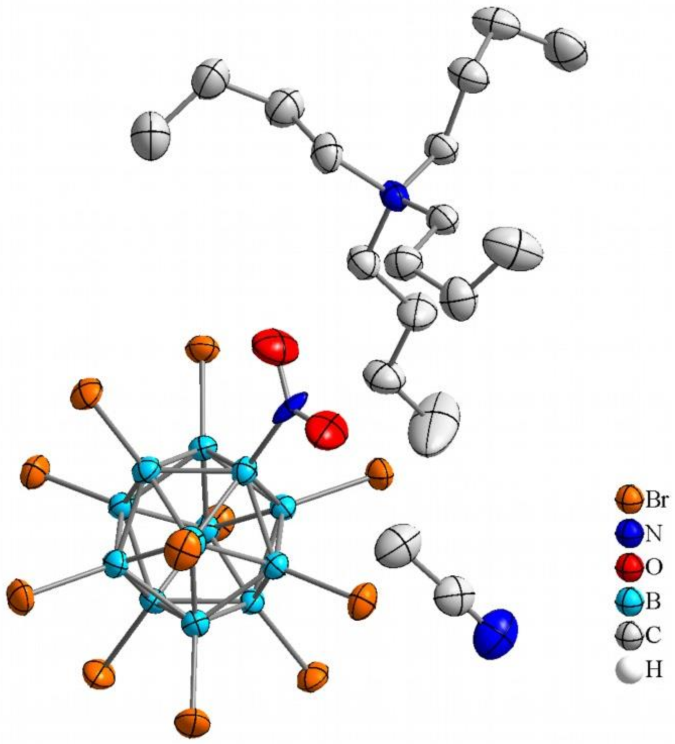

Figure S36: Part of the crystal structure of $[N(nBu)_4]_2[B_{12}Br_{11}(NO_2)]\cdot CH_3CN$. Ellipsoids are drawn at 50% probability and hydrogen atoms were omitted for clarity. The butyl groups of one $[N(nBu)_4]^+$ cation are disordered over two positions. Only the major component is visualized.

S7 Photoelectron spectroscopy

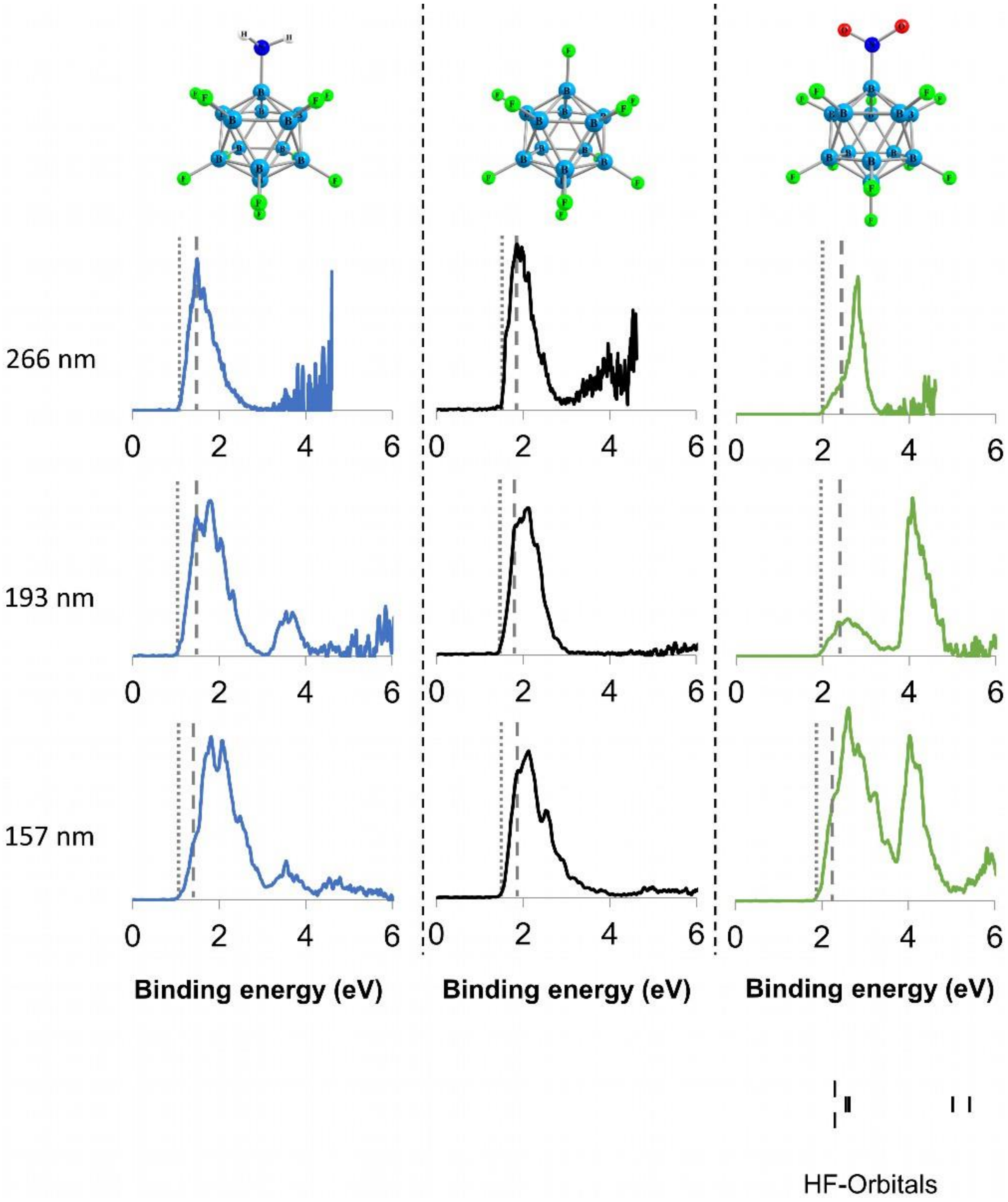

Figure S37: Photoelectron spectra of $[B_{12}F_{11}X]^{2-}$ (X = NH_2, F, NO_2) measured at different wavelengths. Dashed lines indicate vertical detachment energies (VDE) and dotted lines adiabatic detachment energies (ADE). Denotation of the density of states (DOS) predicted by Hartee-Fock calculations is shown below. Note that the highest occupied molecular orbital (HOMO) energy was shifted to match the experimental VDE.

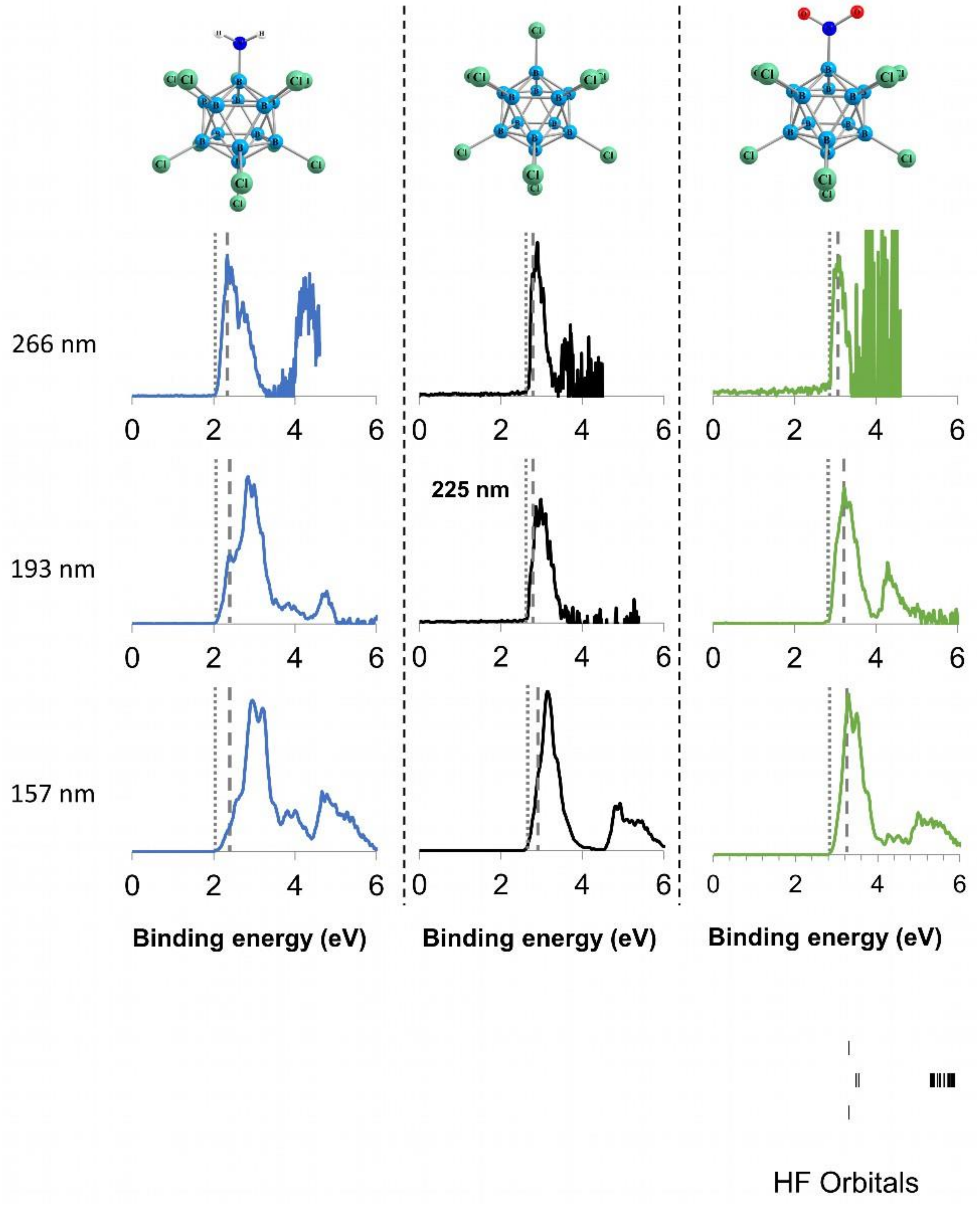

Figure S38: Photoelectron spectra of $[B_{12}Cl_{11}X]^{2-}$ (X = NH_2, Cl, NO_2) measured at different wavelengths. Dashed lines indicate vertical detachment energies (VDE) and dotted lines adiabatic detachment energies (ADE). Denotation of the density of states (DOS) predicted by Hartee-Fock calculations is shown below. Note that the highest occupied molecular orbital (HOMO) energy was shifted to match the experimental VDE.

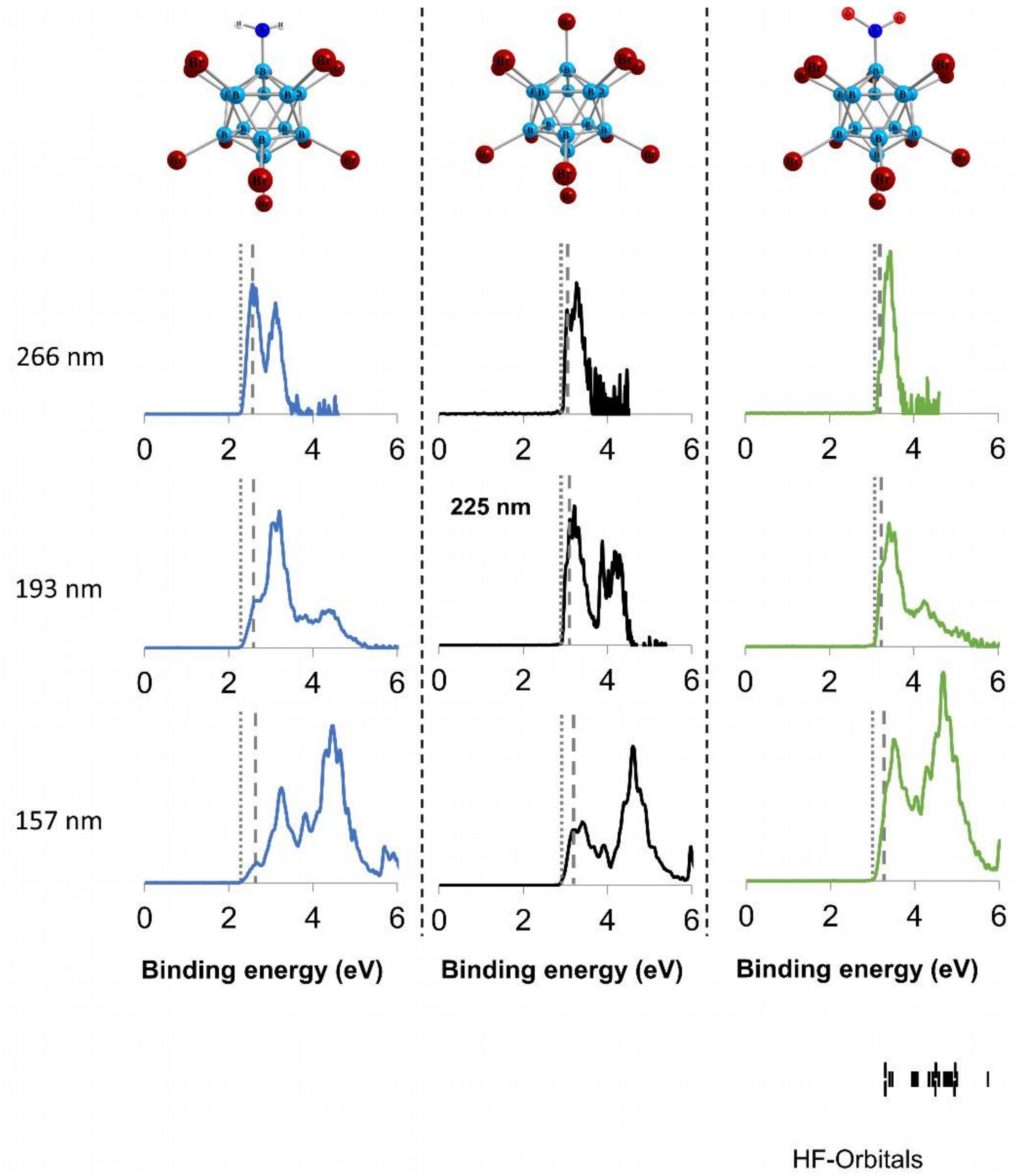

Figure S39: Photoelectron spectra of $[B_{12}Br_{11}X]^{2-}$ (X = NH_2, Br, NO_2) measured at different wavelengths. Dashed lines indicate vertical detachment energies (VDE) and dotted lines adiabatic detachment energies (ADE). Denotation of the density of states (DOS) predicted by Hartee-Fock calculations is shown below. Note that the highest occupied molecular orbital (HOMO) energy was shifted to match the experimental VDE.

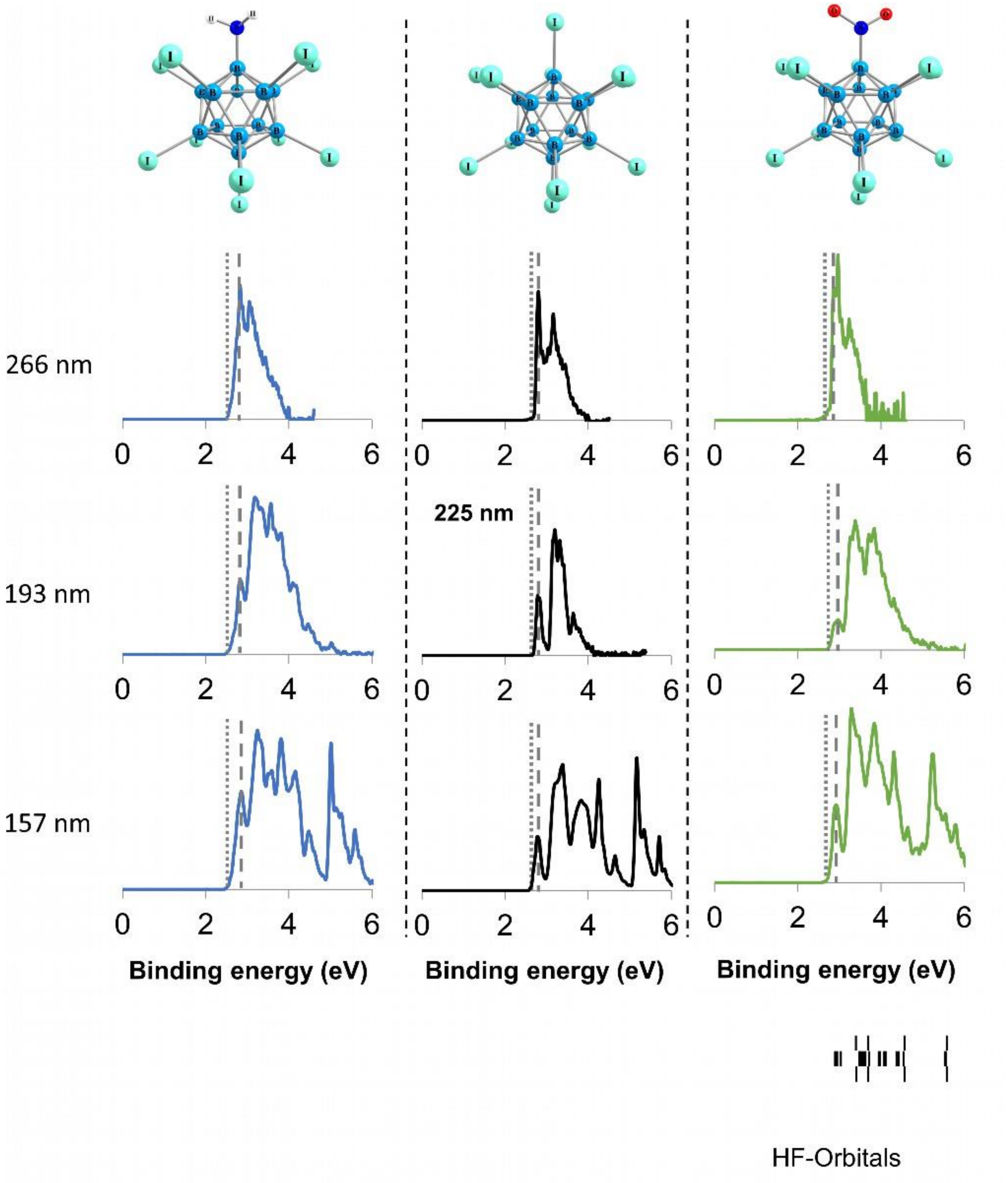

Figure S40: Photoelectron spectra of $[B_{12}I_{11}X]^{2-}$ (X = NH_2, I, NO_2) measured at different wavelengths. Dashed lines indicate vertical detachment energies (VDE) and dotted lines adiabatic detachment energies (ADE). Denotation of the density of states (DOS) predicted by Hartee-Fock calculations is shown below. Note that the highest occupied molecular orbital (HOMO) energy was shifted to match the experimental VDE.

Table S7: Measured adiabatic electron detachment energies in eV by photo electron spectroscopy at different wavelength. The corresponding vertical electron detachment energies are given in brackets. The Δ refers to the difference compared to the unsubstituted $[B_{12}X_{12}]^{2-}$.

$[B_{12}F_{11}X]^{2-}$	X = F	X = NH_2	X = NO_2
266 nm	1.50	1.04 (1.49)	1.95 (2.24)
193 nm	1.46	1.04 (1.47)	1.92 (2.20)
157 nm	1.50	1.06 (1.42)	1.94 (2.28)
Average	1.49	1.05 (1.46)	1.94 (2.24)
Δ	-	- 0.44	+ 0.45 (Calc.: + 0.39)[a]
$[B_{12}Cl_{11}X]^{2-}$	X = Cl	X = NH_2	X = NO_2
266 nm	2.69	2.05 (2.33)	2.88 (3.05)
193 nm	2.66	2.07 (2.39)	2.87 (3.21)
157 nm	2.64	2.11 (2.37)	2.87 (3.29)
Average	2.66	2.08 (2.36)	2.87 (3.18)
Δ	-	- 0.58	+ 0.21 (calc.: + 0.19)[a]
$[B_{12}Br_{11}X]^{2-}$	X = Br	X = NH_2	X = NO_2
266 nm	2.94	2.30 (2.55)	3.12 (3.23)
193 nm	2.93[b]	2.30 (2.62)	3.09 (3.25)
157 nm	2.92	2.30 (2.63)	3.08 (3.33)
Average	2.93	2.30 (2.60)	3.10 (3.27)
Δ	-	- 0.63	+ 0.17 (calc.: + 0.14)[a]
$[B_{12}I_{11}X]^{2-}$	X = I	X = NH_2	X = NO_2
266 nm	2.70	2.54 (2.82)	2.77 (2.88)
193 nm	2.70[b]	2.55 (2.82)	2.74 (2.94)
157 nm	2.65	2.55 (2.85)	2.73 (2.92)
Average	2.68	2.55 (2.83)	2.75 (2.91)
Δ	-	- 0.13	+ 0.07 (calc.: + 0.06)[a]

a = Calculated (PBE0/def2-TZVPP) difference to the value for $[B_{12}X_{12}]^{2-}$

b = Measured on 225 nm

The obtained differences could be well reproduced by quantum-chemical calculations using the equation:

$$\Delta ADE = (E\,[B_{12}X_{11}NO_2]^{-} - E\,[B_{12}X_{11}NO_2]^{2-}) - ADE\,[B_{12}X_{12}]^{2-}$$

S8 Quantum-chemical calculations

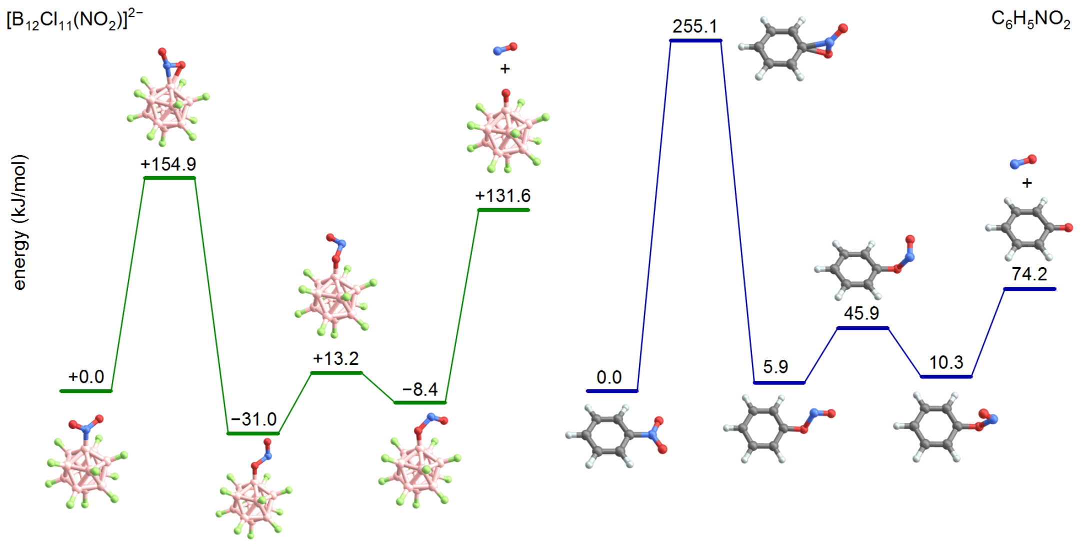

Figure S41: Calculated potential energy surface for the loss of NO• from $[B_{12}Cl_{11}(NO_2)]^{2-}$ and $C_6H_5NO_2$ calculated on the same computational level (B3LYP+GD3BJ/def2-TZVPP). The ONO-isomer is energetically more favorable in the case of the dodecaborate anion but not in the case of nitrobenzene.

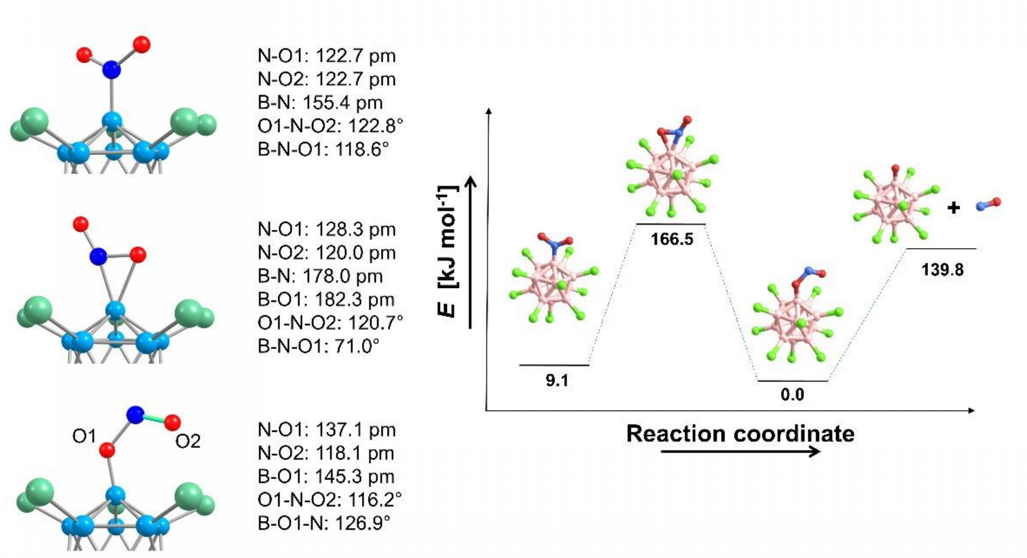

Figure S42: Selected calculated bond lengths and angles.

Table S8: Measured Comparison of the relative electronic energies for the three minimum structures for different combinations of DFT functions and basis sets.

Functional	B3LYP+GD3BJ			HSE06		
Basisset	def2-TZVPP	def2-TZVPPD	aug-cc-pVTZ	def2-TZVPP	def2-TZVPPD	aug-cc-pVTZ
$[B_{12}Cl_{11}(NO)_2]^{2-}$	0.0	0.0	0.0	0.0	0.0	0.0
$[B_{12}Cl_{11}(ONO)]^{2-}$ - h	-4.4	-4.8	-6.6	-0.8	-1.1	-2.7
$[B_{12}Cl_{11}(ONO)]^{2-}$ - s	-27.1	-27.6	-29.3	-24.5	-24.9	-26.4

Table S9: Comparison of the zero-point corrected relative electronic energies for the three minimum structures for different combinations of DFT functions and basis sets.

Functional	B3LYP+GD3BJ			HSE06		
Basisset	def2-TZVPP	def2-TZVPPD	aug-cc-pVTZ	def2-TZVPP	def2-TZVPPD	aug-cc-pVTZ
$[B_{12}Cl_{11}(NO)_2]^{2-}$	0.0	0.0	0.0	0.0	0.0	0.0
$[B_{12}Cl_{11}(ONO)]^{2-}$ - h	-8.4	-8.8	-10.6	-4.8	-5.1	-6.6
$[B_{12}Cl_{11}(ONO)]^{2-}$ - s	-31.0	-31.5	-33.3	-28.4	-28.9	-30.4

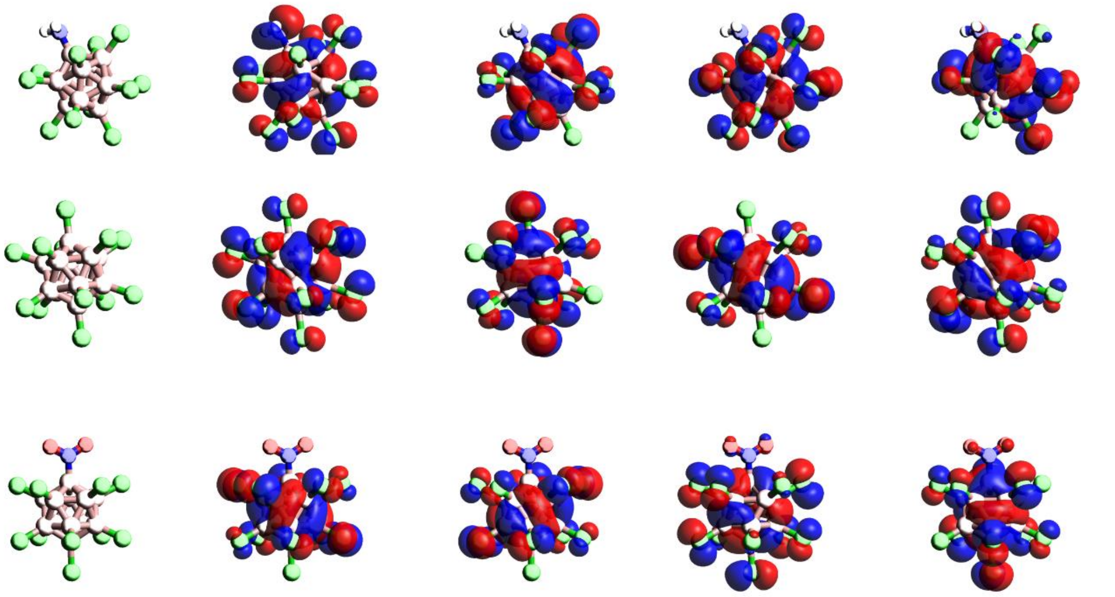

Figure S43: Visualization of the molecular structure and the first four energetically highest occupied molecular orbitals (HOMOs) of $[B_{12}Cl_{11}(NH_2)]^{2-}$ (top row), $[B_{12}Cl_{12}]^{2-}$ (middle row) and $[B_{12}Cl_{11}(NO_2)]^{2-}$ (bottom row). Visualized orbitals were calculated using Hartree Fock/aug-cc-pVTZ.

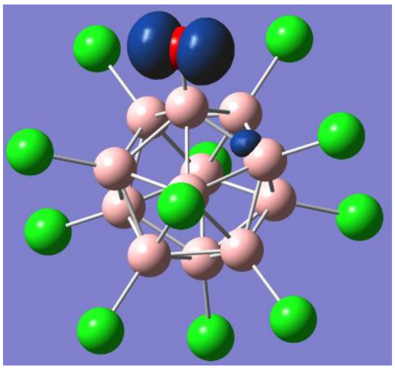

Figure S44: Calculated structure (B3LYP/def2-TZVPP) of the radical dianion $[B_{12}Cl_{11}O]^{2-\bullet}$ which will be mass selected and deposited. Calculated spin-density (0.01 u), is visualized in dark blue, showing that the radical is strongly localized on the oxygen atom. Therefore, the ion is expected to be very reactive.

Gas phase basicity

Table S10: Calculated (B3LYP+GD3BJ/aug-cc-pVTZ) gas-phase basicities *B* of $[B_{12}X_{11}(NH_2)]^{2-}$ (X =F-I) in [kJ mol^{-1}].

X	*B*
F	-1557.1
Cl	-1522.7
Br	-1514.4
I	-1504.0

$$[B_{12}X_{11}(NH_3)]^- + 3\ H_2O_2 + OH^- \longrightarrow [B_{12}X_{11}(NO_2)]^{2-} + 5\ H_2O \qquad \text{Eq. (1)}$$

Table S11. Calculated (B3LYP+GD3BJ/aug-cc-pVTZ) reaction enthalpies at 0 K and free reaction energies (PBE0/def2-TZVPP) in H_2O at 298 K referring to reaction Eq. (1) for X =F-I in [kJ mol^{-1}].

X	ΔH^0	ΔG^{298} (H_2O)
F	-701.6	-839.6
Cl	-692.1	-835.0
Br	-688.6	-828.6
I	-686.2	-815.2

S8.1 Calculated energies of all optimized structures

Table S12: Sum of electronic and zero-point Energies of the calculated structures.

	Compound	Energy [Eh]	Point group	NIMAG[a]
**PBE0/def2-TZVPP**	$[B_{12}F_{11}(NO_2)]^{2-}$	– 1601.467853	C_s	0
	$[B_{12}Cl_{11}(NO_2)]^{2-}$	– 5564.415146	C_s	0
	$[B_{12}Br_{11}(NO_2)]^{2-}$	– 28816.143062	C_s	0
	$[B_{12}I_{11}(NO_2)]^{2-}$	– 3778.571533	C_s	0
	$[B_{12}F_{11}(NO_2)]^{-}$	– 1601.405768	C_1	1 (-6 cm^{-1})[b]
	$[B_{12}Cl_{11}(NO_2)]^{-}$	– 5564.322427	C_1	0
	$[B_{12}Br_{11}(NO_2)]^{-}$	– 28816.040933	C_1	1 (-12 cm^{-1})[b]
	$[B_{12}I_{11}(NO_2)]^{-}$	– 3778.483809	C_s	0
	$[B_{12}F_{11}(NH_2)]^{2-}$	– 1452.369947	C_s	0
	$[B_{12}Cl_{11}(NH_2)]^{2-}$	– 5415.333587	C_s	0
	$[B_{12}Br_{11}(NH_2)]^{2-}$	– 28667.066940	C_s	0
	$[B_{12}I_{11}(NH_2)]^{2-}$	– 3629.500239	C_s	0
	$[B_{12}F_{11}(NH_2)]^{-}$	– 1452.343331	C_1	0
	$[B_{12}Cl_{11}(NH_2)]^{-}$	– 5415.272343	C_1	0
	$[B_{12}Br_{11}(NH_2)]^{-}$	– 28666.994110	C_1	0
	$[B_{12}I_{11}(NH_2)]^{-}$	– 3629.417847	C_1	0
**B3LYP+GD3BJ/def2-TZVPPD**	$[B_{12}Cl_{11}(NO_2)]^{2-}$	– 5566.898172	C_1	
	$[B_{12}Cl_{11}(NO_2)]^{2-‡}$	– 5566.826110	C_1	1 (-537 cm^{-1})
	$[B_{12}Cl_{11}(ONO)]^{2-}$	– 5566.901325	C_s	0
	$[B_{12}Cl_{11}O]^{2-}$	– 5436.893885	C_1	1 (-19 cm^{-1})[c]
	NO	– 129.9400001	$C_{\infty v}$	

a NIMAG = Number of imaginary frequencies

b The vibrations correspond to the rotation of the NO_2 group around the B-N bond.

c The vibrations correspond to the deformation vibration of the boron cluster core.

S8.2 Coordinates of all optimized structures

PBE0/def2-TZVPP

$[B_{12}F_{11}(NO_2)]^{2-}$ (C_s)

B	1.227085000000	-1.076758000000	0.896043000000
B	-0.479705000000	-1.055388000000	1.450059000000
B	-1.535588000000	-1.052398000000	0.000000000000
B	-0.479705000000	-1.055388000000	-1.450059000000
B	1.227085000000	-1.076758000000	-0.896043000000
B	0.483792000000	0.458622000000	1.452267000000
B	-1.238557000000	0.469898000000	0.897055000000
B	-1.238557000000	0.469898000000	-0.897055000000
B	0.483792000000	0.458622000000	-1.452267000000
B	1.546896000000	0.440224000000	0.000000000000
B	0.015245000000	1.370378000000	0.000000000000
B	-0.017567000000	-2.004768000000	0.000000000000
N	0.011910000000	2.919439000000	0.000000000000
F	0.859622000000	1.069789000000	2.626525000000
F	2.798111000000	1.004698000000	0.000000000000
F	2.217355000000	-1.703892000000	1.622495000000
F	2.217355000000	-1.703892000000	-1.622495000000
F	0.859622000000	1.069789000000	-2.626525000000
F	-0.866145000000	-1.662121000000	-2.626543000000
F	-2.219550000000	1.078352000000	-1.641954000000
F	-2.219550000000	1.078352000000	1.641954000000
F	-2.771813000000	-1.663676000000	0.000000000000
F	-0.866145000000	-1.662121000000	2.626543000000
F	-0.031309000000	-3.383393000000	0.000000000000
O	1.079630000000	3.513708000000	0.000000000000
O	-1.061185000000	3.503297000000	0.000000000000

$[B_{12}Cl_{11}(NO_2)]^{2-}$ (C_s)

B	-1.202318000000	-0.879516000000	0.892442000000
B	-1.202318000000	-0.879516000000	-0.892442000000
B	0.493270000000	-0.823176000000	-1.444131000000
B	1.542618000000	-0.797998000000	0.000000000000
B	0.493270000000	-0.823176000000	1.444131000000
B	-1.553736000000	0.624935000000	0.000000000000
B	-0.494908000000	0.668646000000	-1.444187000000
B	1.216326000000	0.713028000000	-0.894057000000
B	1.216326000000	0.713028000000	0.894057000000
B	-0.494908000000	0.668646000000	1.444187000000
B	-0.048344000000	1.596500000000	0.000000000000
B	0.053957000000	-1.778458000000	0.000000000000
N	-0.077749000000	3.152724000000	0.000000000000
Cl	-2.469050000000	-1.721382000000	1.838560000000
Cl	1.009105000000	-1.605173000000	2.970964000000

Cl	3.166460000000	-1.554165000000	0.000000000000
Cl	2.489221000000	1.517218000000	-1.860189000000
Cl	2.489221000000	1.517218000000	1.860189000000
Cl	-1.008000000000	1.462021000000	2.966439000000
Cl	-1.008000000000	1.462021000000	-2.966439000000
Cl	-3.210105000000	1.298741000000	0.000000000000
Cl	-2.469050000000	-1.721382000000	-1.838560000000
Cl	1.009105000000	-1.605173000000	-2.970964000000
Cl	0.108722000000	-3.568909000000	0.000000000000
O	-1.156803000000	3.717951000000	0.000000000000
O	0.984104000000	3.749376000000	0.000000000000

$[B_{12}Br_{11}(NO_2)]^{2-}$ (C_s)

B	-0.644361000000	1.234449000000	0.892877000000
B	-0.644361000000	1.234449000000	-0.892877000000
B	-0.657423000000	-0.462265000000	-1.444775000000
B	-0.675931000000	-1.511926000000	0.000000000000
B	-0.657423000000	-0.462265000000	1.444775000000
B	0.872958000000	1.523280000000	0.000000000000
B	0.874129000000	0.463481000000	-1.443215000000
B	0.847423000000	-1.247320000000	-0.893768000000
B	0.847423000000	-1.247320000000	0.893768000000
B	0.874129000000	0.463481000000	1.443215000000
B	1.786688000000	-0.020457000000	0.000000000000
B	-1.594282000000	0.015911000000	0.000000000000
N	3.344986000000	-0.054270000000	0.000000000000
O	3.951681000000	1.000599000000	0.000000000000
O	3.895510000000	-1.139351000000	0.000000000000
Br	-3.545779000000	0.036341000000	0.000000000000
Br	-1.506327000000	2.648724000000	-1.925146000000
Br	-1.533467000000	-0.987264000000	-3.107895000000
Br	-1.572462000000	-3.245346000000	0.000000000000
Br	-1.506327000000	2.648724000000	1.925146000000
Br	-1.533467000000	-0.987264000000	3.107895000000
Br	1.771538000000	0.987182000000	3.095782000000
Br	1.668559000000	-2.671077000000	1.943490000000
Br	1.679427000000	3.298804000000	0.000000000000
Br	1.771538000000	0.987182000000	-3.095782000000
Br	1.668559000000	-2.671077000000	-1.943490000000

$[B_{12}I_{11}(NO_2)]^{2-}$ (C_s)

B	-0.568757000000	1.231800000000	0.894808000000
B	-0.568757000000	1.231800000000	-0.894808000000
B	-0.569920000000	-0.468535000000	-1.448051000000
B	-0.582081000000	-1.520174000000	0.000000000000
B	-0.569920000000	-0.468535000000	1.448051000000
B	0.948472000000	1.530750000000	0.000000000000

B	0.957124000000	0.469124000000	-1.444989000000
B	0.941628000000	-1.244439000000	-0.894847000000
B	0.941628000000	-1.244439000000	0.894847000000
B	0.957124000000	0.469124000000	1.444989000000
B	1.873225000000	-0.009008000000	0.000000000000
B	-1.512093000000	0.004383000000	0.000000000000
N	3.434837000000	-0.031262000000	0.000000000000
O	4.033735000000	1.027377000000	0.000000000000
O	3.991562000000	-1.112592000000	0.000000000000
I	1.966987000000	1.057430000000	-3.269555000000
I	-1.537788000000	2.793523000000	-2.043346000000
I	-1.537788000000	2.793523000000	2.043346000000
I	1.839321000000	3.507153000000	0.000000000000
I	-3.679006000000	0.012357000000	0.000000000000
I	-1.539572000000	-1.056206000000	-3.295212000000
I	1.874336000000	-2.819466000000	-2.053789000000
I	-1.565307000000	-3.451369000000	0.000000000000
I	1.874336000000	-2.819466000000	2.053789000000
I	-1.539572000000	-1.056206000000	3.295212000000
I	1.966987000000	1.057430000000	3.269555000000

$[B_{12}F_{11}(NO_2)]^-$ (C_1)

B	-1.078634000000	1.211772000000	-0.926594000000
B	-1.087436000000	-0.529079000000	-1.438917000000
B	-1.111354000000	-1.536166000000	0.017183000000
B	-1.089491000000	-0.501327000000	1.449647000000
B	-1.078565000000	1.225692000000	0.910616000000
B	0.490433000000	0.510805000000	-1.451031000000
B	0.470490000000	-1.226898000000	-0.913850000000
B	0.470399000000	-1.213221000000	0.928804000000
B	0.488525000000	0.539118000000	1.439227000000
B	0.496116000000	1.553259000000	-0.017674000000
B	1.394023000000	0.016762000000	-0.001785000000
B	-2.037203000000	-0.009860000000	0.001237000000
N	2.937714000000	-0.024642000000	0.001450000000
F	1.060538000000	0.851194000000	-2.627212000000
F	1.013158000000	2.800656000000	-0.028021000000
F	-1.685866000000	2.180540000000	-1.632721000000
F	-1.684010000000	2.209880000000	1.598094000000
F	1.064153000000	0.897080000000	2.606475000000
F	-1.655398000000	-0.845785000000	2.628951000000
F	1.061642000000	-2.178366000000	1.645593000000
F	1.059632000000	-2.208010000000	-1.611762000000
F	-1.661738000000	-2.774740000000	0.026624000000
F	-1.659200000000	-0.891928000000	-2.608918000000
F	-3.396928000000	0.027742000000	-0.001444000000
O	3.538892000000	1.033892000000	0.002825000000
O	3.480561000000	-1.114659000000	0.002752000000

$[B_{12}Cl_{11}(NO_2)]^-$ (C_1)

B	0.809684000000	1.496770000000	-0.407551000000
B	0.858954000000	0.816829000000	1.281421000000
B	0.890878000000	-0.990389000000	1.134239000000
B	0.898325000000	-1.380682000000	-0.604735000000
B	0.833338000000	0.108898000000	-1.535822000000
B	-0.726171000000	1.397046000000	0.607699000000
B	-0.680373000000	-0.107545000000	1.536868000000
B	-0.637459000000	-1.504693000000	0.412858000000
B	-0.697993000000	-0.820697000000	-1.282073000000
B	-0.727815000000	1.004219000000	-1.132419000000
B	-1.608231000000	-0.045147000000	0.023011000000
B	1.797019000000	0.051348000000	-0.024474000000
N	-3.158895000000	-0.082602000000	0.015112000000
Cl	1.551947000000	3.049284000000	-0.790228000000
Cl	1.568965000000	0.246762000000	-3.138904000000
Cl	1.694407000000	-2.834504000000	-1.218836000000
Cl	-1.316541000000	-3.080525000000	0.802113000000
Cl	-1.482696000000	-1.663796000000	-2.607402000000
Cl	-1.547249000000	1.988169000000	-2.344725000000
Cl	-1.430513000000	-0.243717000000	3.134455000000
Cl	-1.463800000000	2.868981000000	1.243271000000
Cl	1.646199000000	1.658736000000	2.607960000000
Cl	1.722213000000	-1.977243000000	2.337706000000
Cl	3.571592000000	0.104108000000	-0.040954000000
O	-3.754542000000	0.976902000000	0.042184000000
O	-3.706132000000	-1.167890000000	-0.028016000000

$[B_{12}Br_{11}(NO_2)]^-$ (C_1)

B	-1.214355000000	0.900632000000	0.697874000000
B	-1.195380000000	-0.924998000000	0.698390000000
B	0.528836000000	-1.441040000000	0.649396000000
B	1.536431000000	0.016440000000	0.655193000000
B	0.498628000000	1.451856000000	0.649357000000
B	-1.550248000000	-0.016572000000	-0.847381000000
B	-0.504863000000	-1.451135000000	-0.863594000000
B	1.219293000000	-0.902467000000	-0.899636000000
B	1.200291000000	0.926715000000	-0.900325000000
B	-0.535322000000	1.440167000000	-0.863471000000
B	-0.029255000000	-0.000426000000	-1.793859000000
B	0.026337000000	0.000423000000	1.609242000000
N	-0.046657000000	-0.000364000000	-3.348531000000
O	-1.121425000000	-0.005313000000	-3.914959000000
O	1.023721000000	0.005006000000	-3.925366000000
Br	0.057497000000	0.000966000000	3.545297000000
Br	-2.548721000000	-1.954981000000	1.595337000000
Br	1.097098000000	-3.082108000000	1.492961000000
Br	3.284787000000	0.034868000000	1.469753000000

Br	-2.588880000000	1.902623000000	1.594459000000
Br	1.033027000000	3.104741000000	1.492082000000
Br	-1.103509000000	3.073034000000	-1.726117000000
Br	2.566205000000	1.964549000000	-1.762655000000
Br	-3.330778000000	-0.035533000000	-1.580237000000
Br	-1.039337000000	-3.095536000000	-1.726069000000
Br	2.607075000000	-1.912421000000	-1.760344000000

$[B_{12}I_{11}(NO_2)]^-$ (C_s)

B	-0.475052000000	0.575315000000	1.439638000000
B	1.215593000000	0.568223000000	0.886475000000
B	1.215593000000	0.568223000000	-0.886475000000
B	-0.475052000000	0.575315000000	-1.439638000000
B	-1.523036000000	0.565544000000	0.000000000000
B	0.465348000000	-0.939879000000	1.448553000000
B	1.512040000000	-0.955690000000	0.000000000000
B	0.465348000000	-0.939879000000	-1.448553000000
B	-1.225493000000	-0.948384000000	-0.895087000000
B	-1.225493000000	-0.948384000000	0.895087000000
B	-0.002159000000	-1.874051000000	0.000000000000
B	-0.009227000000	1.517749000000	0.000000000000
N	-0.016926000000	-3.431333000000	0.000000000000
O	-0.027262000000	-4.005036000000	1.071529000000
O	-0.027262000000	-4.005036000000	-1.071529000000
I	3.421883000000	-1.959718000000	0.000000000000
I	2.791415000000	1.527943000000	2.005400000000
I	-1.078940000000	1.546154000000	3.269211000000
I	1.093218000000	-1.823537000000	3.314206000000
I	-0.002461000000	3.674414000000	0.000000000000
I	2.791415000000	1.527943000000	-2.005400000000
I	1.093218000000	-1.823537000000	-3.314206000000
I	-1.078940000000	1.546154000000	-3.269211000000
I	-2.777006000000	-1.928371000000	-2.027952000000
I	-3.460521000000	1.514121000000	0.000000000000
I	-2.777006000000	-1.928371000000	2.027952000000

$[B_{12}F_{11}(NH_2)]^{2-}$ (C_s)

B	1.410823000000	-0.363928000000	0.896588000000
B	-0.218900000000	-0.879485000000	1.450681000000
B	-1.218818000000	-1.208782000000	0.000000000000
B	-0.218900000000	-0.879485000000	-1.450681000000
B	1.410823000000	-0.363928000000	-0.896588000000
B	0.209234000000	0.858462000000	1.436784000000
B	-1.414835000000	0.335757000000	0.891555000000
B	-1.414835000000	0.335757000000	-0.891555000000
B	0.209234000000	0.858462000000	-1.436784000000
B	1.216554000000	1.165841000000	0.000000000000
B	-0.529180000000	1.615068000000	0.000000000000

B	0.520502000000	-1.635371000000	0.000000000000
N	-0.964614000000	3.046237000000	0.000000000000
H	-0.603086000000	3.530273000000	-0.811667000000
H	-0.603086000000	3.530273000000	0.811667000000
F	0.944290000000	-2.952246000000	0.000000000000
F	-0.391551000000	-1.585257000000	2.628136000000
F	2.546937000000	-0.653921000000	1.631392000000
F	0.383916000000	1.590601000000	2.603658000000
F	-2.550200000000	0.604132000000	1.634149000000
F	-2.202622000000	-2.181253000000	0.000000000000
F	2.185680000000	2.158387000000	0.000000000000
F	-2.550200000000	0.604132000000	-1.634149000000
F	-0.391551000000	-1.585257000000	-2.628136000000
F	0.383916000000	1.590601000000	-2.603658000000
F	2.546937000000	-0.653921000000	-1.631392000000

$[B_{12}Cl_{11}(NH_2)]^{2-}$ (C_s)

B	0.711571000000	1.194592000000	0.891771000000
B	0.620000000000	-0.503875000000	1.444560000000
B	0.578763000000	-1.550880000000	0.000000000000
B	0.620000000000	-0.503875000000	-1.444560000000
B	0.711571000000	1.194592000000	-0.891771000000
B	-0.850787000000	0.504396000000	1.428248000000
B	-0.929427000000	-1.190928000000	0.886468000000
B	-0.929427000000	-1.190928000000	-0.886468000000
B	-0.850787000000	0.504396000000	-1.428248000000
B	-0.782114000000	1.551954000000	0.000000000000
B	-1.838299000000	0.082999000000	0.000000000000
B	1.586792000000	-0.079660000000	0.000000000000
N	-3.306298000000	0.155462000000	0.000000000000
H	-3.662599000000	0.627672000000	-0.819515000000
H	-3.662599000000	0.627672000000	0.819515000000
Cl	1.575571000000	2.447437000000	1.844451000000
Cl	1.575571000000	2.447437000000	-1.844451000000
Cl	1.395637000000	-1.032755000000	-2.974976000000
Cl	-1.789662000000	-2.447090000000	-1.840908000000
Cl	-1.663970000000	1.046696000000	-2.943597000000
Cl	-1.534054000000	3.187883000000	0.000000000000
Cl	-1.789662000000	-2.447090000000	1.840908000000
Cl	-1.663970000000	1.046696000000	2.943597000000
Cl	1.395637000000	-1.032755000000	2.974976000000
Cl	1.309328000000	-3.190137000000	0.000000000000
Cl	3.379573000000	-0.167938000000	0.000000000000

$[B_{12}Br_{11}(NH_2)]^{2-}$ (C_s)

B	-0.579674000000	-1.219198000000	0.891712000000
B	-0.540511000000	0.481637000000	1.444955000000
B	-0.533058000000	1.529333000000	0.000000000000
B	-0.540511000000	0.481637000000	-1.444955000000

B	-0.579674000000	-1.219198000000	-0.891712000000
B	0.960719000000	-0.480570000000	1.427334000000
B	0.985402000000	1.215698000000	0.885583000000
B	0.985402000000	1.215698000000	-0.885583000000
B	0.960719000000	-0.480570000000	-1.427334000000
B	0.923900000000	-1.529979000000	0.000000000000
B	1.943201000000	-0.029407000000	0.000000000000
B	-1.495146000000	0.027309000000	0.000000000000
N	3.405710000000	-0.043828000000	0.000000000000
H	3.793649000000	-0.483148000000	-0.822833000000
H	3.793649000000	-0.483148000000	0.822833000000
Br	-1.401177000000	1.030107000000	3.113074000000
Br	1.870627000000	-1.039685000000	3.074691000000
Br	1.892231000000	2.608189000000	1.919240000000
Br	-1.474680000000	-2.614562000000	1.929020000000
Br	-3.449824000000	0.061018000000	0.000000000000
Br	-1.474680000000	-2.614562000000	-1.929020000000
Br	-1.381614000000	3.290392000000	0.000000000000
Br	1.892231000000	2.608189000000	-1.919240000000
Br	-1.401177000000	1.030107000000	-3.113074000000
Br	1.870627000000	-1.039685000000	-3.074691000000
Br	1.803689000000	-3.282046000000	0.000000000000

$[B_{12}I_{11}(NH_2)]^{2-}$ (C_s)

B	-0.526559000000	-1.227282000000	0.893006000000
B	-0.502749000000	0.477248000000	1.447704000000
B	-0.506137000000	1.526431000000	0.000000000000
B	-0.502749000000	0.477248000000	-1.447704000000
B	-0.526559000000	-1.227282000000	-0.893006000000
B	1.008651000000	-0.473559000000	1.429265000000
B	1.016758000000	1.225421000000	0.886415000000
B	1.016758000000	1.225421000000	-0.886415000000
B	1.008651000000	-0.473559000000	-1.429265000000
B	0.981638000000	-1.525636000000	0.000000000000
B	1.994421000000	-0.012722000000	0.000000000000
B	-1.456578000000	0.012946000000	0.000000000000
N	3.451197000000	-0.000221000000	0.000000000000
H	3.863852000000	-0.409799000000	-0.825673000000
H	3.863852000000	-0.409799000000	0.825673000000
I	1.988054000000	-3.452187000000	0.000000000000
I	2.033162000000	-1.079152000000	3.251885000000
I	-1.500630000000	-2.788270000000	2.043580000000
I	-3.625689000000	0.029849000000	0.000000000000
I	-1.500630000000	-2.788270000000	-2.043580000000
I	-1.459190000000	1.077693000000	-3.300908000000
I	2.033162000000	-1.079152000000	-3.251885000000
I	2.032966000000	2.771000000000	-2.022282000000
I	-1.460146000000	3.474849000000	0.000000000000
I	2.032966000000	2.771000000000	2.022282000000
I	-1.459190000000	1.077693000000	3.300908000000

$[B_{12}F_{11}(NH_2)]^-$ (C_1)

B	-0.698512000000	-0.918864000000	-1.284006000000
B	-0.810359000000	-1.479523000000	0.406227000000
B	-0.841611000000	-0.010979000000	1.498506000000
B	-0.805197000000	1.475808000000	0.429283000000
B	-0.695811000000	0.942054000000	-1.269091000000
B	0.772182000000	-1.470091000000	-0.426341000000
B	0.658037000000	-0.946781000000	1.269270000000
B	0.660683000000	0.923882000000	1.284503000000
B	0.777471000000	1.473673000000	-0.403387000000
B	0.800772000000	0.011218000000	-1.509949000000
B	1.679292000000	-0.003624000000	0.093823000000
B	-1.712027000000	0.003727000000	-0.095065000000
N	3.102165000000	-0.006446000000	0.164199000000
H	3.643237000000	0.840797000000	0.170400000000
H	3.640081000000	-0.855696000000	0.168543000000
F	-3.063968000000	0.006398000000	-0.170285000000
F	-1.468281000000	-2.625320000000	0.747388000000
F	-1.258951000000	-1.607226000000	-2.313555000000
F	1.461683000000	-2.599770000000	-0.780450000000
F	1.241467000000	-1.643115000000	2.280548000000
F	-1.517841000000	-0.019052000000	2.675134000000
F	1.498811000000	0.019066000000	-2.670530000000
F	1.245943000000	1.602122000000	2.306651000000
F	-1.458812000000	2.618167000000	0.789386000000
F	1.471474000000	2.606569000000	-0.738610000000
F	-1.254088000000	1.648553000000	-2.287588000000

$[B_{12}Cl_{11}(NH_2)]^-$ (C_1)

B	0.908710000000	-1.230023000000	0.661186000000
B	1.464889000000	0.459630000000	0.657811000000
B	0.009877000000	1.537426000000	0.628961000000
B	-1.459185000000	0.476478000000	0.658492000000
B	-0.923446000000	-1.218717000000	0.662452000000
B	1.454098000000	-0.474176000000	-0.858311000000
B	0.930416000000	1.219933000000	-0.860557000000
B	-0.916347000000	1.231797000000	-0.859217000000
B	-1.459771000000	-0.457442000000	-0.857597000000
B	-0.010310000000	-1.554034000000	-0.824970000000
B	-0.000876000000	-0.017202000000	-1.800487000000
B	0.000659000000	0.014687000000	1.596027000000
N	-0.002114000000	-0.043894000000	-3.221775000000
H	-0.857296000000	-0.081809000000	-3.752858000000
H	0.852008000000	-0.071118000000	-3.755138000000
Cl	1.830364000000	-2.520027000000	1.455712000000
Cl	-1.859578000000	-2.497634000000	1.458410000000
Cl	-2.962639000000	0.990632000000	1.457634000000
Cl	-1.823206000000	2.509237000000	-1.694646000000
Cl	-2.944350000000	-0.943566000000	-1.725863000000
Cl	-0.019874000000	-3.136215000000	-1.619313000000

Cl	1.851862000000	2.486090000000	-1.698124000000
Cl	2.932493000000	-0.978486000000	-1.726119000000
Cl	2.975284000000	0.954496000000	1.456278000000
Cl	0.019698000000	3.135686000000	1.391744000000
Cl	0.001508000000	0.030282000000	3.364375000000

$[B_{12}Br_{11}(NH_2)]^-$ (C_1)

B	-0.916165000000	1.226448000000	0.567157000000
B	-1.458738000000	-0.468547000000	0.571294000000
B	0.004332000000	-1.531560000000	0.550646000000
B	1.461105000000	-0.461100000000	0.570956000000
B	0.909517000000	1.231337000000	0.566610000000
B	-1.455407000000	0.458967000000	-0.948016000000
B	-0.917315000000	-1.232734000000	-0.942776000000
B	0.923617000000	-1.227550000000	-0.943332000000
B	1.453072000000	0.466391000000	-0.948332000000
B	-0.004570000000	1.549683000000	-0.925241000000
B	-0.000307000000	0.008207000000	-1.890483000000
B	0.000151000000	-0.004883000000	1.507897000000
N	-0.000838000000	0.025615000000	-3.313474000000
H	0.855904000000	0.049344000000	-3.844867000000
H	-0.858010000000	0.056857000000	-3.843828000000
Br	-3.104865000000	-1.014860000000	1.438732000000
Br	-3.069132000000	0.986096000000	-1.903610000000
Br	-1.910377000000	-2.623578000000	-1.856453000000
Br	-1.939738000000	2.628717000000	1.419918000000
Br	0.000434000000	-0.011067000000	3.438320000000
Br	1.925780000000	2.639039000000	1.418891000000
Br	0.009163000000	-3.273825000000	1.384565000000
Br	1.923982000000	-2.612842000000	-1.857912000000
Br	3.110238000000	-0.998332000000	1.438593000000
Br	3.063905000000	1.002738000000	-1.903169000000
Br	-0.009062000000	3.267663000000	-1.812130000000

$[B_{12}I_{11}(NH_2)]^-$ (C_1)

B	-1.280618000000	-0.816501000000	0.515999000000
B	0.379531000000	-1.462431000000	0.503595000000
B	1.501935000000	-0.086263000000	0.517472000000
B	0.544390000000	1.409063000000	0.504450000000
B	-1.179047000000	0.957422000000	0.516260000000
B	-0.554358000000	-1.392689000000	-1.011907000000
B	1.160423000000	-0.946126000000	-1.002961000000
B	1.261365000000	0.807939000000	-1.002423000000
B	-0.391592000000	1.447443000000	-1.011567000000
B	-1.520182000000	0.087436000000	-0.991405000000
B	-0.010942000000	0.001140000000	-1.999213000000
B	-0.005486000000	-0.000039000000	1.463252000000

N	0.030586000000	-0.000747000000	-3.448816000000
H	-0.291851000000	0.845382000000	-3.893496000000
H	-0.379841000000	-0.807664000000	-3.893888000000
I	-3.430305000000	0.196911000000	-1.996803000000
I	-1.235579000000	-3.179036000000	-2.029563000000
I	-2.896918000000	-1.881194000000	1.475435000000
I	0.000373000000	-0.001068000000	3.622183000000
I	-2.662994000000	2.199097000000	1.476629000000
I	1.254322000000	3.216601000000	1.451031000000
I	-0.862642000000	3.300874000000	-2.028144000000
I	2.881859000000	1.829248000000	-2.001995000000
I	3.437706000000	-0.197858000000	1.470111000000
I	2.653810000000	-2.145306000000	-2.003387000000
I	0.877925000000	-3.339484000000	1.449812000000

<u>B3LYP+GD3BJ/def2-TZVPPD</u>

$[B_{12}Cl_{11}(NO_2)]^{2-}$ (C_1)

B	-0.649941000000	1.462403000000	-0.473473000000
B	-0.628761000000	0.024328000000	-1.538825000000
B	0.879937000000	-0.872767000000	-1.208816000000
B	0.779911000000	-0.024294000000	1.542407000000
B	-0.719219000000	0.880997000000	1.211782000000
B	-0.721851000000	-0.914482000000	1.185349000000
B	-0.655121000000	-1.445048000000	-0.516521000000
B	0.883254000000	0.905515000000	-1.182247000000
B	0.827873000000	1.430997000000	0.518107000000
B	1.773810000000	-0.003976000000	0.063058000000
B	0.823075000000	-1.448619000000	0.475129000000
B	-1.591566000000	0.003543000000	-0.056631000000
Cl	-1.367318000000	3.022038000000	-0.996614000000
Cl	-1.390587000000	0.049671000000	-3.167851000000
Cl	1.728485000000	-1.794358000000	-2.498584000000
Cl	1.520731000000	-0.049760000000	3.180591000000
Cl	-1.565158000000	1.798470000000	2.504083000000
Cl	-1.568722000000	-1.867210000000	2.451047000000
Cl	-1.380061000000	-2.984616000000	-1.086696000000
Cl	1.735338000000	1.862070000000	-2.443907000000
Cl	1.620156000000	2.947778000000	1.069934000000
Cl	3.570565000000	-0.008018000000	0.127590000000
Cl	1.610239000000	-2.983969000000	0.981007000000
O	-3.731477000000	1.083800000000	-0.095696000000
N	-3.145275000000	0.005695000000	-0.088538000000
O	-3.733829000000	-1.071110000000	-0.095187000000

$[B_{12}Cl_{11}(NO_2)]^{2-‡}$ (C_1)

B	-0.233038000000	1.405171000000	-0.886846000000
B	-0.780209000000	-0.213383000000	-1.426144000000
B	0.451109000000	-1.404798000000	-0.888327000000
B	0.956293000000	0.212655000000	1.440255000000
B	-0.233079000000	1.405148000000	0.886864000000
B	-0.780269000000	-0.213418000000	1.426100000000
B	-1.065602000000	-1.235903000000	-0.000040000000
B	0.956356000000	0.212689000000	-1.440210000000
B	1.297915000000	1.198293000000	0.000042000000
B	1.714049000000	-0.524948000000	0.000029000000
B	0.451072000000	-1.404819000000	0.888311000000
B	-1.524952000000	0.521658000000	-0.000032000000
Cl	-0.573149000000	2.885839000000	-1.848590000000
Cl	-1.712893000000	-0.433466000000	-2.952535000000
Cl	0.819566000000	-2.879886000000	-1.850271000000
Cl	1.864551000000	0.430449000000	2.978474000000
Cl	-0.573235000000	2.885795000000	1.848626000000
Cl	-1.713024000000	-0.433538000000	2.952443000000
Cl	-2.307802000000	-2.533800000000	-0.000080000000
Cl	1.864680000000	0.430518000000	-2.978385000000
Cl	2.560916000000	2.479287000000	0.000084000000
Cl	3.426460000000	-1.076518000000	0.000060000000
Cl	0.819486000000	-2.879931000000	1.850233000000
O	-2.897347000000	1.722064000000	-0.000053000000
N	-3.304784000000	0.505808000000	-0.000039000000
O	-4.477551000000	0.252547000000	-0.000036000000

$[B_{12}Cl_{11}(ONO)]^{2-}$ (C_s)

B	-1.697099000000	-0.196379000000	0.000000000000
B	-0.788542000000	0.322533000000	1.439837000000
B	0.764749000000	-0.537477000000	1.438537000000
B	0.764749000000	-0.537477000000	-1.438537000000
B	-0.788542000000	0.322533000000	-1.439837000000
B	0.683680000000	1.167329000000	-0.896357000000
B	0.683680000000	1.167329000000	0.896357000000
B	-0.705966000000	-1.370531000000	0.890482000000
B	-0.705966000000	-1.370531000000	-0.890482000000
B	0.819985000000	-1.581503000000	0.000000000000
B	1.672763000000	-0.015844000000	0.000000000000
B	-0.849696000000	1.380417000000	0.000000000000
Cl	-3.495355000000	-0.284842000000	0.000000000000
Cl	-1.627285000000	0.794093000000	2.962485000000
Cl	1.594515000000	-0.995832000000	2.968092000000
Cl	1.594515000000	-0.995832000000	-2.968092000000
Cl	-1.627285000000	0.794093000000	-2.962485000000
Cl	1.470981000000	2.436227000000	-1.903991000000
Cl	1.470981000000	2.436227000000	1.903991000000
Cl	-1.446939000000	-2.708422000000	1.838924000000
Cl	-1.446939000000	-2.708422000000	-1.838924000000

Cl	1.710077000000	-3.145187000000	0.000000000000
Cl	3.470332000000	0.072656000000	0.000000000000
O	-1.794979000000	2.484830000000	0.000000000000
N	-1.496004000000	3.821775000000	0.000000000000
O	-0.348289000000	4.100755000000	0.000000000000

$[B_{12}Cl_{11}(O)]^{2-\bullet}$ (C_1)

B	0.481635000000	-1.432795000000	-0.906734000000
B	-1.247359000000	-0.877931000000	-0.889873000000
B	-1.517212000000	0.000096000000	0.633985000000
B	1.231847000000	0.884016000000	0.622177000000
B	1.532756000000	-0.000277000000	-0.878311000000
B	0.480901000000	1.431659000000	-0.908919000000
B	-1.247807000000	0.875934000000	-0.891213000000
B	-0.474361000000	-1.438891000000	0.601958000000
B	1.232298000000	-0.882437000000	0.623526000000
B	-0.000719000000	0.001185000000	1.553609000000
B	-0.475098000000	1.439562000000	0.599761000000
B	0.000719000000	-0.001444000000	-1.892226000000
Cl	0.974179000000	-2.976048000000	-1.687655000000
Cl	-2.542338000000	-1.840070000000	-1.687364000000
Cl	-3.117976000000	0.000316000000	1.457612000000
Cl	2.516154000000	1.847664000000	1.434545000000
Cl	3.143244000000	-0.000480000000	-1.683437000000
Cl	0.972656000000	2.973970000000	-1.692191000000
Cl	-2.543274000000	1.836197000000	-1.690169000000
Cl	-0.958929000000	-2.976671000000	1.399243000000
Cl	2.517096000000	-1.844190000000	1.437361000000
Cl	-0.001675000000	0.002556000000	3.351458000000
Cl	-0.960451000000	2.978308000000	1.394702000000
O	0.004295000000	-0.002471000000	-3.239810000000

NO• ($C_{\infty v}$)

N	0.000000000000	0.000000000000	-0.610628000000
O	0.000000000000	0.000000000000	0.534300000000

S9 Gas phase chemistry

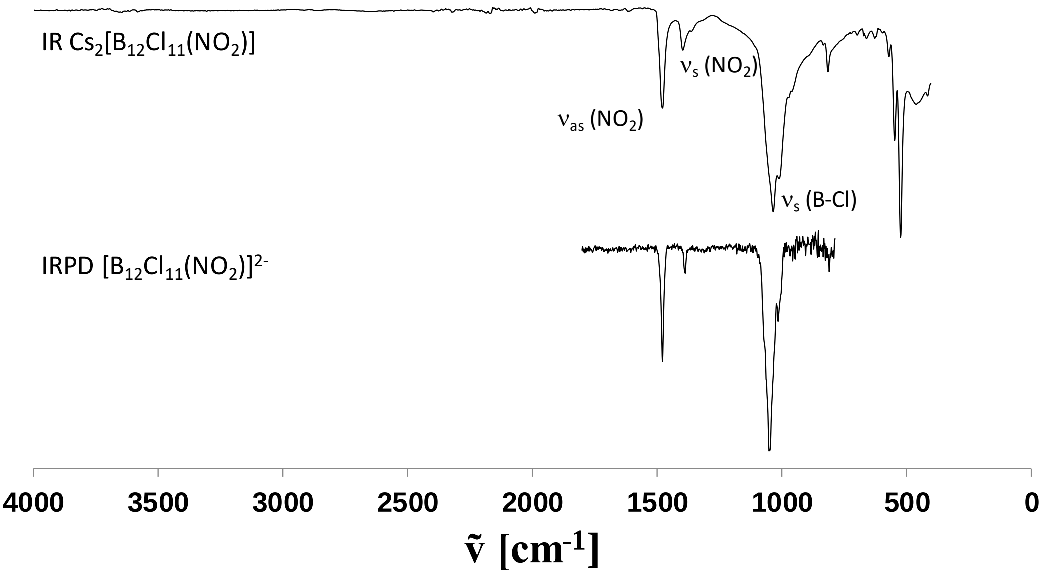

Figure S45: Comparison of the IR spectrum of $Cs_2[B_{12}Cl_{11}(NO_2)]$ (top) and the gas phase IRPD spectrum of the $[B_{12}Cl_{11}(NO_2)]^{2-}$ anion (bottom).

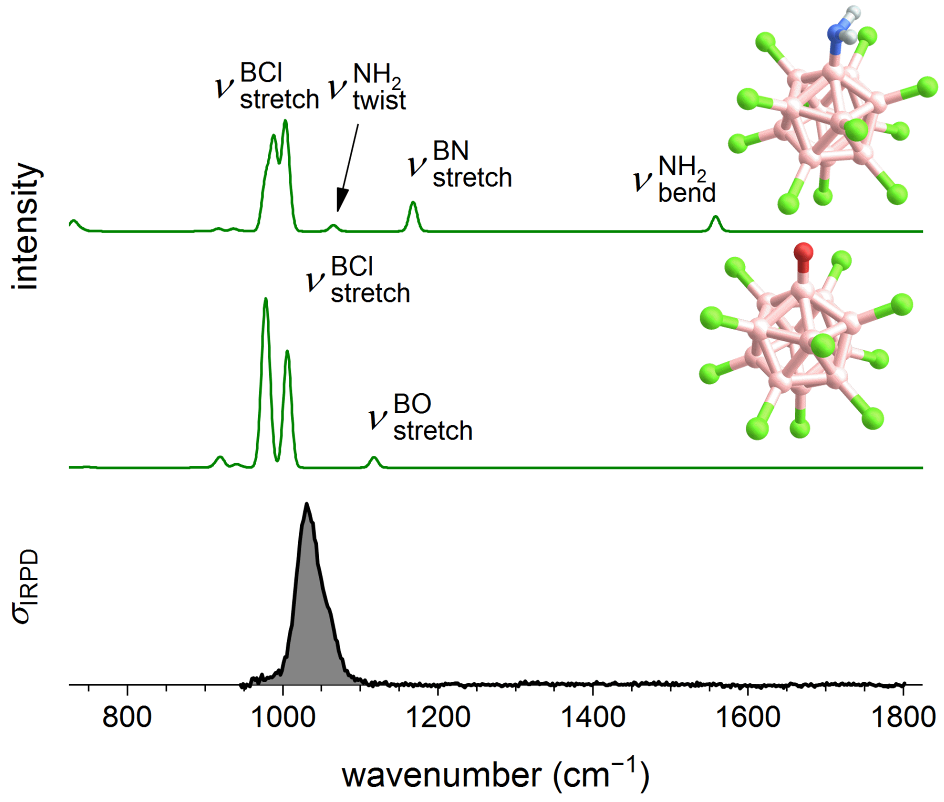

Figure S46: Comparison of the IR spectrum of $Cs_2[B_{12}Cl_{11}(NO_2)]$ (top) and the gas phase IRPD spectrum of the $[B_{12}Cl_{11}(NO_2)]^{2-}$ anion (bottom).

Table S13. Experimental observed band positions compared with theoretical frequencies and assignment for the IRPD spectra of $B_{12}Cl_{11}NO_2^-$, $B_{12}Cl_{11}O^-$ and $B_{12}Cl_{11}ONO^-$. Theoretical values are scaled by 0.968.

System	experimental	theory	assignment
$B_{12}Cl_{11}NO_2^-$	1478	1481	antisymmetric NO_2 stretch
	1388	1383	symmetric NO_2 stretch
	1047	1002	BCl stretch
	1015	975	BCl stretch
$B_{12}Cl_{11}O^-$	1031	1006/978	BCl stretch
$B_{12}Cl_{11}ONO^-$	1599	1611	NO stretch (free)
	1159	1133	BO stretch
	1044	1002/982	BCl stretch
	875	870	NO_2 bend
	788	793	NO stretch (bonded)

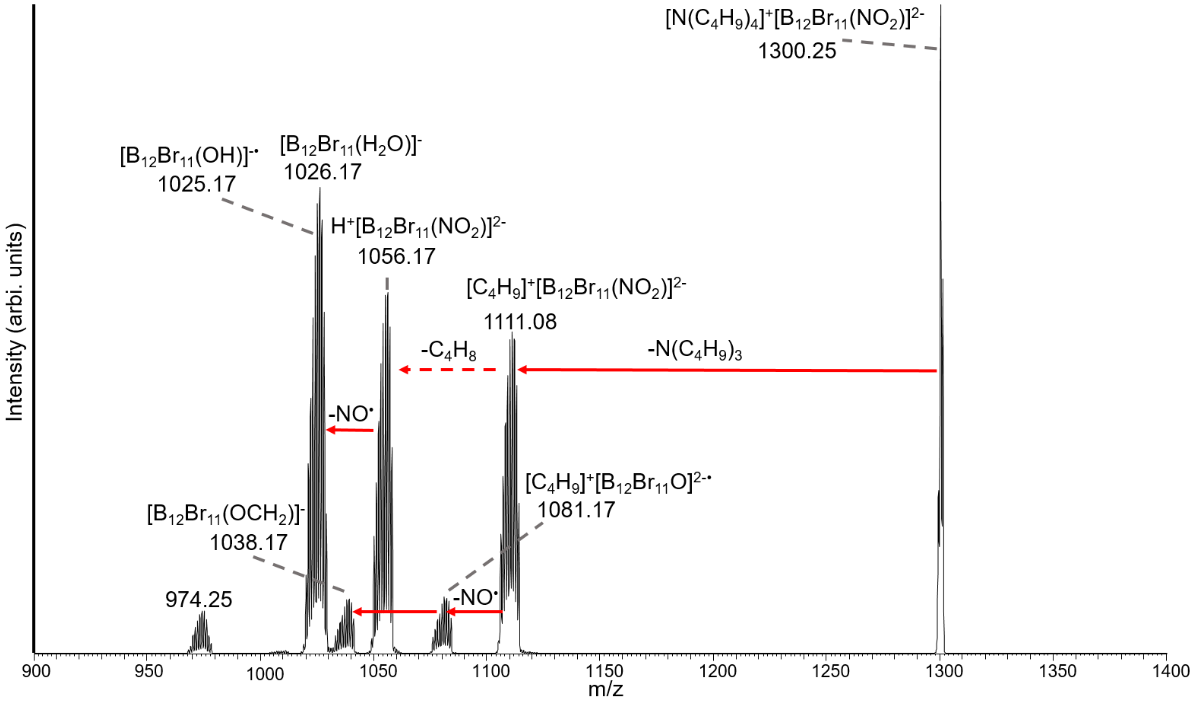

Figure S47: Isolation of all $[N(C_4H_9)_4]^+[B_{12}Br_{11}(NO_2)]^{2-}$ ions (isolation width m/z 10) and subsequent excitation by CID. Note that precursor isotopic patterns are often strongly disturbed in MS/MS experiments. The assignment of neutral losses is based on the experiment shown in Figure S48. The dotted arrow is indicating a side reaction. It should be mentioned that the signal intensity of $H^+[B_{12}Br_{11}(NO_2)]^{2-}$ is significantly higher in experiments in which $[N(C_4H_9)_4]^+[B_{12}Br_{11}(NO_2)]^{2-}$ has been isolated and excited by CID (this Figure and Figure S48) than in experiments in which $[C_4H_9]^+[B_{12}Br_{11}(NO_2)]^{2-}$ has been isolated and excited by CID (Figure S49). In case of isolating $[C_4H_9]^+[B_{12}Br_{11}(NO_2)]^{2-}$ the $NO^•$ loss is clearly favored as the main reaction and the alkene loss occurs only as a side reaction (see Figure S49). Probably, $H^+[B_{12}Br_{11}(NO_2)]^{2-}$ forms directly from $[N(C_4H_9)_4]^+[B_{12}Br_{11}(NO_2)]^{2-}$.

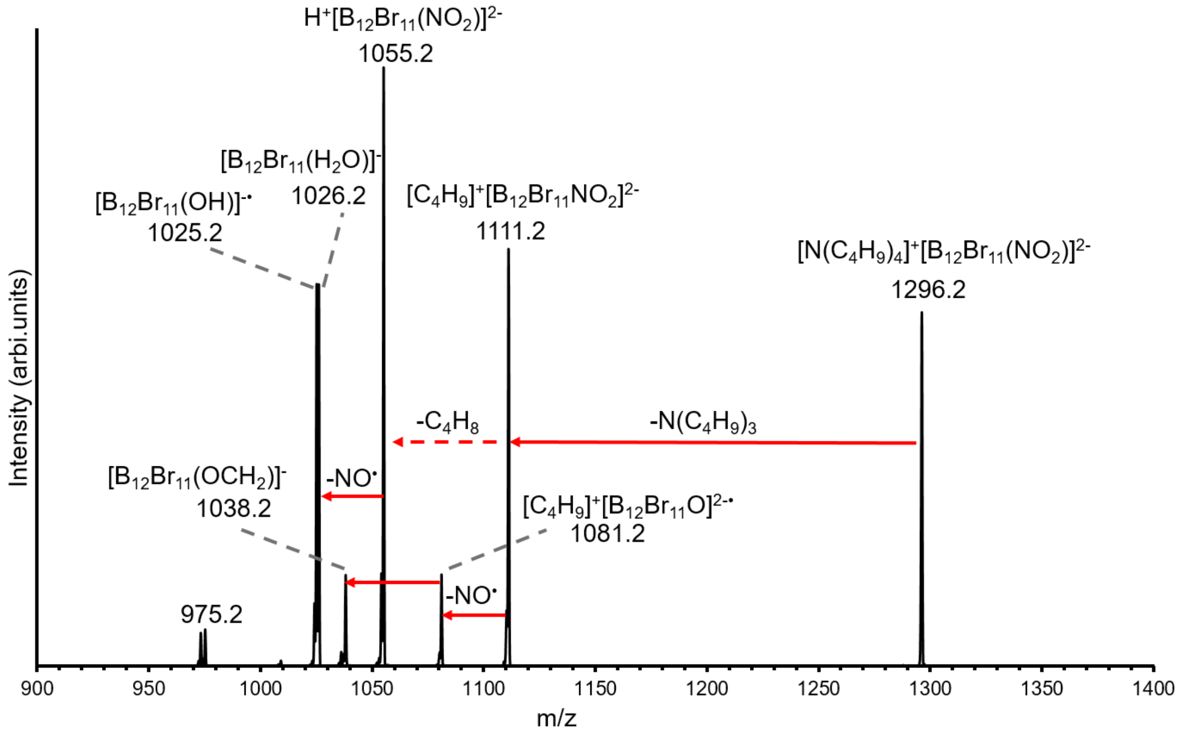

Figure S48: Isolation of all $[N(C_4H_9)_4]^+[B_{12}Br_{11}(NO_2)]^{2-}$ ions with the nominal mass 1296 (isolation width: m/z 1) and subsequent excitation by CID. Assigned fragmentation pathways (red arrows) are based on MS/MS experiments, see Figure S49-53.

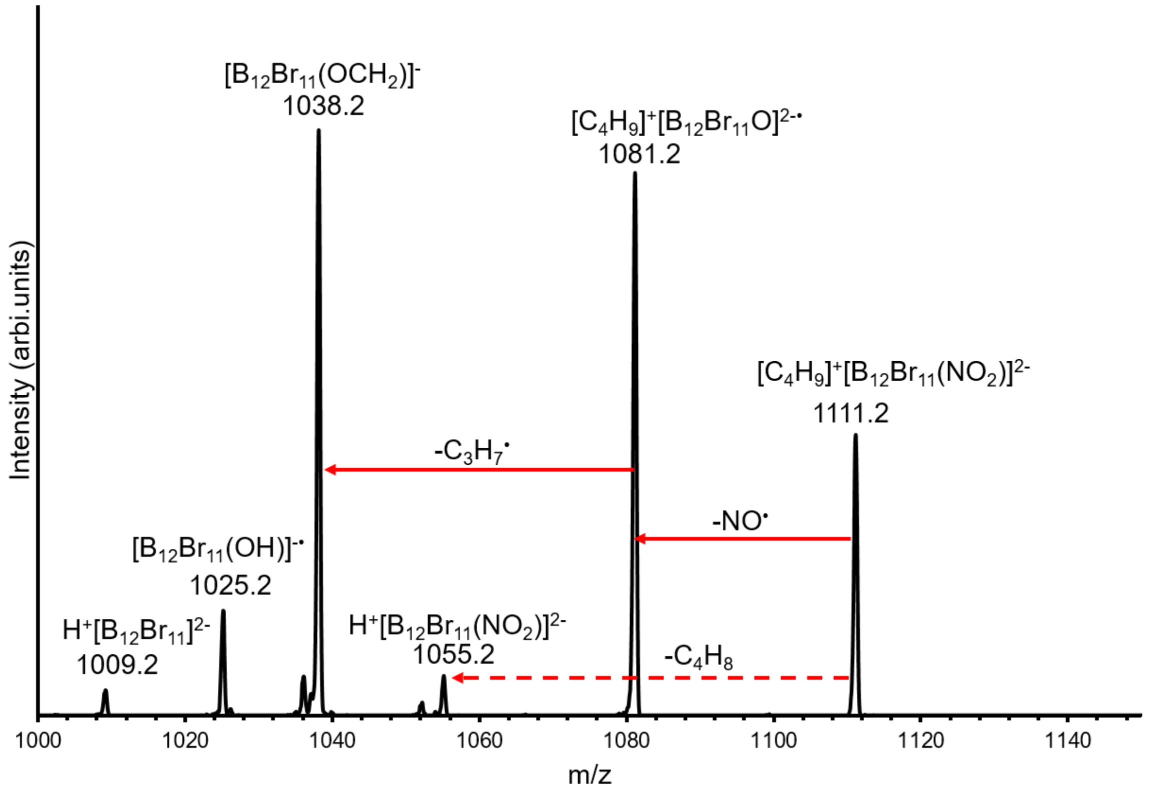

Figure S49: Isolation of all $[C_4H_9]^+[B_{12}Br_{11}(NO_2)]^{2-}$ ions with the nominal mass 1111 (isolation width: m/z=1) and subsequent excitation by CID. Assigned fragmentation pathways (red arrows) are based on MS/MS experiments, see Figure S50 to Figure S53.

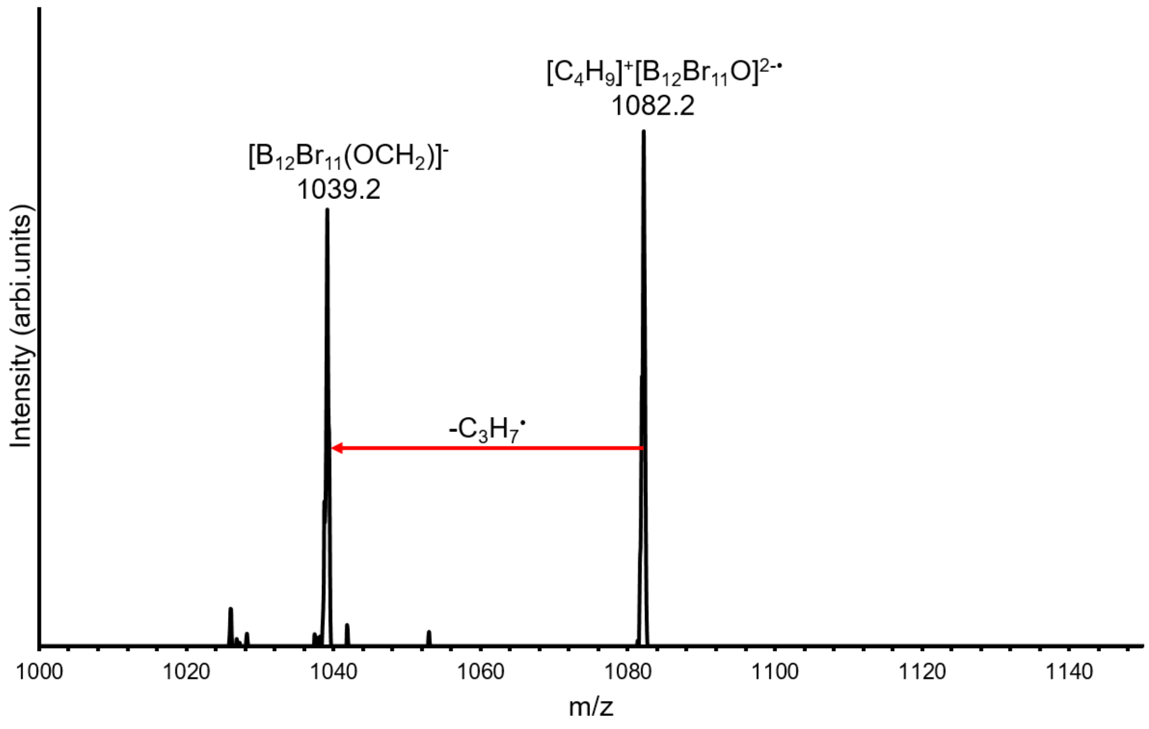

Figure S50: Isolation of all $[C_4H_9]^+[B_{12}Br_{11}O]^{2-\bullet}$ ions with the nominal mass 1082 (isolation width: m/z=1) and subsequent excitation by CID.

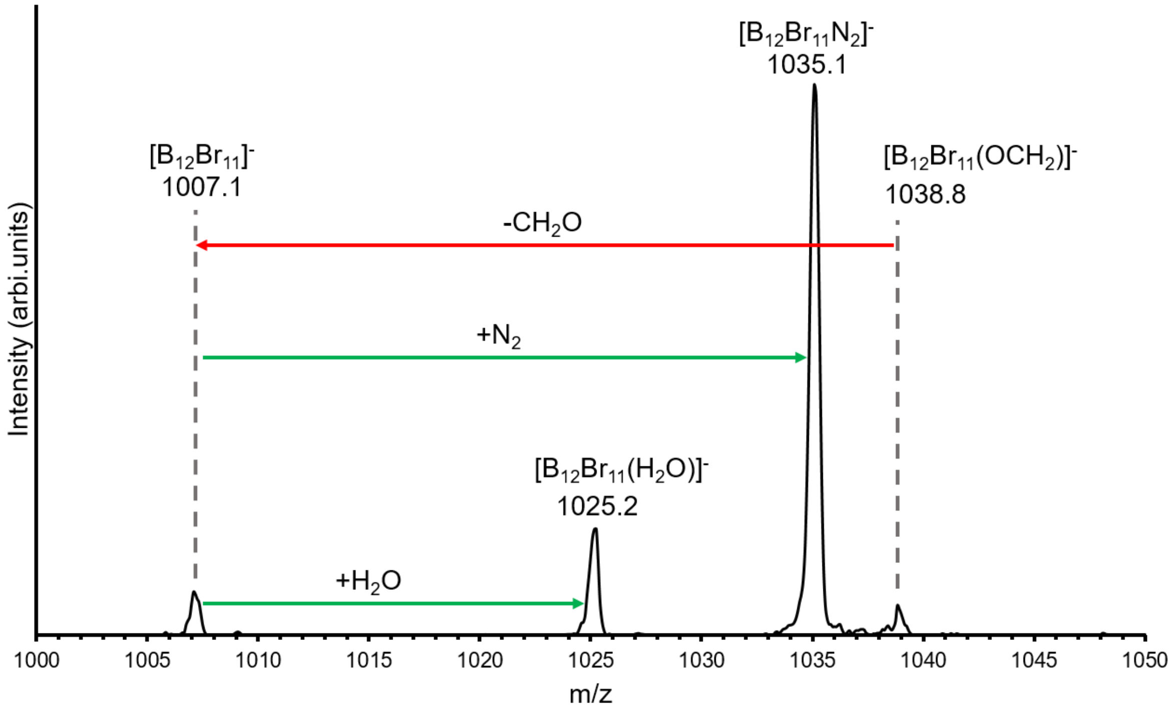

Figure S51: Isolation of all $[B_{12}Br_{11}(OCH_2)]^-$ ion with the nominal mass 1038 (isolation width: m/z=1) and subsequent excitation by CID.

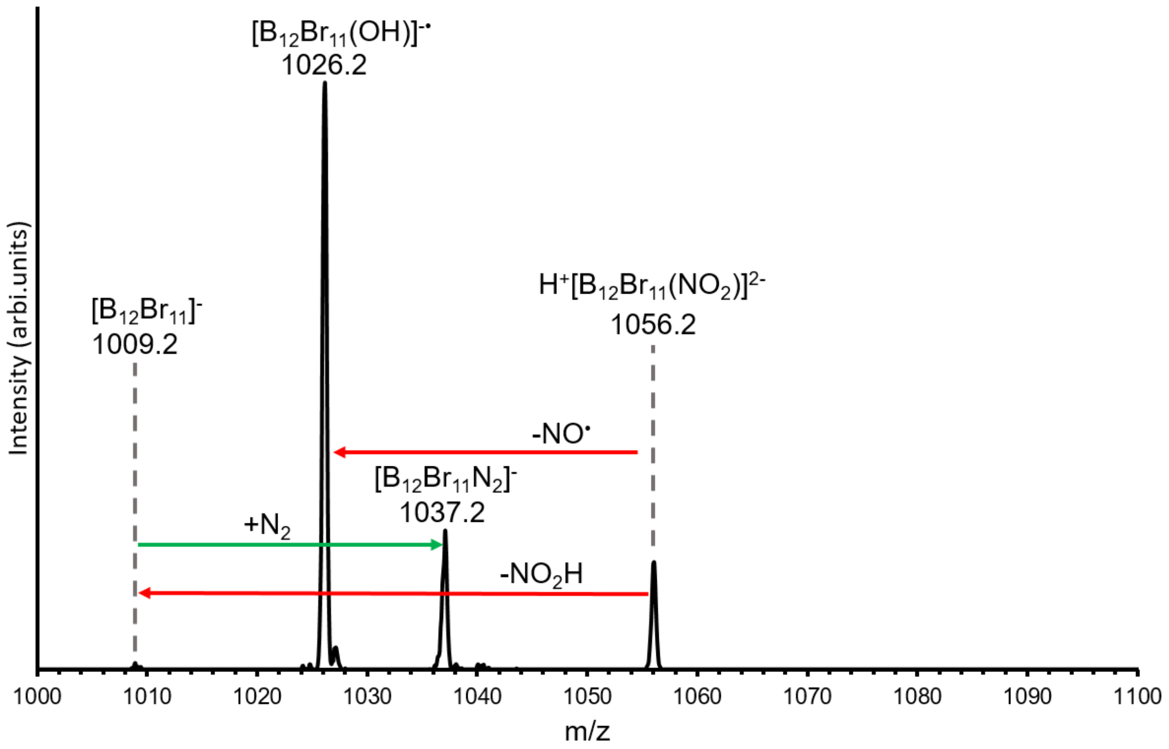

Figure S52: Isolation of all $H^+[B_{12}Br_{11}(NO_2)]^{2-}$ ions with the nominal mass 1056 (isolation width: m/z=1) and subsequent excitation by CID.

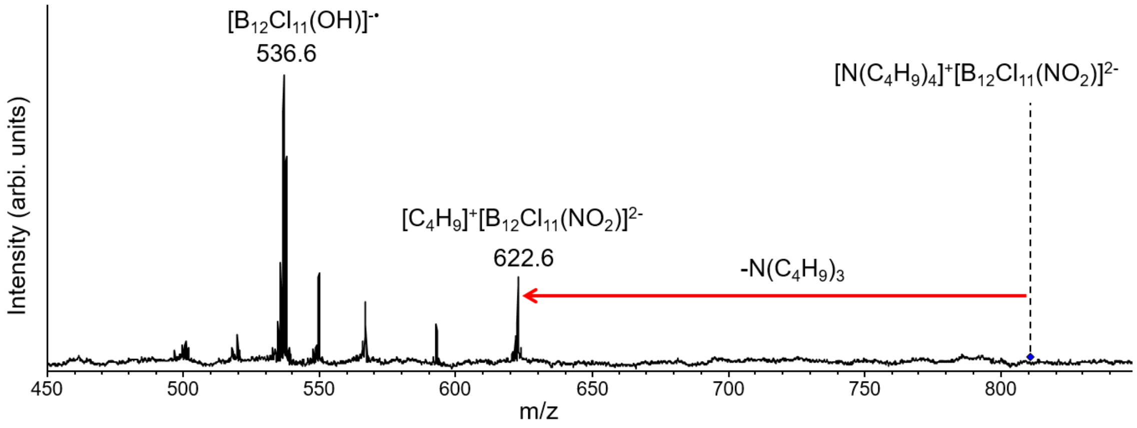

Figure S53: Isolation of all $[N(C_4H_9)_4]^+[B_{12}Cl_{11}(NO_2)]^{2-}$ ions with the nominal mass of 810 and subsequent excitation by CID. For reasons of clarity, the assignment of neutral losses is shown in Figure S54., which represents the area between m/z 480 and 640.

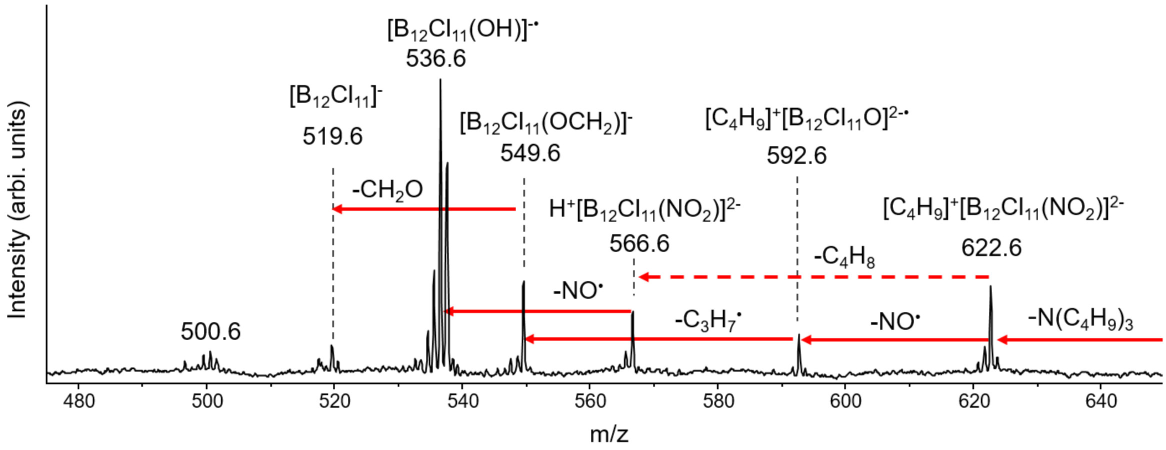

Figure S54: Isolation of all $[N(C_4H_9)_4]^+[B_{12}Cl_{11}(NO_2)]^{2-}$ ions with the nominal mass of 810 and subsequent excitation by CID. Only the area between m/z 480 and 640 is shown. Solid red arrows are indicating neutral losses. The dotted arrow is referring to the side reaction, see caption of Figure S47.

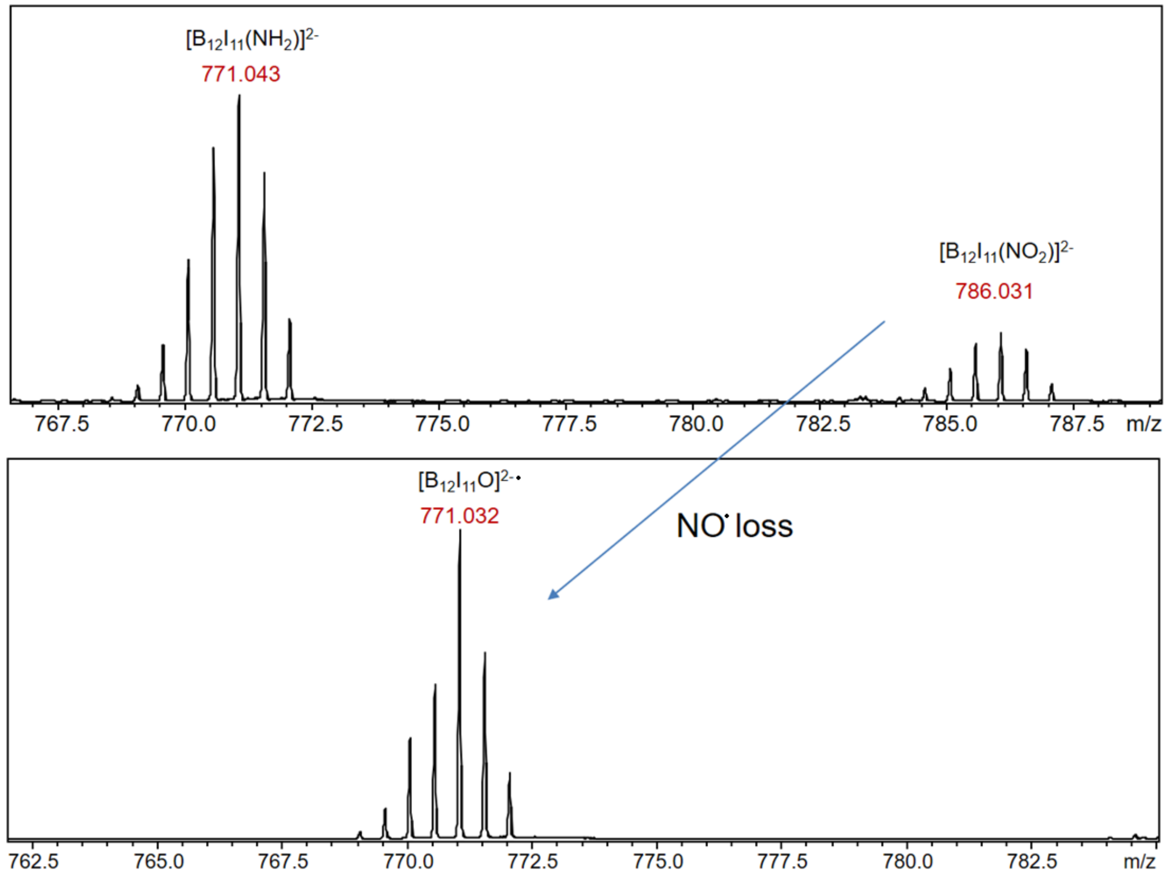

Figure S55: High resolution mass measurement (ESI(-)) of a sample containing a mixture of $[B_{12}I_{11}(NO_2)]^{2-}$ and $[B_{12}I_{11}(NH_2)]^{2-}$ (top). Fragmentation of the former yields $[B_{12}I_{11}O]^{2-\bullet}$ (bottom) which can be clearly distinguished from $[B_{12}I_{11}(NH_2)]^{2-}$.

S10 References

[1] V. Geis, K. Guttsche, C. Knapp, H. Scherer, R. Uzun, *Dalton Trans.* **2009**, 2687–2694.

[2] C. Bolli, J. Derendorf, C. Jenne, H. Scherer, C. P. Sindlinger, B. Wegener, *Chem. Eur. J.* **2014**, *20*, 13783–13792.

[3] E. V. Bukovsky, A. M. Pluntze, S. H. Strauss, *J. Fluorine Chem.* **2017**, *203*, 90–98.

[4] J. Holub, S. El Anwar, T. Jelínek, L. Fojt, Z. Růžičková, V. Šolínová, V. Kašička, D. Gabel, B. Grüner, *Eur. J. Inorg. Chem.* **2017**, *2017*, 4499–4509.

[5] a) M. J. Frisch, G. W. Trucks, H. B. Schlegel, G. E. Scuseria, M. A. Robb, J. R. Cheeseman, G. Scalmani, V. Barone, G. A. Petersson, H. Nakatsuji, X. Li, M. Caricato, A. V. Marenich, J. Bloino, B. G. Janesko, R. Gomperts, B. Mennucci, H. P. Hratchian, J. V. Ortiz, A. F. Izmaylov, J. L. Sonnenberg, Williams, F. Ding, F. Lipparini, F. Egidi, J. Goings, B. Peng, A. Petrone, T. Henderson, D. Ranasinghe, V. G. Zakrzewski, J. Gao, N. Rega, G. Zheng, W. Liang, M. Hada, M. Ehara, K. Toyota, R. Fukuda, J. Hasegawa, M. Ishida, T. Nakajima, Y. Honda, O. Kitao, H. Nakai, T. Vreven, K. Throssell, J. A. Montgomery Jr, J. E. Peralta, F. Ogliaro, M. J. Bearpark, J. J. Heyd, E. N. Brothers, K. N. Kudin, V. N. Staroverov, T. A. Keith, R. Kobayashi, J. Normand, K. Raghavachari, A. P. Rendell, J. C. Burant, S. S. Iyengar, J. Tomasi, M. Cossi, J. M. Millam, M. Klene, C. Adamo, R. Cammi, J. W. Ochterski, R. L. Martin, K. Morokuma, O. Farkas, J. B. Foresman, D. J. Fox, *Gaussian 16 Rev. A.03*, Wallingford, CT, **2016**; b) Perdew, Burke, Ernzerhof, *Phys. Rev. Lett.* **1996**, *77*, 3865–3868; c) C. Adamo, V. Barone, *J. Chem. Phys.* **1999**, *110*, 6158–6170; d) F. Weigend, R. Ahlrichs, *Phys. Chem. Chem. Phys.* **2005**, *7*, 3297–3305; e) Lee, Yang, Parr, *Phys. Rev. B* **1988**, *37*, 785–789; f) B. Miehlich, A. Savin, H. Stoll, H. Preuss, *Chem. Phys. Lett.* **1989**, *157*, 200–206; g) A. D. Becke, *J. Chem. Phys.* **1993**, *98*, 5648–5652; h) S. Grimme, S. Ehrlich, L. Goerigk, *J. Comput. Chem.* **2011**, *32*, 1456–1465;

[6] *Chemcraft - graphical software for visualization of quantum chemistry computations. https://www.chemcraftprog.com.*

[7] M. D. Hanwell, D. E. Curtis, D. C. Lonie, T. Vandermeersch, E. Zurek, G. R. Hutchison, *J. Cheminform.* **2012**, *4*, 17.

[8] a) N. Heine, K. R. Asmis, *Int. Rev. Phys. Chem.* **2015**, *34*, 1–34; b) N. Heine, K. R. Asmis, *Int. Rev. Phys. Chem.* **2016**, *35*, 507;

[9] a) M. Brümmer, C. Kaposta, G. Santambrogio, K. R. Asmis, *J. Chem. Phys.* **2003**, *119*, 12700–12703; b) D. J. Goebbert, T. Wende, R. Bergmann, G. Meijer, K. R. Asmis, *J. Phys. Chem. A* **2009**, *113*, 5874–5880;

[10] W. R. Bosenberg, D. R. Guyer, *J. Opt. Soc. Am. B* **1993**, *10*, 1716.

[11] a) R. T. Boeré, C. Bolli, M. Finze, A. Himmelspach, C. Knapp, T. L. Roemmele, *Chem. Eur. J.* **2013**, *19*, 1784–1795; b) R. T. Boeré, J. Derendorf, C. Jenne, S. Kacprzak, M. Keßler, R. Riebau, S. Riedel, T. L. Roemmele, M. Rühle, H. Scherer, T. Vent-Schmidt, J. Warneke, S. Weber, *Chem. Eur. J.* **2014**, *20*, 4447–4459;

[12] G. M. Sheldrick, *Acta Cryst.* **2015**, *A71*, 3–8.

[13] O. V. Dolomanov, L. J. Bourhis, R. J. Gildea, J. A. K. Howard, H. Puschmann, *J. Appl. Cryst.* **2009**, *42*, 339–341.

[14] G. M. Sheldrick, *Acta Cryst.* **2015**, *C71*, 3–8.
